# Spontaneous switching in a protein signalling array reveals near-critical cooperativity

**DOI:** 10.1038/s41567-025-03158-3

**Published:** 2026-01-29

**Authors:** Johannes M. Keegstra, Fotios Avgidis, Evan Usher, Yuval Mulla, John S. Parkinson, Thomas S. Shimizu

**Affiliations:** 1 AMOLF, Amsterdam, The Netherlands; 2 Institute for Environmental Engineering, ETH Zurich, Zurich, Switzerland; 3 School of Biological Sciences, University of Utah, Salt Lake City, UT USA

**Keywords:** Biological physics, Computational biophysics, Supramolecular assembly, Motility, Phase transitions and critical phenomena

## Abstract

Cooperative interactions within large protein assemblies are crucial for cellular information processing. However, direct observations of cooperative transitions have been limited to compact molecular assemblies. Here we report the in vivo measurements of spontaneous discrete-level transitions in the activity of an entire *Escherichia coli* chemosensory array—an extensive membrane-associated assembly comprising thousands of molecules. Finite-size scaling analysis of the temporal statistics reveals nearest-neighbour coupling strengths within 3% of the Ising phase transition, indicating that chemosensory arrays are poised at criticality. We also show how *E. coli* exploits both static and dynamic disorder, arising from chemoreceptor mixing and sensory adaptation, respectively, to temper the near-critical dynamics. This tempering eliminates detrimental slowing of response while retaining substantial signal gain as well as an ability to modulate physiologically relevant signal noise. These results identify near-critical cooperativity as a design principle for balancing the inherent trade-off between response amplitude and response speed in higher-order signalling assemblies.

## Abstract (web summary)

Many biological systems appear to organize their dynamics close to a critical point. Now it is shown that the protein array mediating *Escherichia coli* chemosensing is near-critical, enabling large signal amplification without compromising response speeds.

## Main

Large protein assemblies at the heart of many cell signalling processes exhibit varying degrees of structural and dynamical order, from liquid-like granules^1–3^ to solid-like arrays^4–6^. Recent experiments are revealing how phase transitions that lead to the formation of liquid-like assemblies—discontinuous phase transitions analogous to the condensation of gas into liquid—are used by cells to implement various information processing tasks, such as signal initiation and confinement^7^, kinetic proofreading^8^ and noise control^9^. Theory also predicts that different phase transitions can occur within solid-like assemblies—continuous phase transitions, analogous to the ordering of magnetic spins in a ferromagnet at low temperatures—arising from conformational interactions between protein subunits^10^. However, requirements for continuous phase transitions are more stringent than those for discontinuous phase transitions—they occur only at a special point in phase space—a critical point. It remains unclear if such critical transitions occur in signalling assemblies and, if so, how they constrain or contribute to the functional design of protein assemblies. Elucidating the physical principles of signalling assemblies would not only advance our understanding of cell signalling in nature but also provide new design principles for the rational design of synthetic protein circuits^11–14^.

Recent structural studies are revealing an increasing number of solid-like signalling assemblies with a high degree of spatial order, where subunit proteins are arrayed in a regular pattern. The repertoire of such crystal-like assemblies reported so far is diverse in both form and function, and includes one-dimensional filament-like assemblies found in cellular homeostasis and inflammation signalling^15,16^, protein rings that mediate the control of cell motility and apoptosis^17,18^, as well as two-dimensional arrays involved in chemosensing, neuromuscular control and innate immune responses^19–22^. Yet, despite exciting advances in resolving the ultrastructure of these large assemblies^23,24^, the mechanistic design principles of their signalling function remain challenging to address experimentally. Of particular interest is the ability of signalling assemblies to perform signal processing by way of cooperative interactions between assembly subunits. In contrast to the compact oligomeric signalling complexes of fixed size, these extensive structures tend to assemble through open-ended polymerization and/or multivalent interactions, making them inherently variable in both size and composition^5^. The resulting polydispersity and stoichiometric diversity tend to mask their true signalling dynamics^25,26^, as well as their size and composition dependencies^6,27^, both in vivo and in vitro. An ideal functional assay would, therefore, interrogate assembly level dynamics *in singulo*—at the level of an individual assembly—but experimental realization has remained elusive.

**Fig. 1:**
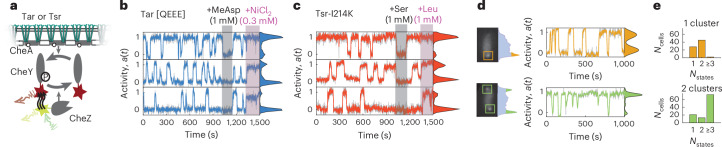
*In singulo* measurements of chemosensory array dynamics reveal two-state switching fluctuations. **a**, Illustration of single-cell FRET assay to measure the kinase activity^34^, with a schematic of the side view of a membrane-associated chemosensory array of chemoreceptors (either Tar or Tsr). The kinase (CheA) within the chemosensory array phosphorylates CheY (CheY-P), whereas CheZ dephosphorylates CheY. The FRET signal (black arrows) between fluorescently labelled CheZ and CheY, measured from the emitted fluorescence intensities (red and yellow arrows) is proportional to the activity of the chemosensory array. **b**,**c**, Activity time series from single-cell FRET experiments that reveal two-state switching in the absence of any chemoeffector, of representative cells without adaptation enzymes expressing only the chemoreceptor Tar [QEEE] (**b**; blue) or the chemoreceptor Tsr-I214K (**c**; red). Cells are exposed to the measurement buffer (MotM) for most of the experiment. To obtain the minimum and maximum FRET levels, cells are exposed to an attractant stimulus (Tar: 1 mM α-methylaspartate (MeAsp); Tsr: 1 mM l-serine (Ser), grey bands) and a repellent stimulus (Tar: 0.3 mM NiCl_2_; Tsr: 1 mM l-leucine (Leu), purple bands). These levels were then used to normalize the activity time series of each cell between zero and unity. Example time series other than the two-state one are shown in Extended Data Fig. 1. **d**, Chemosensory cluster organization from CheZ localization (left) and the corresponding activity time series (right, raw in grey, 6 s (averaged); in colour) and histograms (on the right margin) of activity determined by FRET, for a representative cell expressing only the chemoreceptor Tsr-I214K, with a single visible cluster exhibiting two-state switching (orange) and a representative cell with two visible clusters exhibiting multistate switching (green). Additional time series and cluster organization are shown in Extended Data Fig. 2. **e**, Histograms of the number of states in switching cells, for cells with one visible cluster (top, orange) and for cells with two visible clusters (bottom, green).

Here we report in vivo FRET experiments that resolve this challenge for the chemosensory array of *Escherichia coli*, a canonical two-dimensional signalling assembly lining the cytoplasmic membrane, which allows motile bacteria to bias their random-walk swimming trajectories to navigate chemical environments^28–30^. This higher-order assembly comprises thousands of receptor, kinase and scaffolding molecules arranged in a well-defined lattice structure^31–33^ and, thus, serves as a paradigm for signalling in two-dimensional protein assemblies. The array integrates input signals (chemoeffector ligand concentrations) with a negative feedback signal (covalent modification state of receptors) to generate a signal output (activity of the kinase, CheA) that can be followed in real time by an intermolecular Förster resonance energy transfer (FRET) system^28^. By labelling two downstream proteins, the response regulator CheY, which is phosphorylated by CheA, and its phosphatase CheZ, which dephosphorylates CheY, a FRET read-out proportional to the kinase activity can be obtained within live cells^28^ (Fig. 1a). Our strategy leverages recent advances in extending this in vivo FRET technique to the single-cell level^34,35^, to achieve functional measurements of individual arrays within live cells. Although this FRET system provides a whole-cell measurement that integrates the kinase output of all arrays within each cell, the number of arrays per cell is highly variable^36^ and in a substantial minority of cells, nearly all receptors and other array-component proteins are assembled within one dominant large array^37^. Thus, by searching for cells in which signalling is dominated by a single large array, we aimed to achieve *in singulo* FRET measurements of functional array output.

## Spontaneous two-state switching in chemosensory arrays

**Extended Data Fig. 1:**
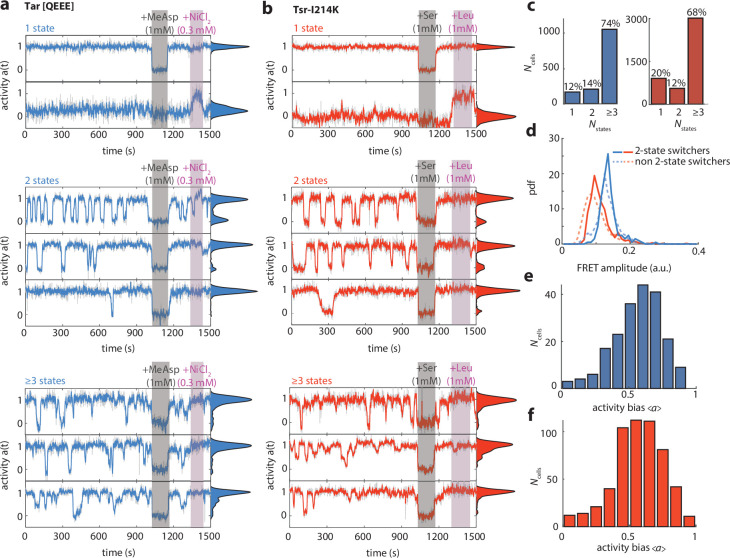
Classification of fluctuation phenotypes and temperature dependence in cells expressing chemoreceptors Tar or Tsr. (**a**). Activity time series of eight representative individual cells expressing only the chemoreceptor Tar [QEEE] without adaptation enzymes (pVS120/TSS1964). Temporal fluctuations during flow of measurement buffer are recorded prior to stimulation by saturating pulses of attractant (1 mM MeAsp) (gray) and repellent (0.3 mM NiCl_2_) (pink). Cells with one, two, and three or more stable states are shown. On each raw time series (gray), a 3 s moving average filter was applied and the resulted time series are superimposed (blue), shown together with associated activity histograms of the filtered time series. (**b**) As in (**a**), but with cells expressing only Tsr-I214K in the same background (pPA114/TSS1964), and stimulated with an attractant stimulus of 1 mM l-Serine (Ser) and a repellent stimulus of 1 mM Leucine (Leu). (**c**) Histogram of number of states of total 1414 Tar [QEEE] cells (left, blue) and 4446 Tsr-I214K cells (right, red). (**d**) Histrograms of maximum FRET amplitude of 1414 cells expressing Tar [QEEE] (left, blue) and 4446 cells expressing Tsr-I214K (right, red). Cells exhibiting two-state switching behavior (solid lines) have approximately equal or higher maximum FRET signals (and hence kinase activity) compared to the rest of the cells (dashed lines), ruling out that switching cells have an anomalously low expression of chemoreceptor array components. (**e**) Histogram of activity bias 〈*a*〉 of cells exhibiting two-state switching behavior, for Tar [QEEE]. Activity bias (*a* ≈ 1) is defined as the sum of all residence time intervals for active (up) state (*a* ≈ 1) divided by the sum of all residence time intervals. (**f**) as in e, but for Tsr-I214K.

To identify cells whose chemoreceptor population is concentrated into a single large array, we focused on fluctuations in the kinase output that are observable by FRET at the single-cell level. In wild-type (WT) *Escherichia coli* cells, the stochastic kinetics of the reversible covalent modification reactions (methylation/demethylation) mediated by the adaptation system are known to drive temporal fluctuations in kinase activity under constant environmental conditions^34,35,38^. Surprisingly, however, previous single-cell FRET studies identified the largest fluctuations in engineered genetic mutants devoid of the adaptation system and expressing only one of the five chemoreceptor species^34,35^, with a subpopulation of cells exhibiting two-level (all-ON/all-OFF) fluctuations in kinase output as the input ligand signal was held constant, at a sub-saturating level^34^ (Supplementary Note 1). If these fluctuations were driven by the intrinsic dynamics of the array, such synchronized two-level stochastic switching of the entire kinase population would be expected only if whole-cell signalling were dominated by a single dominant array. However, because an external ligand was present in those experiments, the possibility remained that the observed output switches were caused by spurious fluctuations in the external chemoeffector signal. We therefore began by testing whether two-level fluctuations occur in the absence of any external ligand stimulus by exploiting known genetic modifications of chemoreceptors that mimic the effects of methylation/demethylation, and can modify the chemoreceptor activity bias and maintain otherwise normal signalling function^39^. We tuned down the activity bias by expressing the aspartate receptor Tar in the QEEE covalent modification state (from QEQE in WT Tar), which yields an intermediate activity bias without the addition of chemoeffectors (Supplementary Note 1 and Extended Data Fig. 1e)^39^. This allowed us to measure temporal fluctuations by single-cell FRET recordings in thousands of individual cells (Fig. 1b and Extended Data Fig. 1a). In 204 out of 1,414 cells obtained from 19 FRET experiments, we observed two-level switching between a high- and low-activity state (>65% of transitions showing activity-level changes of >0.7). Spontaneous switching in the absence of exogenous ligands was not specific to Tar receptors, as we observed a similar switching behaviour (two-state switching in 548 out of 4,446 cells across 44 experiments) in analogous experiments with Tsr-I214K, a single-residue replacement mutant of the serine receptor Tsr within its ‘control cable’ region^40^ with a downshifted activity bias similar to that of Tar [QEEE] (Fig. 1c and Extended Data Fig. 1b).

**Extended Data Fig. 2:**
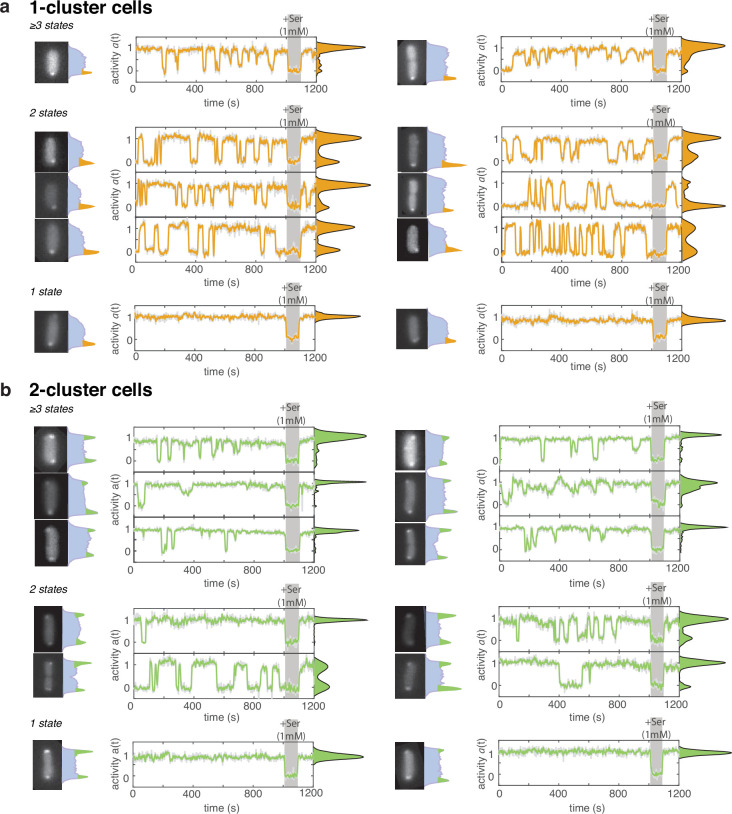
Further examples of fluctuation phenotypes of cells with one or two large CheZ-YFP clusters. Examples from the same experiment in Fig. 1d with reduced FRET-pair expression. (**a**) FRET time series of cells exhibiting one large Tsr-I214K receptor cluster and no adaptation enzymes (pPA114/TSS1964), shown alongside donor (YFP-channel) fluorescence images and intensity profile along the long axis of the cell. Cells demonstrating one, two, and three or more stable states are shown. In these experiments FRET pair expression was induced at lower levels than standard conditions (which are optimized for FRET photon budget), in order to decrease cytoplasmic levels of CheZ-YFP fluorescence and therefore allow detection of large chemosensory arrays through detection of CheZ-YFP clusters. Cells are exposed to measurement buffer and then to an attractant stimulus of 1 mM l-Serine (gray). On each raw time series (gray), a 6 s moving average filter was applied and the resulted time series are superimposed (orange), shown together with marginal histograms of the filtered activity time series. (**b**) As in (**a**), but for cells exhibiting two large Tsr-I214K receptor clusters. For histograms of classified fluctuation phenotypes, for both one-cluster (**a**) and two-cluster (**b**) cells, see Fig. 1e.

**Extended Data Fig. 3:**
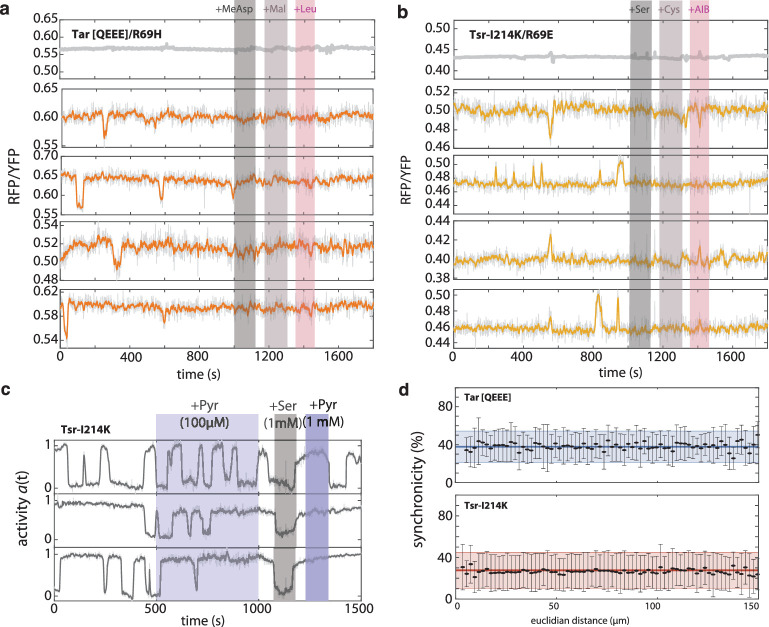
Switching behavior does not stem from ligand sensing or metabolic fluctuations. (**a**) FRET response from cells expressing only the receptor Tar [QEEE]/R69H, a double mutant receptor with impaired ligand binding sites, exposed to buffer or different chemoeffectors (shaded regions). The following saturating doses of chemoattractants were tested: 1 mM MeAsp; Mal: 1 mM Maltose; Leu: 1 mM Leucine. Measurement at 25 ^∘^C. Because ligand binding is abolished, the minimum and maximum FRET levels (determined by adding attractant and repellent, respectively) cannot be extracted and thus FRET time series are shown in (non-normalized) raw RFP/YFP intensity units. Shown are the population-averaged response (top) and 4 example single-cell time series (red). On each raw single time series (gray), a 7 s moving average filter was applied and the resulting time series are superimposed (red). (**b**) As in (**a**), but for cells expressing only Tsr-I214K/R69E, a double mutant receptor with impaired ligand binding sites, exposed to buffer or different chemoeffectors (shaded regions). The following saturating doses of chemoattractants were tested: Ser: 1 mM l-Serine; Cys: 10 mM Cysteine; AIB: 10 mM *α*-aminoisobutyric acid. Measurement at 35 ^∘^C. Shown are the population-averaged time series (top) with 4 example single-cell time series (yellow). On each raw time series (gray), a 7 s moving average filter was applied and the resulting time series are superimposed (yellow). (**c**) Switching in the presence of pyruvate. Intracellular pyruvate levels have recently been shown to exhibit large fluctuations in *E. coli* (ref. ^108^). Suppressing those fluctuations in cells expressing only the receptor Tsr-I214K by adding 100 *μ*M pyruvate (purple shaded region) did not interrupt switching, thus establishing that switching does not stem from such metabolic fluctuations. Pyruvate is not a chemoeffector, as evident by the absence of response to a 1 mM pyruvate stimulus (dark purple shaded region). (**d**) (Top) Measure of synchronicity between 2-state switching cells expressing Tar [QEEE]. For each independent experiment, one reference cell is chosen, and the time of incidence of its switching events is compared with the time of incidence of the switching events in the rest of the cells within a window of ± 10 seconds. A synchronicity of 100% corresponds to all switching events coinciding within the same respective window. The process is then repeated across all cells within the same experiment and then, for all 19 experiments, totaling 204 cells. A control was generated by repeating the process one more time, where switching events across all cells from the same experiment are shuffled and a number of events, corresponding to the number of events in the reference cell, are drawn randomly and with replacement. Then, the time of incidence of the reference cell’s switching events is compared to random set of events (solid blue line represents average, shaded area represents standard deviation). The synchronicity of switching events between cells within the same experiment can be fully explained by random synchronization of switching events. (Bottom) Same analysis for cells expressing Tsr-I214K, across 44 experiments (548 cells).

**Extended Data Fig. 4:**
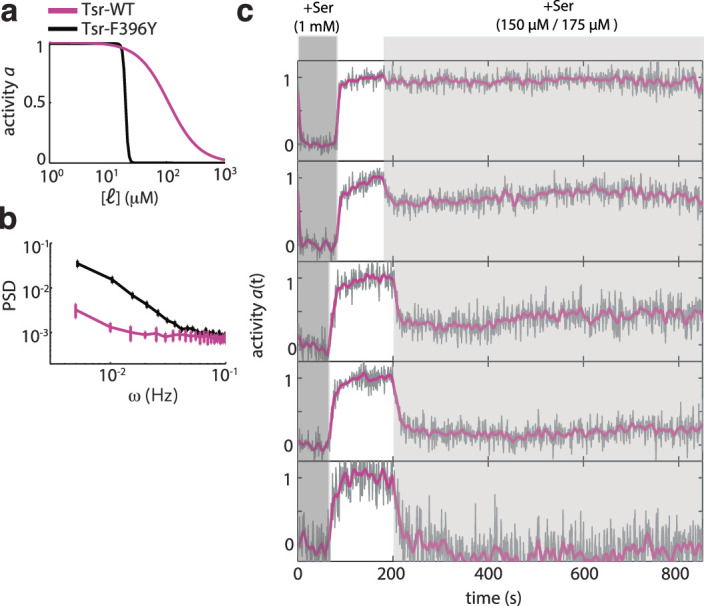
Absence of switching behavior in cooperativity defect mutant cells. (**a**) Hill curves illustrating the difference in cooperativity of the responses to Serine between wildtype Tsr (black) and Tsr-F396Y (purple). The dose–response parameters are the average single-cell fit values obtained from single-cell FRET experiments (81 cells) on both genotypes in cells lacking other chemoreceptors and the adaptation enzymes (pPA114/TSS1964). (**b**) Power spectral density (PSD) estimates for temporal signal fluctuations from cells expressing wildtype Tsr and Tsr-F396Y. Shown are the average of the single-cell PSD estimates from a single experiment (49 cells), error bars represent standard error of the mean (s.e.m.). (**c**) Example single-cell time series for a FRET experiment with cells expressing only Tsr-F396Y. At the start of the experiment, a saturating concentration ([*ℓ*]=1 mM l-serine, dark grey) is applied for a short time. After flushing buffer ([*ℓ*]=0, white), an intermediate concentration ([*ℓ*]=150-175 *μ*M l-serine, light grey) is sustained for approximately 10 minutes. 5 representative single-cell time series, each normalized to its activity level before adding the first stimulus are shown. The top two curves are taken from an experiment (34 cells) responding to 150 *μ*M l-serine, bottom three curves from a second experiment (49 cells) responding to 175 *μ*M l-serine. To the unfiltered data (gray) a 10-second moving average filter is applied and superimposed (purple).

Spontaneous two-state switching is a hallmark of allosteric signalling complexes such as ion channels^41^, but their observation requires measurements at the level of individual complexes; ensemble-averaged experiments cannot resolve the switching dynamics because the timings of switching events are uncorrelated and their dynamics are averaged out. To further test whether the observed two-level switches represent the behaviour of individual chemosensory arrays, we performed additional single-cell FRET experiments at a reduced expression level of the CheY/CheZ FRET pair. The attenuated cytoplasmic fluorescence in these experiments allowed the detection of chemosensory arrays as the intensity peaks of CheZ-YFP fluorescence, due to the well-established phenomenon of CheZ clustering at chemosensory arrays^42,43^, and the number of stable kinase output states could be determined through FRET experiments on the same individual cell (Fig. 1d and Extended Data Fig. 2). We found that cells that exhibited only a single detectable cluster predominantly demonstrated only one or two stable activity states, whereas cells with two clusters typically demonstrated three or more states (Fig. 1d and Extended Data Fig. 2). Tests with mutants deficient in either ligand binding or response cooperativity, as well as tests under a metabolic perturbation, further supported the idea that the observed switches are driven by intrinsic stochastic dynamics of the array, rather than extrinsic fluctuations (Extended Data Figs. 3 and 4 and Supplementary Note 1). Collectively, these results strongly support the idea that two-level fluctuations observed in our FRET experiments reflect cooperative signalling dynamics within a single dominant array.

**Fig. 2:**
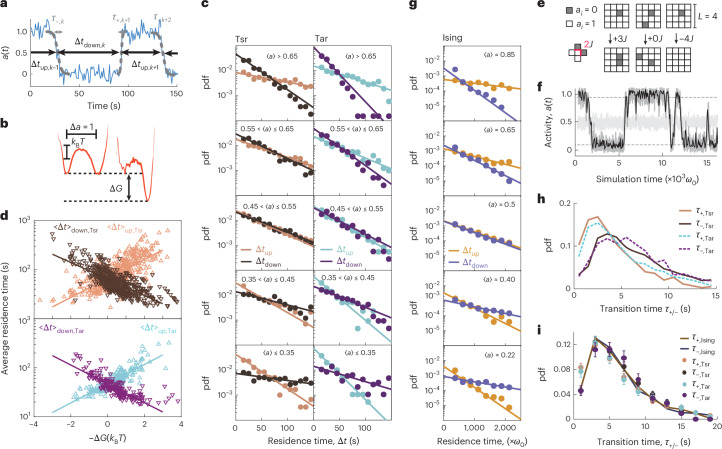
Temporal statistics of switching events are well described by a two-dimensional Ising model. **a**, Definitions of residence times Δ*t*_up,down_ and transition times *τ*_+,−_, determined routinely for each switching event (Methods). **b**, Coarse-grained energy landscape along the array-activity coordinate *a* (estimated as the negative logarithm of the activity histogram) based on the selected time series with 〈*a*〉 ≈ 0.5 (15 cells; left) and 〈*a*〉 ≈ 0.9 (12 cells; right) from cells expressing Tsr-I214K. Horizontal scale bar indicates the reaction coordinate Δ*a* = 1, vertical scale bar shows the energy (in *k*_B_*T*) and the dashed lines indicate the free energy difference Δ*G* between the high- and low-activity state. **c**, Histogram of residence times from experiments. Residence times for Tsr-I214K (left) and Tar [QEEE] (right), with each event sorted by the activity bias of the corresponding cell (colours as in **d**). In each panel, data (points) are shown together with fits to single exponential functions (solid lines). Fit parameters and number of data points are shown in Supplementary Tables 3, 5, 12 and 13. pdf, probability density function. **d**, Mean residence times per cell as a function of the energy bias Δ*G* between the high- and low-activity state for all cells expressing Tsr-I214K (top) and Tar [QEEE] (bottom). Fit parameters and number of data points are shown in Supplementary Table 7. **e**, Conformational spread model of the chemosensory array activity with size *L* × *L*, in which each individual unit can switch between the activity states of 1 (white) or 0 (dark). A difference in neighbouring spins is associated with an energy cost of *J*, shown for three different transitions on a lattice with size *L* = 4 in the absence of an external field (*H* = 0). **f**, Example activity time series obtained by simulating dynamics on a strongly coupled (*L* = 26, *J* = 0.4625*k*_B_*T*, dark grey) and weakly coupled (*L* = 20, *J* = 0.2375*k*_B_*T*, light grey) lattice. The strongly coupled lattice exhibits stochastic switches between two activity levels (dashed lines). The simulated time series was downsampled to approximate the experimental acquisition frequency (solid black line). **g**, Same data as in **c**, but histograms of the residence times from the simulated time series with *L* = 12 and *J* = 0.5*k*_B_*T*, sorted by the activity bias generated by an applied external field *H*. Fit parameters and number of data points are shown in Supplementary Tables 8, 10, 14 and 15. **h**, Histograms of transition times for Tsr-I214K (solid lines) and Tar [QEEE] (dashed lines). Mean transition times and number of data points are shown in Supplementary Tables 4 and 6. **i**, Transition times from simulated two-state time series (*N* = 12, *J* = 0.5*k*_B_*T*, varying *H*). To approximate the experimental signal-to-noise ratio (Supplementary Fig. 6), Gaussian white noise was added to the simulated time series. Mean simulated transition times and number of data points are shown in Supplementary Tables 9 and 11. Experimental (points) and simulated (solid lines) histograms are scaled horizontally to have mean transition time of cells expressing Tsr, for comparison. Error bars represent 95% confidence intervals obtained through bootstrap resampling.

To consider the physical mechanism underlying this long-range cooperativity, we analysed the temporal statistics of switching fluctuations (Fig. 2a) by extracting the time interval between switching events, hereafter called the residence times Δ*t*_up_ and Δ*t*_down_ for time spent in the up (*a* ≈ 1) and down (*a* ≈ 0) states, respectively, as well as the duration of the activity transient on switching, hereafter called the transition times *τ*_+_ and *τ*_−_ for upward and downward switches, respectively (Fig. 2a). We first interpreted these data as a barrier-crossing stochastic process in which the up and down states correspond to wells within an energy landscape (Fig. 2b), the shape of which could be approximated from the observed activity time series histograms with the free energy difference Δ*G* (in units of the thermal energy *k*_B_*T*) between the up and down states determined by the activity bias $\left\langle a\right\rangle$ as $\Delta G={\mathrm{ln}}[(1-\left\langle a\right\rangle )/\!\left\langle a\right\rangle ]$. Consistent with this, we found that for both Tar [QEEE] and Tsr-I214K arrays, the residence time intervals between switching events were exponentially distributed across the full range of $\left\langle a\right\rangle$ (Fig. 2c), and the average residence time of each cell $\left\langle \Delta {t}_{{\rm{up,down}}}\right\rangle$ as a function of Δ*G* was found to obey an Arrhenius-type exponential scaling $\left\langle \Delta {t}_{\mathrm{up,down}}\right\rangle (\Delta G)=\langle \Delta t\rangle {{\rm{e}}}^{-{\gamma }_{\mathrm{up,down}}\Delta G}$ (Fig. 2d), where 〈Δ*t*〉 is a characteristic residence timescale independent of the cell’s activity bias and *γ*_up,down_ are fitting constants. From the crossings of the Arrhenius-fit lines in Fig. 2d, we determined 〈Δ*t*〉 for both receptor species: 〈Δ*t*〉_Tar_ = 47.0 ± 1 s and 〈Δ*t*〉_Tsr_ = 65.5 ± 1 s.

## Switching temporal statistics collapse to those of two-dimensional Ising model

To investigate whether and how the observed temporal statistics could be explained by cooperative subunit interactions, we turned to theory. We used an Ising-type conformational spread model of allosteric cooperativity^44–47^, which assumes that subunit conformations are coupled through nearest-neighbour interactions. The strength of these interactions is parameterized by a coupling energy *J* (promoting order) whose magnitude relative to *k*_B_*T* (promoting disorder) determines a finite spatial range (that is, a correlation length) over which action at one site can affect distant sites. By varying *J*, therefore, the model can represent allosteric systems along a continuous scale of conformational disorder, including that of the more widely used Monod–Wyman–Changeux model (which is recovered on taking the fully ordered limit *J* → ∞ and has been applied extensively in modelling chemosensory arrays^48–51^). Importantly, for signalling function, two-dimensional Ising models are known to exhibit a continuous phase transition as a function of *J*, with a spontaneous ordering of subunit conformations above a critical coupling energy J*. Although so far, strong experimental support for conformational spread models has been obtained in one-dimensional protein rings^52,53^, Ising models exhibit a critical point only in two or higher dimensions, and the implications of the Ising phase transition in two-dimensional protein assemblies remained untested experimentally.

**Extended Data Fig. 5:**
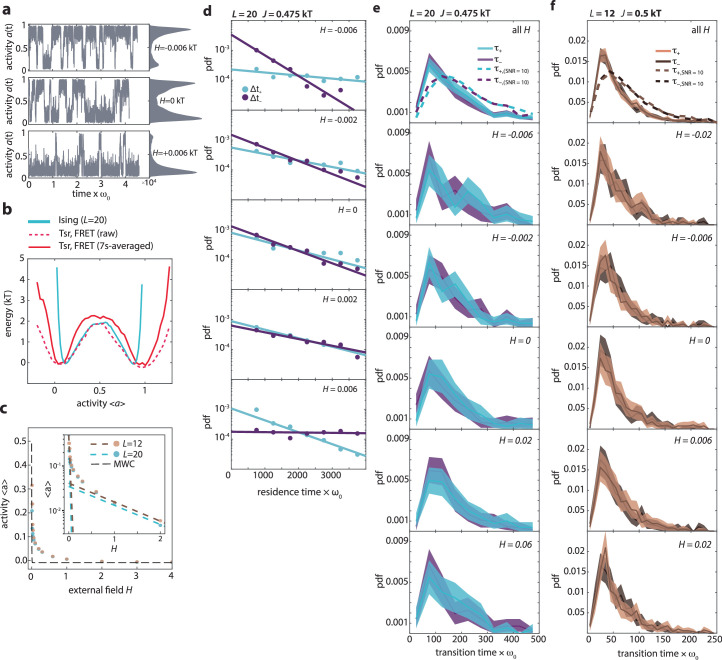
Influence of external biasing field H on conformational-spread (Ising) model. (**a**) Example time series and histograms from simulations of the conformational spread model with L = 20, coupling energy J = 0.475 *k*_B_*T* and external field as indicated. In all panels, k represents the Bolzmann constant (k_B_). (**b**) Energy landscape based on 8 simulation runs as shown in panel (**a**) with *H* = 0, calculated as -log of the histogram (cyan line). For comparison, the energy landscape based on the experimental FRET time series on Tsr-I214K is superimposed, both for the raw time series (red dashed line) as well as data processed with a 7 s moving average filter (red solid line; see Fig. 2b). (**c**) Array activity 〈*a*〉 as a function of a biasing field H for two different Ising lattices (blue: *L* = 20, *J* = 0.4875 *k*_B_*T* and brown: *L* = 12, *J* = 0.50 *k*_B_*T*). The response was determined by measuring the mean activity after applying a field H to 192 independent runs of randomly initialized lattices with a(t=0) 0.5. Also shown is the field dependence of a Monod-Wyman-Changeux (MWC) lattice of L=20, the strongly coupled limit of the conformational spread model. Inset: 〈*a*〉 on a logarithmic activity axis. The dashed lines (brown: *L* = 12, cyan: *L* = 20) indicate the activity scaling based on the slope d log(〈*a*〉)/dH around 〈*a*〉=0.5 and the expected scaling for a single lattice unit for large H: d log(〈*a*〉)/dH =-1. See Supplementary Note 2 (Section 2.7) for a detailed explanation regarding the scaling parameters. (**d**) Residence time events from Ising simulations with a lattice size L of 20 by 20 spins with a coupling energy of *J* = 0.475 *k*_B_*T*, together with exponential fits to the data (dashed lines). Each event is sorted by the bias H of the external field. See Fig. 2g for residence time distributions with a lattice size *L* of 12 by 12 spins and coupling energy of *J* = 0.5 *k*_B_*T*. (**e**) Histograms of transition time events from Ising simulations with a lattice size L of 20 by 20 spins and coupling energy of *J* = 0.475 *k*_B_*T*. Each event is sorted by the bias H of the external field. Lines and shaded areas represent the average with 95% confidence intervals obtained through bootstrap resampling. pdf: probability density function. (**f**) As in (**e**), but from Ising simulations with a lattice size L of 12 by 12 spins and a coupling energy of *J* = 0.5 *k*_B_*T*. Shaded areas represent 95% confidence intervals obtained through bootstrap resampling. As in the experiments, mean transition times are independent of the activity bias (here caused by an external field, see Supplementary Tables 9 and 11). The Ising lattice sizes and coupling energies were chosen in both cases to recapitulate the ratio of the residence over transition times determined experimentally for Tsr.

We performed kinetic Monte Carlo simulations on an *L* × *L* lattice of allosteric units with free boundary conditions, each of which can flip between two conformational states, active (*a* = 1) or inactive (*a* = 0) (Fig. 2e). The flipping rate of the unit at site *i* was modified from a fundamental flipping frequency *ω*_0_ by the influence of its nearest neighbours (*j* ∈ 1…*N*_*j*_, where *N*_*j*_ is the number of nearest neighbours) through the coupling energy *J* (in units of *k*_B_*T*) as $\omega ={\omega }_{0}\exp[{-J(2{a}_{i}-1){\sum }_{j}(2a_{j}-1)}]$, corresponding to an Ising model in which each active–inactive bond on the lattice contributes an energy penalty of *J* (Methods). At a low coupling strength (*J* ≪ J*), each unit switches independently and the total activity of the array demonstrates only small fluctuations about its mean value at $\left\langle a\right\rangle =1/2$ (Fig. 2e). However, as the coupling energy is increased towards its critical value J*, the correlation length approaches the finite size of the array, generating a double-well potential and the arrays exhibit switching events between fully active and fully inactive states (Fig. 2f and Extended Data Fig. 5). We analysed the temporal activity statistics of a simulated array with parameters within this two-state switching regime, with various values of a weak biasing field *H*_b_ that modifies the flipping rate by a factor ${{\rm{e}}}^{{H}_{{\rm{b}}}(a-1/2)}$, to approximate the diverse FRET activity biases observed across individual cells in the population (Extended Data Fig. 1e,f). Simulated residence time distributions (Fig. 2g and Extended Data Fig. 5d) were in excellent agreement with their experimental counterparts (Fig. 2c), recapitulating their characteristic exponential shape at each activity bias.

**Extended Data Fig. 6:**
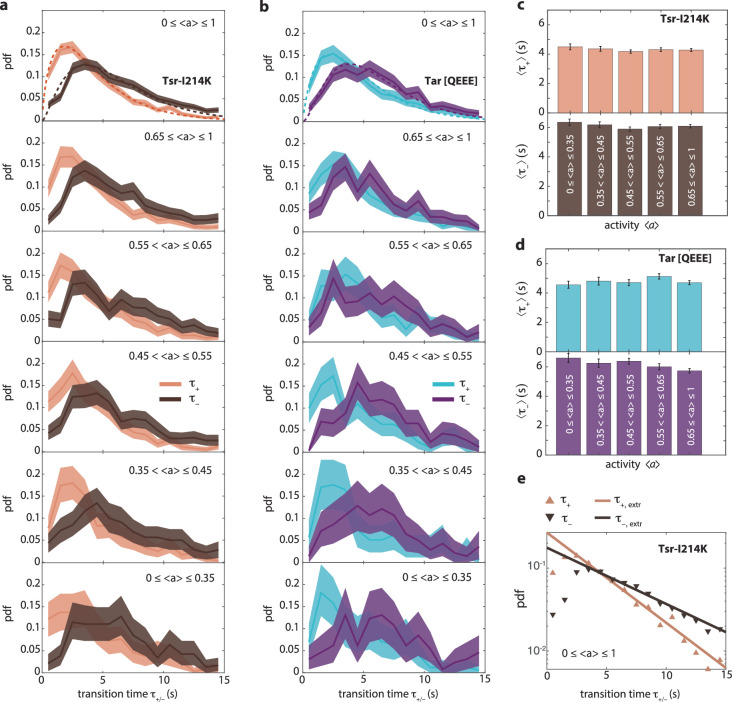
Robustness of experimental transition time extraction. (**a**) Histograms of transition time events across 549 cells expressing Tsr-I214K with each event sorted by the activity bias of the corresponding cell. Lines and shaded areas represent average with 95% confidence intervals obtained through bootstrap resampling. pdf: probability density function. Dashed lines: gamma distribution fits with the following parameters: upwards transition: *α* = 1.72 and *β* = 2.5; downwards transition: *α* = 2.45 and *β* = 2.48. Mean upwards and downwards transition times are independent of the activity bias (Supplementary Table 4). (**b**) As in (**a**), but with 204 cells expressing Tar [QEEE]. Shaded areas represent 95% confidence intervals obtained through bootstrap resampling. pdf: probability density function. Dashed lines: gamma distribution fits with the following parameters: upwards transition: *α*= 1.87 and *β*= 2.57; downwards transition: *α*= 2.74 and *β*= 2.2. Mean upwards and downwards transition times are independent of the activity bias (Supplementary Table 6). (**c**) Histograms of mean transition time per activity bias for 549 cells expressing Tsr-I214K; (top) mean upwards transition time, (bottom) mean downwards transition time. Error bars represent standard error of the mean. Mean upwards and downwards transition times are independent of the activity bias. Mean transition times and number of data points are shown in Supplementary Table 4. (**d**) As in (**c**), but for 204 cells expressing Tar [QEEE]. Mean transition times and number of data points are shown in Supplementary Table 6. (**e**) Analysis of possible undersampling bias due to finite acquisition rate (1 s^−1^). Extrapolation of the transition time distributions for cells expressing Tsr-I214K for the extreme case where the true shape of the distribution follows an exponential distribution instead of a gamma distribution. Exponential fits yield the following extrapolated transition times: *τ*_+_ = 3.72 s and *τ*_−_ = 5.67 s, which fall within 15 percent of the measured values of *τ*_+_ = 4.29 s and *τ*_−_ = 6.07 s.

By contrast, the measured transition time distributions had peaked profiles for both Tar and Tsr arrays (Fig. 2h). The average downward transition time $\left\langle {\tau }_{-}\right\rangle$ and the average upward transition time $\left\langle {\tau }_{+}\right\rangle$ were similar between Tar and Tsr arrays, with $\left\langle {\tau }_{-}\right\rangle$ slightly greater than $\left\langle {\tau }_{+}\right\rangle$ in both cases ($\left\langle {\tau }_{+}^{\,{\rm{Tsr}}}\right\rangle =4.29\pm 0.06$ s, $\left\langle {\tau }_{-}^{\,{\rm{Tsr}}}\right\rangle =6.07\pm 0.07$ s, $\left\langle {\tau }_{+}^{\,{\rm{Tar}}}\right\rangle =4.79\pm 0.08$ s, and $\left\langle {\tau }_{-}^{\,{\rm{Tar}}}\right\rangle =6.06\pm 0.09$ s; mean ± s.e.m.). These modest yet significant differences between $\left\langle {\tau }_{+}\right\rangle$ and $\left\langle {\tau }_{-}\right\rangle$ hint at the breaking of time-reversal symmetry and possible non-equilibrium driving^54,55^, and are not captured by the equilibrium Ising model. Remarkably, however, when normalized by their respective mean values to remove this asymmetry, we observed a remarkable collapse of all measured transition time distributions onto the profile of the simulated distributions (Fig. 2i). Furthermore, both measured and simulated transition-time statistics demonstrated no dependency on the activity bias (Extended Data Figs. 5e,f and 6). Collectively, the high degree of quantitative agreement between these measured and simulated temporal statistics suggest that an Ising-type conformational spread model with near-critical coupling strength (*J* ≈ J*) provides an excellent approximation to chemoreceptor array dynamics.

## Finite-size scaling analysis reveals near-critical cooperativity

**Fig. 3:**
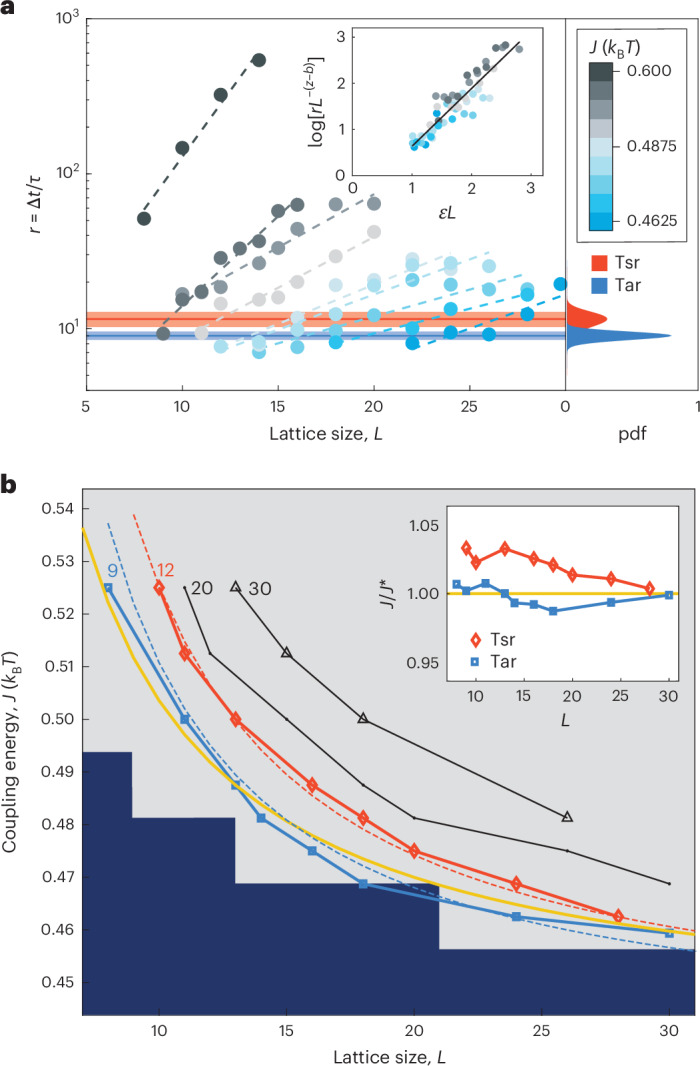
Finite-size scaling analysis reveals near-critical cooperativity of chemosensory arrays. **a**, Left: switching timescale ratio $r=\left\langle \Delta t\right\rangle/\left\langle \tau \right\rangle$ for various values of the coupling energy *J* (blue to black) as a function of lattice size *L* (circles), with exponential fits (dashed lines). Inset: data collapse for the near-critical region (*J* < 0.6*k*_B_*T*), on rescaling by factors $\epsilon =\left|\;{J}^{* }-J\right|/J$ and *L*^−(*z*−*b*)^ according to the finite-size scaling theory (Extended Data Fig. 7 shows the determination of scaling parameters). Right margin: estimated distribution (Supplementary Fig. 8) of the switching timescale ratio $\left\langle \Delta t\right\rangle /\left\langle \tau \right\rangle$ across cells, for Tsr-I214K (red) or Tar [QEEE] (blue). The shaded areas (left margin) correspond to one standard deviation of the estimated timescale ratio distribution. **b**, Phase diagram of the switching behaviour as in the *L*–*J* plane. Parameter value pairs generated polarized (grey region) and non-polarized (dark blue region) fluctuations as estimated from the peak-valley ratio of the time series histograms. Isolines of similar switching timescale ratios, as indicated, were constructed from the simulations (beaded coloured and black curves). Theoretical finite-size scaling predictions for the critical coupling energy J*(*L*) (ref. ^62^; gold curve) and the switching time ratios (dashed lines; Supplementary Equation (9) and Supplementary Table 16 list the fit parameters) are superimposed. Inset: switching timescale ratio isolines for Tar (blue) and Tsr (red), normalized by the finite-size scaling of the critical coupling energy J* reveals values within 3% of the critical energy.

**Extended Data Fig. 7:**
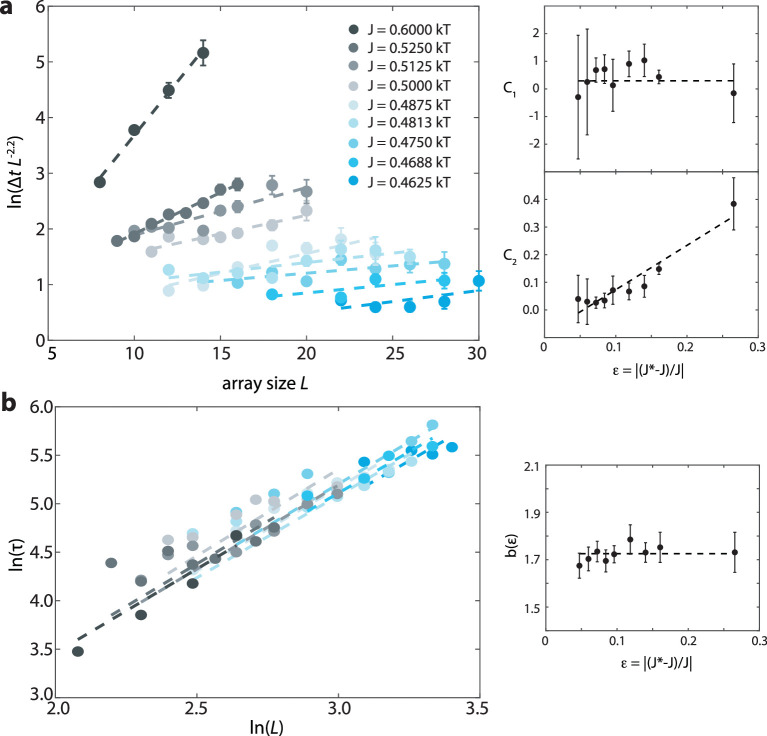
Mean residence and transition times as a function of lattice size obtained from Ising simulations. (**a**) (Left) Mean residence times as a function of array size L, scaled according to equation S4 (Supplementary Note 2). Dashed lines are fits of a function of the form c_1_ + c_2_*L* to the data and colours indicate different coupling energies (the same colour scheme is used throughout the figure). (Right) Scaling of the fitting parameters c_1_ and c_2_ as a function of the energy level *ε*. (**b**) (Left) Logarithm of mean transition times as a function of the logarithm of the array size L. Dashed lines are fits of the linear form ln(*τ*) = *b*(*ε*)ln(*L*); (right) Fitted values of *b*(*ε*) demonstrated nearly no dependence on *ε*, and hence in our scaling analysis we treated the exponent *b* as fixed parameter that is independent of *ε*. Error bars throughout the figure represent standard error of the mean. Residence and transition times were extracted from switching events detected in at least six independent simulations for each combination of lattice size and coupling energy. Determined scaling constants are listed in Supplementary Table 16.

We sought to quantify the degree to which both Tar and Tsr arrays are close to criticality. Crucial in considering critical phenomena in living systems are finite-size effects^56^, which modify the quantitative behaviour near critical points compared with well-known results derived in the thermodynamic limit (where the system size *L* → ∞). Finite-size scaling theory of Ising-type models is highly developed^57,58^, but direct comparisons between the temporal statistics of the Ising model and our experimental data are complicated by the fact that the fundamental flipping timescale 1/*ω*_0_ of cooperative units (corresponding to the spin-flip timescale in the Ising model) remains unknown. We, therefore, identified—as a key experimental observable—the dimensionless ratio $r\equiv \left\langle \Delta t\right\rangle /\left\langle \tau \right\rangle$ between the residence and transition timescales (with $\left\langle \tau \right\rangle$ defined as the average $\left\langle \tau \right\rangle \equiv (\left\langle {\tau }_{+}\right\rangle +\left\langle {\tau }_{-}\right\rangle )/2$; Supplementary Note 2), which divides out the *ω*_0_-dependence to enable direct comparisons. Simulations at various values of *J* indeed revealed a strong dependence of this ratio *r* on *L* (Fig. 3a), with all results collapsing onto a single curve defined by the finite-size scaling relation $r\approx {L}^{(z-b)}\exp ({c}_{0}\epsilon L)$, where *z*, *b* and *c*_0_ are scaling constants and $\epsilon =\left|\;{J}^{* }-J\right |/J$ is the ‘reduced temperature’ providing a dimensionless measure of the (energetic) distance to criticality^59,60^ (Supplementary Note 2.5 and Extended Data Fig. 7 detail the determination of the scaling constants).

Because different combinations of *J* and *L* can yield the same value of *ϵL*, this scaling does not allow the unique determination of *J* from the measured values of *r* (Fig. 3a). However, finite-size scaling theory predicts that the critical coupling energy J*, separating the highly ordered (polarized) and disordered (non-polarized) regimes, also depends on the system size *L* (refs. ^61,62^). Consistent with this prediction, a phase diagram on the *J*–*L* plane constructed from simulations showed that the boundary between polarized (Fig. 3b, grey region) and non-polarized (Fig. 3b, blue region) dynamics coincided well with the theoretically predicted scaling (Fig. 3b, gold curve) J*(*L*) ≈ J*(∞)/(1 − *cL*^−1^), where J*(∞) ≈ 0.44*k*_B_*T* is the critical coupling energy in the thermodynamic limit (*L* → ∞) and *c* is a boundary-condition-dependent constant (*c* = 1.25 for free boundary conditions^62^; Supplementary Note 2.3). Interestingly, we found that isolines ${J}_{r}^{\,{\rm{iso}}}(L)$ corresponding to *L*–*J* combinations that yield the same timescale ratio *r* (Fig. 3b, beaded curves) were nearly parallel with the profile of J*(*L*). Dividing J*(*L*) from the isolines ${J}_{r}^{\,{\rm{iso}}}(L)$ corresponding to the measured *r* values for Tar (*r*_Tar_ ≈ 9; Fig. 3b, blue) and Tsr (*r*_Tsr_ ≈ 12; Fig. 3b, red) revealed a remarkable confinement of the coupling energy for both Tar and Tsr to within ±3% of J*(*L*) across a broad range in *L* (Fig. 3b, inset). Thus, despite uncertainty in the array size *L*, finite-size scaling analysis of the observed temporal statistics strongly suggests that both Tar and Tsr arrays are poised very close to the Ising critical point.

## Arrays without adaptation feedback exhibit critical slowing of response

**Fig. 4:**
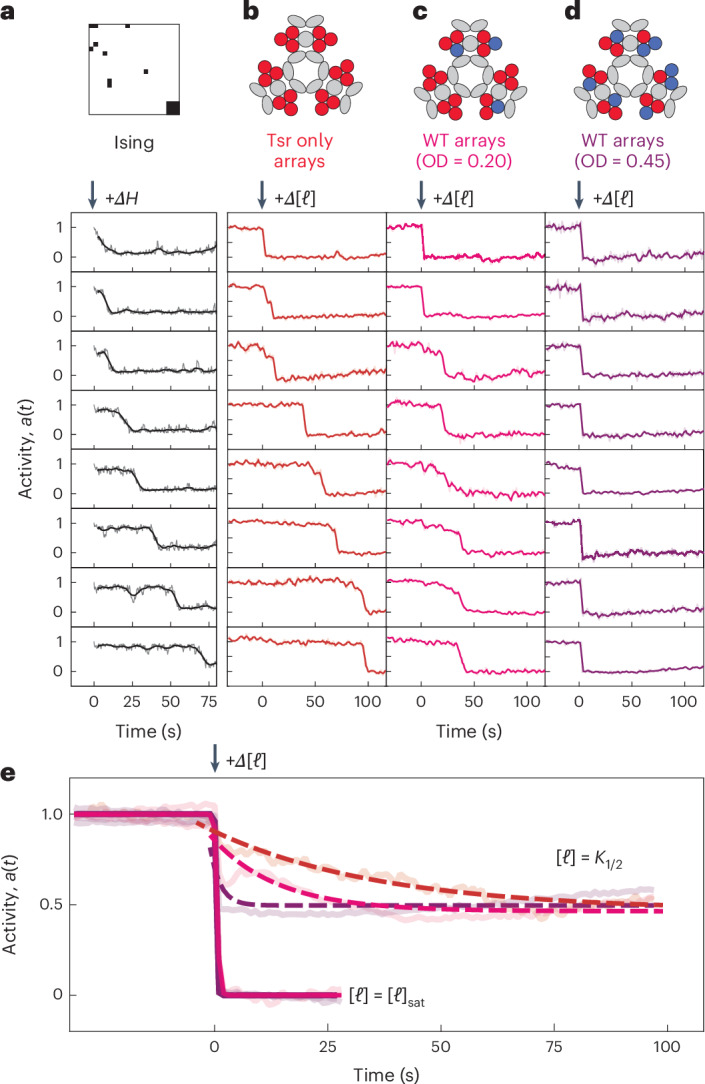
Experimental demonstration of critical slowing down in chemosensory arrays. **a**, Example activity time series from stochastic simulations (‘Ising’) of a single array (*L* = 20, *J* = 0.48*k*_B_*T*), initialized as all-active ($\left\langle a\right\rangle \approx 1$) state at time *t* = 0 (arrow), when a positive external field (Δ*H* = 0.02*k*_B_*T*, mimicking sub-saturating chemoattractant addition) was applied. Raw trajectories (light grey) are overlaid with 7-s low-pass-filtered data (dark grey). **b**, Representative activity time series from single-cell FRET measurements in non-adapting cells expressing only the WT Tsr receptor (strain TSS1964/pPA114), responding to a sub-saturating 20 μM l-serine stimulus (*Δ*[*ℓ*]) applied at *t* = 0 (arrow). Top: schematic of the top view of the chemosensory array, reflecting different compositions (red circles represent Tsr receptors; blue circles, Tar). Raw FRET time series (light red) are overlaid with 3-s low-pass-filtered data (dark red). **c**, Same data as **b**, but for non-adapting cells expressing WT receptor complement (TSS1845), harvested at OD = 0.20 to yield arrays with a low degree of receptor-species mixing. Cells were stimulated with 35 μM l-serine. **d**, Same data as **c**, but cells harvested at OD = 0.45, leading to a high degree of receptor-species mixing. Cells were stimulated with 50 μM l-serine. **e**, Response timescale to l-serine derived from population-averaged experimental time series of Tsr-only arrays (red, 100 cells), WT OD = 0.20 (low mixing) arrays (pink, 119 cells) and WT OD = 0.45 (high mixing) arrays (magenta, 208 cells). Response times for sub-saturating stimuli (from exponential fits) and saturating stimuli (from sigmoidal fits) were 37.19 s and 0.33 s (Tsr only, 20 μM and 1 mM); 14.5 s and 0.71 s (WT OD = 0.20, 35 μM and 1 mM); 2.24 s and 0.31 s (WT OD = 0.45, 50 μM and 1 mM). All experiments were performed in a microfluidic device enabling rapid ligand exchange (~100 ms)^91^ on non-adapting cells, and chemoeffector stimulus (*Δ*[*ℓ*]) delivery was temporally aligned to *t* = 0 using a fluorescent dye pulse preceding l-serine addition for precise timing. Arrays were fully active (*a*_0_ ≈ 1) before stimulation (Supplementary Fig. 3).

**Extended Data Fig. 8:**
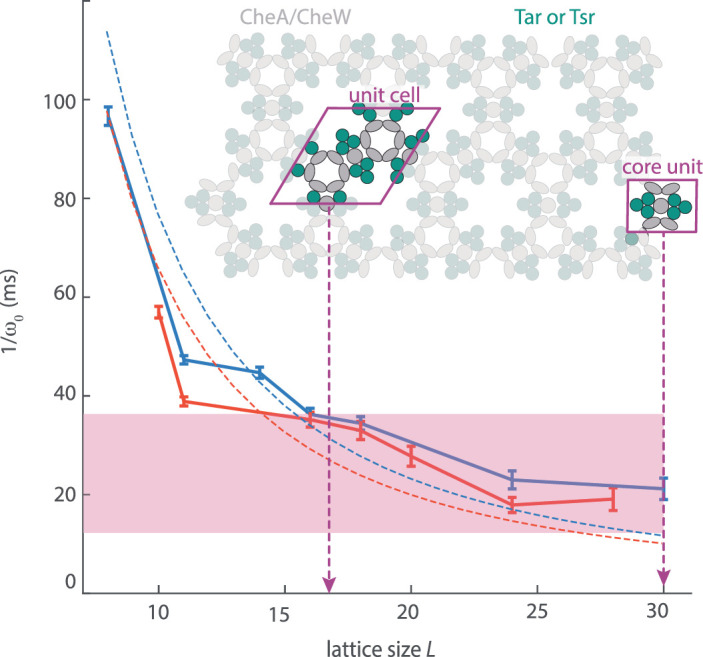
Estimation of the intrinsic fluctuation timescale of allosteric units. Fundamental transition timescale 1/*ω*_0_ of allosteric units, plotted as a function of the assumed array size *L* for Tar (blue) or Tsr (red) arrays, based on simulations calibrated by experimentally observed residence- and transition-times (solid lines). Shown together are the expected dependence based on finite-sized scaling theory (blue and red dashed lines; see Supplementary Note 2.6). Two possible fundamental units of cooperativity in the bacterial chemosensory array are highlighted within a schematic top view of chemosensory arrays: ‘core unit’ consisting of a CheA kinase dimer, two CheW monomers, and two trimers of chemoreceptor dimers (purple rectangle) and the repeating unit cell of the array, consisting of one full core unit and four half core units (purple parallelogram). Each chemosensory array contains approximately 1000 core units or 300 repeating unit cells, leading to lower and upper limits of array size (purple dashed lines). Using these as lower and upper limits yields a timescale range 1/*ω*_0_ ≈ 15 − 35 ms (shaded area). Error bars refer to s.e.m.

Finite-size scaling analysis also allowed us to estimate the fundamental flipping timescale 1/*ω*_0_ of allosteric units. Combining the observed residence and transition times with an approximate range for the size *L* based on the known structure, stoichiometry and component expression levels of the array, the scaling could be calibrated to yield 1/*ω*_0_ ≈ 10–35 ms (Extended Data Fig. 8 and Supplementary Note 3). This allowed us to directly compare the response dynamics between simulations and experiments. Previous theoretical studies have shown that signal amplification due to coupling within Ising-type conformational spread models comes at the cost of prolonged response times^47,63^, potentially impairing temporal comparisons during run-and-tumble navigation^64–66^. To address the influence of near-critical cooperativity on response times to external stimuli, we first simulated the dynamic response of arrays with *J* ≈ J* after an applied step stimulus (implemented within the model by an external ligand field Δ*H* that modifies the flipping rate *ω*_0_ by a factor of e^Δ^^*H*(2*a*−1)/2^; Methods) to mimic kinase inactivation on attractant chemoeffector stimulation. These simulations demonstrated that near-critical Ising arrays indeed respond slowly, with a broad distribution of times averaging tens of seconds (Fig. 4a). We then conducted FRET experiments with a sub-saturating stimulation of adaptation-deficient cells expressing homogeneous arrays (consisting of a single chemoreceptor species, Tsr) using a polydimethylsiloxane (PDMS) microfluidic flow cell that allowed for chemoeffector concentration changes much faster than the acquisition frequency of 1 Hz (Methods). The experimental response time courses (Fig. 4b) were remarkably similar to those of the array simulations (Fig. 4a), thereby confirming that the drastic slowing of response can indeed occur in *E. coli* homogeneous chemosensory arrays near criticality.

Chemosensory arrays in WT *E coli* cells are known to mix multiple chemoreceptor species^67^, and at the population level, such receptor mixing has been shown to attenuate (though not eliminate) ligand–response cooperativity^48^. Considering our current finding that the strength of nearest-neighbour interactions within homogeneous arrays are near critical (*J* ≈ J*) for both Tar and Tsr arrays, attenuated cooperativity upon mixing Tar and Tsr suggests that *J* might take on a different value between lattice sites occupied by homologous and heterologous receptors (that is, *J*_Tar–Tar_ ≈ *J*_Tsr–Tsr_ ≈ J*, but *J*_Tar–Tsr_ ≠ J*). Thus, receptor-species mixing introduces a spatial disorder in coupling (that is, a ‘bond disorder’) that potentially limits the extent of cooperative interactions and, therefore, attenuates critical slowing down (Supplementary Note 4). Experimentally, the degree of chemoreceptor mixing can be varied by controlling cell culturing conditions. Cells with a WT chemoreceptor complement are known to have a relatively low degree of mixing when harvested at an early exponential phase (array dominated by Tsr), but a higher degree of mixing when harvested at mid-exponential phase (increased Tar)^68,69^ (Supplementary Note 5). FRET experiments on adaptation-deficient cells harvested at these different growth phases revealed that although receptor mixing can indeed attenuate response slowdown (Fig. 4c,d,e), responses to sub-saturating stimuli at early exponential phase can still be very slow (>10 s), limiting the speed at which bacteria can respond. Taken together, these results provide a clear experimental demonstration that cooperative interactions within the array affect the signalling response timescale of *E. coli*. Although cooperativity-induced response slowdown is most acute in cells engineered to express only a single chemoreceptor species, it is substantial also in cells with the WT complement of chemoreceptor species.

## Near-critical cooperativity balances a speed–amplitude trade-off

**Fig. 5:**
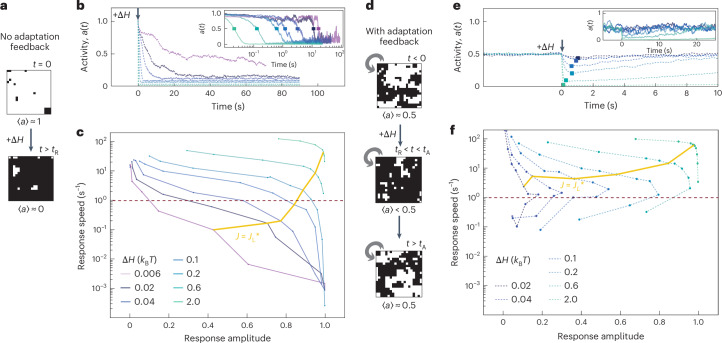
Near-critical cooperativity balances response amplitude and speed. **a**, Top: without adaptation feedback, near-critical cooperativity extends the range of cooperativity across the entire array, leading to all-or-none response. Shown are example states (binary squares) of an array initialized in the all-active ($\left\langle a\right\rangle \approx 1$) state at time *t* = 0 when a positive external field (Δ*H* > 0, mimicking chemoattractant addition) was applied, and at a later time *t* > *t*_R_ exceeding the response time *t*_R_ of the array. **b**, Simulated response time series (dashed curves) of activity bias $\left\langle a\right\rangle (t)$, computed by averaging 48 stochastic trajectories of a 20 × 20 array without adaptation feedback and *J* = 0.47*k*_B_*T*, for various values of added external field Δ*H* (**c** shows the colour code), initialized at 〈*a*〉 ≈ 1 and assuming 1/*ω*_0_ = 30 ms (Extended Data Fig. 8). Inset: example single-array stochastic trajectories (solid curves), which invariably demonstrate all-or-none switching-like response. The coloured rectangles illustrate the definition of the response time *t*_R_ as the average time of crossing half-maximal activity, *a* = 0.5 (coloured rectangle). The response amplitude is defined as $1-\left\langle a\right\rangle (J,\Delta H)/0.5$. **c**, Speed–amplitude trade-off in array responses without adaptation feedback. For each stimulus size Δ*H* (see the legend for colour code), a thin curve indicates the dependence of response amplitude and speed on the coupling energy *J*. The thick gold curve is the critical isoline, tracing out points on each thin curve corresponding to $J={J}_{J}^{* }$. The horizontal dashed line represents the speed required to respond within a typical run time (1 s) of *E. coli*^92^, corresponding to an approximate lower bound for effective run-and-tumble chemotaxis. **d**, Adaptation feedback injects spatial disorder within the array that limits the extent of cooperativity and stabilizes intermediate-activity states. Shown are example array states (binary squares) in the presence of adaptation feedback (indicated by grey arrows), before stimulus (*t* < 0), just after response (*t*_R_ < *t* < *t*_A_) and following sensory adaptation (*t* > *t*_A_), each with corresponding activity bias $\left\langle a\right\rangle$. **e**, Simulated response time series (dashed curves) of activity bias 〈*a*〉(*t*), computed by averaging 96 stochastic trajectories of a 20 × 20 array with adaptation feedback and *J* = 0.47*k*_B_*T*, for various values of added external field Δ*H* (**f** shows the colour code), initialized at 〈*a*〉 ≈ 0.5. Response time and amplitude are determined from an exponential fit to the averaged activity time series (coloured rectangles). Inset: example single-array stochastic trajectories (solid curves), smoothed with a 7-s moving-average filter to aid visual inspection. **f**, Same data as **c**, but for speed–amplitude trade-off in array responses with adaptation feedback. Note that response slowdown is mitigated compared with non-adapting arrays, and the speed of near-critical arrays (thick gold curve) remains above the lower-bound response speed set by the run-and-tumble behaviour timescale (horizontal dashed line) across all the stimulus sizes.

Why are bacterial chemosensory arrays poised so close to criticality, despite potentially deleterious slowing of response? To address this question, we examined the relationship between response speed and response amplitude using simulations for various combinations of *J* and Δ*H* (Fig. 5a,b). For every stimulus size Δ*H*, increasing the coupling strength *J* led to a decrease in the response speed (defined as the inverse of response time) but an increase in response amplitude, indicating a trade-off (Fig. 5c). The profile of these *H*_L_ isolines are interesting when viewed as a Pareto front^70^ for navigating the trade-off, having a convex shape with a knee above which the response speed drops off sharply. Remarkably, the critical coupling energy (*J* = J*; Fig. 5c, gold curve) traverses this knee at every stimulus size, indicating that near-critical cooperativity enables a balancing of these two response objectives, allowing for large response amplitudes without drastically compromising the response speed.

**Extended Data Fig. 9:**
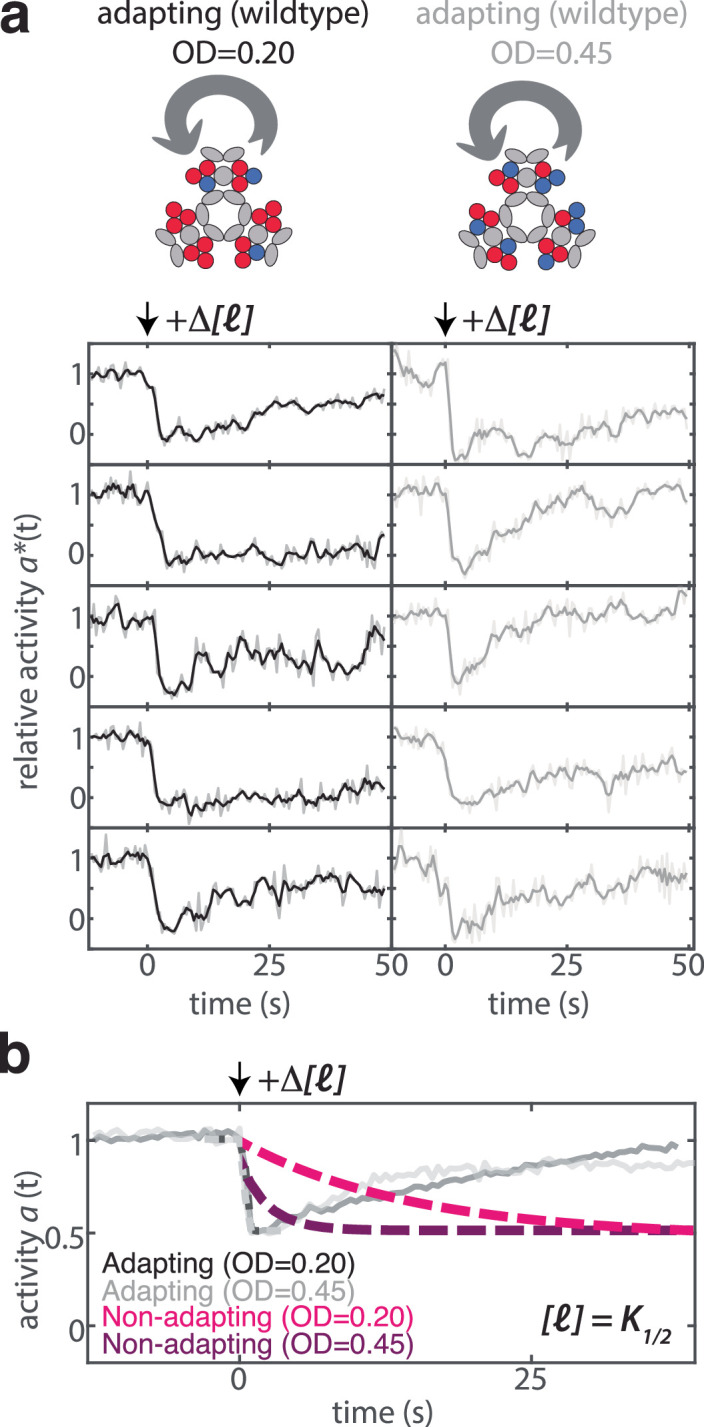
Wildtype (adapting) cells reveal fast response to ligand addition. (**a**) Representative relative activity a* obtained from FRET time series for single cells (strain TSS1846) harvested in early exponential phase (OD=0.20; left, black) and mid-exponential phase (OD=0.45; right, light grey) responding to a concentration [*ℓ*] of 0.05 *μ*M l-Serine. The illustrations on top indicate the level of chemoreceptor mixing (low and high levels of chemoreceptor mixing for OD 0.20 and 0.45 respectively) and the arrow represents the presence of adaptation feedback. Experiments were performed in a microfludic device with fast exchange rate (≈ 100 ms, ref. ^91^) and an acquisition frequency of 2 Hz. Stimulus delivery was temporally aligned to t=0 using a fluorescent dye pulse preceding l-serine addition for accurate timing. Due to limited photon budget and constraints in device architecture, no saturating attractant or repellent stimulus was applied, and the response was normalized to the average FRET level before stimulus. The steady-state activity of wildtype cells is *a*_0_ ≈ 0.3 for both ODs (Extended Data Fig. 10g) (**b**) Population averaged response of 384 wildtype (adapting) cells harvested at OD=0.20 (dark grey) and 98 cells harvested at OD=0.45 (dark gray) to a subsaturating stimulus (same experiment as panel **a**). The relative activity time series is shown together with sigmoidal fits to the activity decay (grey and black dashed lines), as well as the fits to the activity decay to a subsaturating stimulus in non-adapting cells (Fig. 4, coloured dashed lines). To facilitate comparison, the activity of wildtype cells was rescaled and set between 1 and 0.5. The fit values were 0.22 s for OD=0.20 and 0.05 s for OD=0.45. These timescales are close to the response time obtained in classic tethering experiments, but are likely limited in resolution because of the exchange time of the microfluidic device (100 ms).

**Extended Data Fig. 10:**
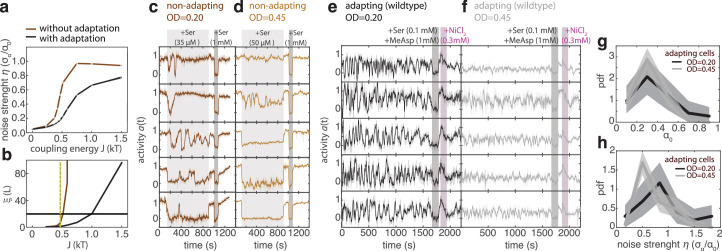
Enhancement and attenuation of steady-state noise in wildtype arrays. (**a**) Noise strength *η* in the Ising model as a function of coupling energy *J*, measured as the standard deviation in array activity divided by the mean array activity, averaged across simulation runs with *L* = 20. Results are shown for lattices with (192 runs) and without adaptation (48 runs). For all values of *J*, adaptation feedback decreases the noise strength. (**b**) Correlation length *ξ* as a function of coupling energy *J*, computed as the average value of *ξ* across 48 independent array states for each value of *J*, with (black) and without (brown) adaptation feedback. The correlation length ξ increases with *J* (panel **a**), increasing the noise strength. However, as the correlation length *ξ* of the lattice size increases, activity fluctuations become fully binary, resulting in the saturation of the noise strength *η* at a noise plateau. (**c**) Representative activity time series of adaptation-deficient cells expressing all 5 chemoreceptor species (TSS58), harvested at low optical density (OD = 0.20) where their receptor clusters are primarily comprised of Tsr (light brown) superimposed with a low-pass filtered time series with a time window of 5 seconds (dark brown). Cells are stimulated with a sub-saturating l-Serine stimulus of 35 *μ*M and a saturating stimulus of 1 mM. The saturating stimulus defines the minimum activity (0), and the buffer condition corresponds to maximum activity (1) in these cells (see Supplementary Figure 3). (**d**) Same as (**c**), but for cells harvested at an intermediate optical density (OD = 0.45), where their receptor clusters are comprised by equal amounts of Tar and Tsr. Cells are stimulated with a sub-saturating l-Serine stimulus of 50 *μ*M and a saturating stimulus of 1 mM. (**e**) Representative activity time series of adapting cells expressing all 5 chemoreceptor species (TSS1846), harvested at low optical density (OD = 0.20), where their receptor clusters are primarily comprised of Tsr. Cells are exposed to a saturating attractant mixture (0.1 mM l-Serine and 1 mM MeAsp) and repellent (0.3 mM NiCl_2_) to normalize kinase activity between 0 and 1. (**f**) Same as (**e**), but for cells harvested at an intermediate optical density (OD = 0.45) where their receptor clusters are comprised by equal amounts of Tar and Tsr. (**g**) Histograms of steady-state activity *a*_0_ for 39 adapting cells harvested at OD = 0.20 (black) and 92 adapting cells harvested at OD = 0.45 (gray). Lines and shaded areas indicate mean with 95% confidence intervals from bootstrap resampling. (**h**) Histograms of noise strength for the same cells as in (g), calculated as the standard deviation of a 10-second low-pass filtered time series, normalized by its mean FRET level (representing steady-state activity level *a*_0_). Lines and shaded areas indicate mean with 95% confidence intervals from bootstrap resampling.

However, these Ising simulations also indicate that at *J* = J*, the speed of response to small-amplitude stimuli (*H* < 0.2) can fall below the typical run-and-tumble frequency of swimming *E. coli* (~1 s; Fig. 5c, dashed line), whereas WT *E. coli* cells are known to respond on a much faster timescale (~100 ms; refs. ^71,72^ and Extended Data Fig. 9). A salient difference between the Ising simulations and WT *E. coli* is that the latter is regulated by an adaptation system that maintains a constant steady-state output through the reversible methylation of receptors. These activity-biasing covalent modifications are added and removed at random sites by the adaptation enzymes CheR and CheB throughout the array^73^ and, therefore, amount to another source of spatial disorder (a ‘field disorder’) that can further limit the extent (correlation length) of cooperative interactions within the array^45,74^ (Supplementary Note 4). To test the impact of this feedback-induced dynamic disorder on array response dynamics, we modified our simulations to include adaptation feedback (Fig. 5d,e), implemented as a stochastic modulation of a local biasing field *H*_b_ on each allosteric unit that counteracts the activity of that unit (Methods). These simulations with feedback-induced disorder also demonstrate a speed–amplitude trade-off (Fig. 5f), but the response slowdown at high values of *J* leads to a decrease in response amplitude due to the loss of timescale separation between the initial response and subsequent adaptation. Importantly, the disorder-induced reduction in the extent of cooperativity (Extended Data Fig. 10a) strongly attenuates response slowdown and near-critical (*J* ≈ J*) coupling (Fig. 5f, gold curve) achieves response speeds faster than the typical tumble frequency (Fig. 5c,f, dashed lines) for all stimulus magnitudes and maintains substantial signal amplification.

## Disorder-induced tempering of near-critical dynamics

Taken together, the above results suggest that in WT cells, the combined effects of static disorder (due to receptor-species mixing) and dynamic disorder (due to sensory adaptation) serve to temper the near-critical dynamics in a manner that eliminates the deleterious critical slowing of response and retains a substantial amplification of response. Although both kinds of disorder contribute to this tempering by effectively shifting the proximity to criticality (Supplementary Note 4), it is interesting to note that the static (bond) disorder due to receptor mixing is expected to vary with the Tar/Tsr expression level ratio—a variable that is known to undergo changes during culture growth^68,69^. This raises the intriguing possibility that proximity to criticality can be modulated physiologically through the Tar/Tsr ratio. Consistent with this idea, we found that cell populations in physiological states with a higher degree of Tar–Tsr mixing (and hence arrays with increased bond disorder) exhibited attenuated temporal fluctuations, as expected for disorder-induced tempering (Extended Data Fig. 10 and Supplementary Note 5).

Numerous experimental and theoretical studies have established that swimming *E. coli* cells can exploit such signal noise to enhance the exploratory propensity of their run-and-tumble motility^38,75–78^. Our FRET measurements of temporal fluctuations (Extended Data Fig. 10) revealed very high noise strengths (up to *η* ≡ *σ*_a_/*a*_0_ ≈ 0.88) in WT cells, exceeding previous theoretical predictions of models that did not account for the near-critical cooperativity of arrays^39,75,79,80^ by approximately three to fourfold. Hence, in addition to balancing the speed–amplitude trade-off in the sensory response, near-critical cooperativity directly impacts the exploratory propensity of swimming cells by augmenting steady-state signal noise, which can, in turn, be modulated physiologically via the Tar/Tsr expression-level ratio (Supplementary Note 5).

## Discussion

An increasing number of biological systems involving many components, from protein sequences^81^ to cellular membranes^82^, as well as communication between cells^83,84^ and even whole organisms^85,86^, have been reported to self-organize into narrow zones of phase space close to a critical point, on the boundary between order and disorder^87^. Our finding that bacterial chemoreceptor arrays are poised close to the Ising critical point reveals near-critical cooperativity as a design principle for balancing competing response objectives in protein signalling assemblies, and further experiments and analyses revealed how disorder can be harnessed to mitigate deleterious critical slowing. Recent theoretical studies^88–90^ indicate that the switching statistics observed in our experiments point to near-critical cooperativity also when the Ising framework is generalized to include non-equilibrium effects (Supplementary Note 6). Given the many spatially extended arrays being discovered across cell biology^5,6,21,22^ as well as their anticipated role in synthetic biology^12,13^, we envisage that our approach of combining *in singulo* fluctuation measurements with finite-size scaling analysis to understand their function could find use across a wide range of systems.

## Methods

### Bacterial strains and molecular biology

Spontaneous switching experiments with receptor mutants were performed using TSS1964 (ref. ^34^), a receptorless (ΔTar ΔTsr ΔTap ΔTrg ΔAer), non-adapting (ΔCheRB) derivative of *E. coli* K-12 RP437 (also known as HCB33), with the native response regulator and phosphatase deleted (ΔCheYZ). The strain expresses chromosomally encoded adhesive FliC flagellar elements, enabling immobilization on glass surfaces. Experiments on cells with WT chemoreceptor arrays were performed in either non-adapting (ΔCheRBYZ) or adapting (ΔCheYZ) strains, with adhesive FliC flagellar elements expressed from plasmid pZR1 in ΔFliC background (TSS58, as done in ref. ^34^) or by expressing adhesive FliC from the chromosome (ΔCheRBYZ, TSS1845; ΔCheYZ, TSS1846). Strains TSS1845 and TSS1846 were engineered using the Datsenko–Wanner method^93^, essentially in the same way as TSS1964 (ref. ^34^), using primers GGAATTACCCTTTCTACGGAAGC (forward) and CACATCCGCAGCGTAAAG (reverse) on strains VS149 and VS104 (ref. ^28^), respectively. In strains with chromosomally encoded adhesive FliC, an empty plasmid (pBAD33) carrying the same chloramphenicol-resistance cassette as pZR1 was introduced to ensure uniform antibiotic resistance requirements across experiments. All strains are listed in Supplementary Table 1.

Protein fusions for FRET to CheY/CheZ as well as chemoreceptor mutants were expressed from inducible plasmids. Receptor mutants deficient in ligand binding were created by introducing point mutations at chemoreceptor residue 69 by overlap extension PCR, using restriction sites EagI/BamHI on either pLC113 with forward primer CCTGAGTcaTTCAGCGGTACgg and reverse primer cGTACCGCTGAAtgACTCAGG (for Tar) or with forward primer CACCCTCAACgagGCGGGTATCCGC and reverse primer GCGGATACCCGCctcGTTGAGGGTG on pPA114 (for Tsr). Plasmids were introduced in host strains by heat shock or electrophoretic transformation. All plasmids are listed in Supplementary Table 2.

### Bacterial cell culture and media conditions

For all experiments, cells were grown in Tryptone Broth (10 g l^−1^ of bacto-tryptone and 5 g l^−1^ of NaCl; pH 7.0) at 33.5 °C with shaking, diluted 1:50 from a saturated Tryptone Broth overnight culture, with 100 μg ml^−1^ of ampicillin and 34 μg ml^−1^ of chloramphenicol in both cultures and appropriate inducers in the day culture. The FRET pair CheZ-YFP and CheY-mRFP1 was expressed (in tandem) from pSJAB106 (ref. ^34^) using 50 μM IPTG for Tar [QEEE], Tar-F396Y, Tsr-WT and 100 μM IPTG for Tsr-I214K. For experiments in which CheZ localization was used to visualize chemoreceptor clusters, IPTG induction was lowered to 15 μM for Tsr-I214K. Unless otherwise specified, cells were harvested at an optical density (OD) of 0.46–0.47, then washed once with and thereafter placed in motility media (MotM; 10 mM KPO_4_, 0.1 mM EDTA, 1 μM l-methionine, 10 mM lactic acid; pH 7.0). Because of the auxotrophic limitation of *E. coli* RP437, growth and protein expression are arrested in MotM^94^. Cells were incubated at room temperature for 1.5 h, before imaging, to allow for fluorescent protein maturation.

### FRET microscopy

Single-cell FRET microscopy was performed as reported previously^34^. For each experiment, cells immobilized on a glass coverslip were placed in a flow cell under continuous flow (400 μl min^−1^) established by a syringe pump (Harvard Apparatus PHD 2000). The sample was illuminated every second (1 Hz) with a light-emitting diode system (CoolLED pE-2) through a ×40 1.30-numerical-aperture oil objective (Nikon) for 17 ms using an upright microscope (Nikon FN1). Epifluorescent light was sent through an optical splitting device (Cairn Research OptoSplit II) in combination with a 580-nm dichroic mirror (Semrock) and two emission band-pass filters (527/42 nm and 641/75 nm; Semrock) to project the emitted donor and acceptor channels in parallel on the sensor of an electron-multiplying charge-coupled device camera (Princeton Instruments proEM 512), set with a multiplication gain of 100.

The flow-cell temperature was controlled through a custom-built stage heater, based on a water-cooled thermoelectric Peltier element controlled through a proportional–integral–derivative controller (*T* = 18 °C for Tsr-I214K, *T* = 25 °C for Tar [QEEE]). The activity bias of chemoreceptors depends on temperature^95^; therefore, we controlled the temperature to keep the average activity bias close to 1/2, at which we found the number of switching cells to be the maximum (Supplementary Fig. 1). The buffer solution temperature was controlled through a heat bath with heating and cooling capacity (Grant Instruments LT ecocool 100). Experiments involving WT Tsr ([QEQEE] and [QEEEEE]; Supplementary Fig. 2), Tsr-F396Y or mixed receptor clusters were performed at room temperature without temperature control.

In experiments where the receptor clusters were imaged in conjunction with FRET microscopy, an inverted microscope (Nikon Eclipse Ti-E), equipped with a ×100 1.45-numerical-aperture oil objective (Nikon), was utilized. For FRET microscopy, the sample was illuminated every 2 s (0.5 Hz) with a light-emitting diode system (CoolLED pE-4000) for 20 ms. Epifluorescent light was directed into a two-camera image splitter (Cairn Research TwinCam) equipped with a 580-nm dichroic mirror (Semrock) and two emission filters (520 nm and 593 nm; Semrock), leading to two identical scientific complementary metal–oxide–semiconductor cameras (Hamamatsu ORCA-Flash4.0 V2), where the emitted donor and acceptor channels were projected separately. To enhance their signal-to-noise ratio, FRET images were binned by a factor of 4 × 4 pixels. To visualize large chemoreceptor clusters resulting from CheZ-YFP localization near the chemoreceptors, the sample was imaged once at the beginning and end of each experiment for 200 ms using the same optical path as in FRET microscopy. CheZ-YFP images were not binned to preserve all the spatial information. Receptor cluster imaging experiments were performed at room temperature.

In experiments determining the response time of cells to externally applied ligand stimuli (Fig. 4 and Extended Data Fig. 9), we performed the experiment in a PDMS microfluidic flow cell that allowed for rapid (~100 ms) ligand exchange, substantially faster than in the flow cell used in the rest of our FRET experiments^96^ (~4 s at 400 μl min^−1^). The PDMS device was fabricated and operated as described previously^91^. To determine the precise delay of ligand arrival and, therefore, calculate the exact time of stimulus addition, cells were exposed to a brief pulse of the fluorescent tracer fluorescein before stimulus addition. In the experiments with the PDMS microfluidic flow cell, no chemorepellent stimuli were applied, since the cells are transiently exposed to all stimuli during cell loading, and prior exposure to repellents can alter the attractant responses, possibly due to an irreversible binding of chemorepellents, and the activity bias was determined separately (Supplementary Fig. 3). The sample was illuminated every 1 s (1 Hz) for experiments with non-adapting cells (Fig. 5) and every 0.5 second (2 Hz) for experiments with adapting cells (Extended Data Fig. 9). All response–time experiments were performed at room temperature.

### FRET analysis

Analysis of the fluorescent time series was performed essentially as reported previously^34^. After drift correction using rigid stack registration^97^ in ImageJ (v. 1.49 or higher), the images were segmented using the Mahotas Python library 1.1 (or higher)^34,98^ to obtain single-cell donor (*D*(*t*)) and acceptor (*A*(*t*)) fluorescence intensity time series. Afterwards, the intensity time series were corrected for bleaching by dividing an exponential decay. The FRET time series were calculated from the ratio of acceptor and donor emissions *R*(*t*) = *A*(*t*)/*D*(*t*) as^28,34^

1 $$\,{\rm{FRET}}\,(t)=\frac{R(t)-{R}_{0}}{R(t)+\alpha },$$

where *R*_0_ is the ratio during a large and saturating attractant stimulus (for example, the ratio in the absence of any FRET^28^) and *α* = 0.3 is a constant depending on the FRET measurement setup and the specific FRET donor–acceptor pair used^34^.

This intermolecular FRET signal^28^ measures the concentration of the CheY-CheZ complex [Yp-Z] formed during dephosphorylation, which is limited by the rate of kinase autophosphorylation^28^. Hence, the FRET signal is proportional to kinase activity^95^:

2 $$\,\mathrm{FRET}\,\propto [\,\mathrm{Yp}-{\rm{Z}}]=a\frac{{k}_{{\rm{A}}}}{{k}_{{\rm{Z}}}}\,\mathrm{[CheA]}\,\approx a\frac{{k}_{{\rm{A}}}}{{k}_{{\rm{Z}}}}{[\mathrm{CheA}]}_{{\rm{T}}},$$

where [CheA] is the concentration of kinases and *a* is the activity per kinase.

To compute kinase activity, each ratiometric FRET time series was normalized to the maximum FRET level measured by a saturating repellent stimulus: *a*(*t*) = FRET(*t*)/FRET_max_.

We ruled out that two-state switching cells have an anomalously low expression of chemoreceptor array components, by confirming that the maximum amplitude of the CheY-CheZ FRET signal, proportional to the amount of receptor–kinase complex (equation (2)), was comparable between the switching and non-switching cells (Extended Data Fig. 1d).

### Cluster intensity analysis

The CheY phosphatase, CheZ, is recruited to chemoreceptor arrays by binding to the short form of CheA, CheA_s_. Because this binding affinity is high, resulting in a long exchange time (~10 min), comparable with that of CheA within arrays^99^, and insensitive to array activity^100^, the CheZ-YFP fluorescence intensity can be used as a proxy for receptor cluster size. In a typical FRET experiment, the CheY-RFP/CheZ-YFP FRET pair is overexpressed to increase the FRET signal-to-noise ratio, causing background fluorescence to obscure the fluorescent foci corresponding to receptor clusters. However, once the induction of the FRET plasmid is lowered sufficiently, fluorescent foci become visible via traditional wide-field microscopy (Fig. 1 and Extended Data Fig. 2) in the expanse of the FRET signal-to-noise ratio. To extract the cluster size, the sample was screened for cells with one large fluorescent focus. Since receptor clusters are typically localized at the poles of the cells, the YFP fluorescence intensity *I* along the long axis of each cell (Supplementary Fig. 4) was extracted. To account for non-localized, cytoplasmic CheZ-YFP, the average background intensity was calculated as follows:

3 $$< I > ={\int }_{-\infty }^{\infty }{\int }_{-l/2}^{+l/2}I(x,y)\,{\rm{d}}x\,{\rm{d}}y,$$

Then, the cluster size is defined as

4 $$\Delta I={I}_{{\rm{m}}{\rm{a}}{\rm{x}}}- < I > ,$$

where *I*_max_ is the maximum of the fluorescence intensity along the long axis of the cell *I*.

### Switching analysis

Switching events were analysed automatically using a custom-made MATLAB script (Mathworks, version 2019b or newer). For each cell, attractant and repellent responses were detected automatically based on the timing and duration of the stimulus delivery. Next, the ratiometric FRET time series of each cell, after correcting for photobleaching, was normalized between one and zero based on the repellent and attractant response amplitudes, respectively. The FRET signal was then low-pass filtered using a 3-s moving-average filter, and switching events were detected as peaks in the derivative of the filtered signal. With each switching event, an amplitude (change in kinase activity), residence time (time until next event) and transition time (duration of the switch based on a fit of the form 1 − *e*^−1^) are associated. The switching behaviour of each cell was classified according to the amplitude and number of the switching events per cell. A two-state switching cell is defined as a cell with at least 65% of its transitions showing activity-level changes of at least 0.7 or 70% of total kinase activity—increasing these thresholds to 80% and 0.8, respectively, has only a marginal effect on the switching statistics (Supplementary Fig. 5). For each two-state switching cell, the bleaching correction is refined by another correction step using only the activity in either the *a* = 0 state, for cells with bias <0.5, or *a* = 1 state, for cells with bias of >0.5. The time series is then renormalized using the maximum activity level of the histograms during the two-state switching, and the amplitude, transition time and residence time associated with each switching event are extracted again. To verify that the automated switching analysis extracts events reliably, we generated mock time series that recapitulated the switching phenotype of Tsr-I214K and added Gaussian white noise to approximate the experimental signal-to-noise ratio. By comparing the transition and residence times of the mock time series to the times extracted by our automated analysis, we find that the relative uncertainty is minimal, even for low signal-to-noise ratios (Supplementary Fig. 6). Events occurred at a stable average frequency throughout the course of experiments (Supplementary Fig. 6). There were no strong correlations between the transition and residence times per cell (Supplementary Fig. 7). The variability between cells was estimated by convolving simulated transition and residence time distributions with Gaussian white noise to match the experimentally observed distributions (Supplementary Fig. 8).

### Numerical simulations of the conformational spread model

Conformational spread was modelled by a two-dimensional Ising model on an *L* × *L* lattice with free boundary conditions. Each lattice site represents an allosteric unit, whose conformational state was represented in the main text by an activity variable *a*_*i*_ ∈ {0, 1}. For the discussion here, we make the mapping *σ* = 2*a* − 1 and discuss the model in terms of the ‘spin variable’ *σ*, which takes one of two values: *σ* = 1 for active, and *σ* = −1 for inactive. The activity of the unit at site *i* is influenced by its *N*_*j*_-nearest neighbours through the coupling energy *J* a biasing field *H*_b_ and a ligand field *H*_L_, giving a Hamiltonian (total energy) ${\mathcal{H}}$ for this lattice (in units of *k*_B_*T*):

5 $${\mathcal{H}}=-J\mathop{\sum }\limits_{\left\langle ij\right\rangle }{\sigma }_{i}{\sigma }_{j}+({H}_{{\rm{b}}}/2+{H}_{{\rm{L}}}/2)\mathop{\sum }\limits_{i}{\sigma }_{i},$$

where $\left\langle ij\right\rangle$ indicates that the summation is over all the nearest-neighbour pairs in the lattice. The biasing and ligand fields (*H*_b_ and *H*_L_, respectively) were set to zero for most of our simulations, except where specified otherwise in the text (the field dependence of the switching phenotype is described in more detail in Supplementary Note 2.7). The probability of finding the lattice in a given configuration is then proportional to ${{\rm{e}}}^{-{\mathscr{H}}}$ and the ratio of probabilities *p*_*i*_(*σ*_*i*_) and *p*_*i*_(−*σ*_*i*_) for the *i*th site to be in state *σ*_*i*_ as opposed to −*σ*_*i*_ is

6 $$\frac{{p}_{i}({\sigma }_{i})}{{p}_{i}(-{\sigma }_{i})}={{\rm{e}}}^{-\Delta {\mathcal{H}}},$$

where $\Delta {\mathcal{H}}\equiv {\mathcal{H}}({\sigma }_{i})-{\mathcal{H}}(-{\sigma }_{i})$ is the change in the Hamiltonian on flipping *σ*_*i*_. We set the rate *ω*(*σ*_*i*_ → −*σ*_*i*_) for the unit at site *i* to flip from a state *σ*_*i*_ to the opposite state −*σ*_*i*_ as

7 $$\omega ({\sigma }_{i}\to -{\sigma }_{i})={\omega }_{0}\exp \left[-J{\sigma }_{i}\mathop{\sum }\limits_{j}^{{N}_{j}}{\sigma }_{j}+({H}_{{\rm{b}}}/2+{H}_{{\rm{L}}}/2){\sigma }_{i}\right]$$

where *ω*_0_ is the fundamental flipping frequency of a single lattice unit and *N*_*j*_ is the number of nearest neighbours, so as to satisfy (together with equations (5) and (6)) the detailed balancing condition:

8 $$\frac{{p}_{i}({\sigma }_{i})}{{p}_{i}(-{\sigma }_{i})}=\frac{\omega (-{\sigma }_{i}\to {\sigma }_{i})}{\omega ({\sigma }_{i}\to -{\sigma }_{i})}.$$

We note that the fundamental frequency *ω*_0_ corresponds to the rate of conformational transitions in an individual allosteric unit. Because this rate is unknown for the bacterial chemosensory array, in our simulations, we set this parameter to unity, implying that the simulated temporal statistics are expressed in units of the fundamental timescale 1/*ω*_0_.

The rates, as defined in equation (7), were used in a kinetic Monte Carlo scheme (essentially as in ref. ^101^, but modified to have free boundary conditions, as shown below), which uses one random number in every iteration to draw the time until the next flip, and another to determine which site flips. The lattice was an *L* × *L* lattice with free boundary conditions, such that the number of nearest neighbours *N*_*j*_ at each lattice site was

9 $${N}_{j}=\left\{\begin{array}{ll}2 & \,{\rm{at\; corners,}}\\ 3 & \,{\rm{at\; edges,}}\\ 4 & \,{\rm{otherwise.}}\end{array}\right.$$

To calculate the activity *a* of the lattice at each time point, we map the spin variable *σ*_*i*_ ∈ {−1, +1} at each site to an activity variable *a*_*i*_ ∈ {0, 1} as *a*_*i*_ = (*σ*_*i*_ + 1)/2 and take its mean across the lattice:

10 $$a=\frac{1}{{L}^{2}}\mathop{\sum }\limits_{i}{a}_{i}.$$

To simulate adaptation feedback, a bias field is defined in which each lattice unit has a specific bias field *H*_b,*i*_ that represents the effect of receptor methylation according to^39^

11 $${H}_{{\rm{b}},i}=\alpha ({m}_{i}-{m}_{0}),$$

where 0 < *m*_*i*_ < *M* is the methylation level of site *i* with maximum value *M* and *α* = 1*k*_B_*T* is the energy per methylation site^39^. We chose *M* = 64, corresponding to approximately eight receptor dimers per Ising lattice unit and eight methylation sites per dimer^102^. The methylation offset *m*_0_ is set to *M*/8 (ref. ^39^). All units with *a*_*i*_ = 1 (and *m*_*i*_ < *M*) experience demethylation at rate *k*_B_ and all units with *a*_*i*_ = 0 (and *m*_*i*_ > 0) are methylated with rate *k*_R_. This activity-dependent (de)methylation activity results in robust perfect adaptation to a steady-state activity level of *a*_0_ = *k*_R_/(*k*_B_ + *k*_R_). We defined these rates in terms of the fundamental frequency *ω*_0_ according to *k*_B_ = *k*_R_ = *n*_R_/*L*^2^ × *V*_R_/*ω*_0_ = 0.0015*ω*_0_, where *n*_R_ = 200 is the approximate number of adaptation proteins per cell^103^ and *V*_R_ = 1/10s is the maximum rate of the methylation reaction^104^, reflecting that the adaptation enzymes work at saturation^105^. Arrays were simulated for a sufficient time to reach the steady state (*a*_0_ ≈ 0.5) before the application of an external field *H*_L_ (representing chemoattractant ligand stimulation) was used to probe the array responses.

Simulations were implemented with Python 3.7 or newer, and single simulation runs for extended times were performed on regular desktop computers. Parallel computations were performed on the LISA cluster of the SURFsara national computing facility (Amsterdam). For the parallel runs, each parameter set was given a unique seed for random number generation. The resulting time series were sampled at regular intervals, and subsequently downsampled to approximate the acquisition frequency of experiments (1 Hz) relative to the array-level switching frequency (~10^−2^ Hz). Simulated activity time series were then further processed in the same way as experimental data to extract the temporal statistics of switching.

### Calibration of fundamental frequency

Along the isoline ${J}_{r}^{\,{\rm{iso}}}(L)$ (Fig. 3b), the timescale ratio *r* by definition remains constant, meaning 〈Δ*t*〉 = *r*〈*τ*〉. Combined with the calibration of the finite-size scaling for the transition time 〈*τ*〉_sim_ ≈ *L*^*b*^ through numerical simulations (Extended Data Fig. 7), and noting that timescales from experiments and simulations are related as ${\langle \Delta t\rangle }_{\exp }={\omega }_{0}^{-1}{\langle \Delta t\rangle }_{{\rm{sim}}}$, we obtain *ω*_0_ = *rc*_*τ*_*L*^*b*^/Δ*t*_exp_, where *c*_*τ*_ and *b* are constants based on the scaling analysis (Supplementary Note 2). The resulting dependence on *L* was very similar for both Tar and Tsr (Extended Data Fig. 8). Although our FRET measurements do not provide a direct estimate of *L*, we can motivate the approximate upper and lower bounds based on structural and biochemical findings in the literature between *L* = 17 and *L* = 30 (Supplementary Note 3). With these approximate limits, our scaling analysis yields a flipping timescale of individual allosteric units in the range 1/*ω*_0_ ≈ 15–35 ms (Extended Data Fig. 8).

### Determination of correlation length

The correlation length ${\mathcal{E}}(J)$ was determined from exponential fits to the correlation function of Ising arrays^106^:

12 $$c(r)=\overline{{\sigma }_{i}{\sigma }_{j}}={{\rm{e}}}^{\frac{-r}{{\mathcal{E}}(\,J)}},$$

where $\overline{{\sigma }_{i}{\sigma }_{j}}$ is the spin product averaged over all pairs with equal distance *r* = ∣*i* − *j*∣. For each unit *i*, *j*, pairs are sampled horizontally and vertically (for example, keeping either *i* or *j* constant, ignoring diagonal values). For each *J*, the correlation length ${\mathcal{E}}(J)$ is computed as the average correlation length of 48 independent array states.

### Statistics and reproducibility

Number of experiments and number of cells per experiments are described in the figure captions or in supplementary tables. Two-state switching cells were selected according to the criteria described above; otherwise, no data were excluded from the analysis. No method was used to predetermine the sample sizes. The investigators were not blinded to allocation during experiments and outcome assessment.

### Reporting summary

Further information on research design is available in the Nature Portfolio Reporting Summary linked to this article.

## Online content

Any methods, additional references, Nature Portfolio reporting summaries, source data, extended data, supplementary information, acknowledgements, peer review information; details of author contributions and competing interests; and statements of data and code availability are available at 10.1038/s41567-025-03158-3.

## Supplementary information

Supplementary Information Supplementary Notes 1–6, Figs. 1–8, Tables 1–16 and Equations (1)–(17).

Reporting Summary

## Data Availability

All FRET experimental data, including figure source data, are available via Figshare at 10.6084/m9.figshare.27824757 (ref. ^107^). Raw FRET images are available from the corresponding author upon request. Bacterial strains and plasmids are listed in Supplementary Tables 1 and 2, respectively, and are available from the corresponding author upon request.

## Code availability

All custom code for experimental analysis and for numerical simulations of the Ising model are available via Figshare at 10.6084/m9.figshare.27824757 (ref. ^107^).
