# Supplementary material for: Spontaneous switching in a protein signalling array reveals near-critical cooperativity
Source: Nat Phys. 2026 Jan 29;22(3):452–60. doi: 10.1038/s41567-025-03158-3 (PMC13019169; doi:10.1038/s41567-025-03158-3)
Supplement: Supplementary file 1 — Supplementary Notes 1–6, Figs. 1–8, Tables 1–16 and Equations (1)–(17). [file 41567_2025_3158_MOESM1_ESM.pdf]

nature physics

Supplementary information https://doi.org/10.1038/s41567-025-03158-3

Spontaneous switching in a protein signalling array reveals near-critical cooperativity

In the format provided by the authors and unedited

Contents

A Supplementary Notes **2**

1 Measurements of fluctuations and switching in chemosensory arrays using FRET 2

1.1 Two-state fluctuations in chemosensory arrays with ligand stimuli. . . . 2

1.2 Changing activity bias by genetic modification 2

1.3 Additional control experiments and analysis to exclude external ligand fluctuations 3

2 Finite-size scaling effect in Ising lattices 4

2.1 The Ising model in the thermodynamic limit can not explain spontaneous switching 4

2.2 Finite-size Ising models exhibit two-state switching near criticality . . . 5

2.3 Scaling of the critical coupling energy 5

2.4 Scaling of the residence and transition times 6

2.5 Scaling analysis of the experimental data 6

2.6 Scaling of the fundamental frequency ω_0 7

2.7 Field dependence of two-state switching in finite lattices 8

3 Array size estimation and calibration of the fundamental frequency ω_0 10

4 Combined effect of static and dynamic disorder on response times and fluctuations near criticality 11

5 Physiological modulation of proximity to the critical point 13

5.1 Physiological modulation via chemoreceptor mixing 13

5.2 Physiological modulation by array size or receptor interactions 14

5.3 Functional consequences of near-criticality on signal noise and exploration behavior 15

6 Robustness of criticality in non-equilibrium Ising models 16

B Supplementary Tables **17**

C Supplementary Figures **25**

A Supplementary Notes

1 Measurements of fluctuations and switching in chemosensory arrays using FRET

1.1 Two-state fluctuations in chemosensory arrays with ligand stimuli.

The *in vivo* signaling activity of chemosensory arrays is determined by the combined effects of sensory inputs to chemoreceptors and adaptation feedback via reversible covalent modifications (methylation/demethylation) of specific chemoreceptor residues. Two aspects are crucial for chemotactic signal processing: the amplitude of the array response, which is enhanced by cooperative protein-protein interactions within the array (*48*), and the response timescale, which must match that of downstream adaptation and behavior responses to effectively bias the bacterial random walk (*64, 65*).

The motile behavior of the individual *E. coli* cell is known to be highly variable over time (*38, 76, 77*), and numerous studies have implicated a role for the adaptation enzymes CheR and CheB in generating this variability through stochasticity in their enzymatic activity (*38, 75, 79, 80*). Recent single-cell FRET measurements confirmed strong temporal fluctuations of intracellular signaling activity (*34, 35*), but surprisingly, the largest temporal fluctuations were found in cells deleted for both CheR and CheB and expressing only a single chemoreceptor species (out of five in wildtype *E. coli*). Strikingly, in one of these studies (*34*) we discovered a small number of cells that demonstrated switch-like fluctuations, with the total kinase activity of the cell alternating between fully active and inactive states while the input ligand stimulus was held constant. Two-level switching in whole-cell kinase activity is surprising because it implies synchronous switching of very large molecular populations — *E. coli* chemoreceptors and their associated kinases are expressed at a level of $\sim 10^3 - 10^4$ copies per cell (*103*). Yet within that study, a deeper analysis of those unusual fluctuations and their possible mechanistic origins were precluded by the small sample size of two-level switching cells.

A possible explanation for these giant fluctuations is that they represent intrinsic fluctuations amplified by strong cooperativity within one large chemosensory array. However, it is also possible that other factors, such as spurious fluctuations in the applied ligand signal, are driving synchronous switching of a larger number of otherwise independent signaling arrays. This motivated our experiments to measure two-state fluctuations in the absence of any chemoeffectors.

1.2 Changing activity bias by genetic modification

To observe fluctuations in the absence of adaptation enzymes and chemoeffectors, we used a strategy of using chemoreceptor mutants that change the activity bias. The activity of the chemoreceptors in bacteria is determined by fast ligand binding and slow changes in the activity bias level through modifications of the chemoreceptors by a pair of adaptation enzymes (*102*). Upon attractant binding, the activity decreases, but activity-dependent modification of

the chemoreceptors provides effective negative feedback that restores the activity to its prestimulus level. This way, the wildtype chemosensory array faithfully returns to a steady-state activity bias of, on average, 0.3, but with variation between cells presumably due to expression level variation of the adaptation enzymes (*34*). In the absence of the adaptation enzymes, both chemoreceptors Tar and Tsr when natively expressed, have an activity bias close to 1 (*28*). Switching between the active and inactive array state is only observed if the activity bias has intermediate values and maximizes at 0.5. In our previous study on fluctuations in chemosensory activity (*34*), we used small amounts of chemoattractant to bring the activity bias to intermediate values to observe switching behavior. Here, we wanted to observe fluctuations in the absence of chemoeffectors and adaptation enzymes and therefore change the activity bias in a ligand-independent way.

The adaptation enzymes (CheR and CheB in *E. coli*) change the energy bias of the chemoreceptors by sequentially methylating and demethylating four conserved glutamate residues on chemoreceptor Tar and five in Tsr (*39, 48, 109*), where CheR-mediated methylation increases the activity bias and CheB-mediated demethylation decreases it. Previous studies identified genetic modifications of those sites that mimic methylation, and can modify the chemoreceptor activity bias without changing other chemoreceptor properties such as signal amplification. For both chemoreceptors the effect of methylation can be mimicked by exchanging a glutamate (E) for glutamine (Q). The fully unmethylated state (EEEE(E)) has an activity bias close to 0. For Tar, its QEEE modification state has an intermediate activity bias and shows fluctuations in the absence of chemoeffectors (*35*), and is used throughout this work.

We pursued mutations in Tsr that would generate an intermediate activity bias. The I214K mutation in the control cable region of Tsr was a promising candidate (*40*). In population-averaged FRET experiments, it showed an intermediate activity bias and similar dose–response curve parameters to L-serine as Tar [QEEE] to MeAsp. Therefore, we investigated the behavior of cells expressing only Tsr-I214K with single-cell FRET and observed similar switching behavior as in the case of Tar [QEEE].

In Tsr, the QEEEE modification state has a bias close to 1 (*109*), precluding it from being used for spontaneous switching (Fig. S2). We also examined additional receptor variants and found that Tsr [EEEEE] exhibits an intermediate activity bias and spontaneous switching at room temperature (22 C) (Fig. S2). Taken together, our observations indicate that switching behavior is not specific to the I214K mutation, but rather arises when the activity bias of a chemosensory array with near-critical cooperativity is close to the transition point between active and inactive array states

1.3 Additional control experiments and analysis to exclude external ligand fluctuations

We performed additional control experiments to exclude the influence of external noise sources. First, we ruled out that switching fluctuations are driven by ligands secreted by the cells themselves by tests on single-residue replacement mutants of the ligand binding pocket (residue 69) of both Tar [QEEE] and Tsr-I214K known to abolish ligand binding in both receptor

types (*110, 111*). Cells expressing these mutated receptors showed no response to their cognate ligands, as expected, but still exhibited switching behavior (Extended Data Figure 3a,b). Switching fluctuations were unimpeded by exogenous addition of pyruvate, a key metabolic intermediate that lies close to the entry point of carbon metabolism under our experimental conditions (with lactic acid as the sole carbon source), and recently shown to exhibit large temporal fluctuations in *E. coli* under some conditions (*108*) (Extended Data Figure 3c). Switching was also not correlated between cells (Extended Data Figure 3d), indicating that fluctuations in secreted metabolites are not the cause of chemosensory array switching. Finally, cells expressing Tsr-F396Y, a single-residue replacement mutant defective in response cooperativity, demonstrated no switching behavior (Extended Data Figure 4), indicating that the switching phenotype is linked to cooperativity within the array. Taken together, these observations suggest that two-state switching reflects a generic property of chemosensory arrays that is not specific to a single chemoreceptor species, and that it arises not from extrinsic fluctuations of ligand inputs or secreted metabolites, but rather from intrinsic fluctuations that drive cooperative switching of thousands of molecules within a single large chemosensory array.

2 Finite-size scaling effect in Ising lattices

2.1 The Ising model in the thermodynamic limit can not explain spontaneous switching

The two-dimensional Ising model (*112, 113*) is known to demonstrate a second-order phase transition as a function of the ratio J/k_BT where J is the coupling energy, T is the temperature, and k_B is Boltzmann's constant. Increasing J across a critical value J^* at fixed T (or equivalently, decreasing T below a critical value T^* at fixed J) leads to an abrupt ordering of the lattice for J above (or T below) this critical point, with the lattice becoming polarized with all spins in either the up or down states. For a two-dimensional lattice in the thermodynamic limit (where the lattice size $L \to \infty$), an exact solution of the model was obtained by Onsager (*114*), giving the precise critical value $J^*/k_BT = \ln(1 + \sqrt(2))/2 = 0.440686\ldots$ (and equivalently $k_BT^*/J = 2.26918\ldots$). Near this critical point, various thermodynamic observables (such as the correlation length ξ and the correlation time τ_c) are known to diverge as power laws characterized by critical exponents as a function of the "reduced temperature",

$$\epsilon \equiv |T - T^*|/T^* = |J^* - J|/J, \tag{S1}$$

which provides a dimensionless measure of the distance from the critical point (*115*). For example, the critical exponent ν ($= 1$ for 2-D lattices) defines how the correlation length diverges, $\xi \sim \epsilon^{-\nu}$, and another exponent z (≈ 2.2 for 2-D lattices (*60*)) in turn defines the scaling of the correlation time $\tau_c \sim \xi^z = \epsilon^{-\nu z}$, as the critical point is approached ($\epsilon \to 0$). The divergence of the correlation time at the critical point implies that above J^* (or equivalently below T^*), the system becomes frozen in one of the two polarized states. Thus, the Ising model in the thermodynamic limit is unable to explain the switching fluctutions we have observed in chemoreceptor arrays.

2.2 Finite-size Ising models exhibit two-state switching near criticality

By contrast to the behavior in the thermodynamic limit discussed above, in Ising lattices of finite size, the correlation length ξ can not grow beyond the lattice size L, and the scaling of various observables near criticality becomes a function of the system size L, a phenomenon known as finite-size scaling (*57, 116*). For example, the singularity in the correlation time scaling $\tau_c \sim \xi^z = \epsilon^{-\nu z}$ at the critical point ($\epsilon = 0$) is lost due to the cutoff of ξ at L and instead becomes $\tau_c \sim L^z$. Thus, fluctuations in near-critical 2-D Ising lattices of finite size demonstrate all-or-none switching between polarized states (intuitively, because $\xi \sim L$) that become increasingly slow as the lattice size grows (intuitively, because $\tau_c \sim L^z$) — an example of the phenomenon of "critical slowing down" observed across a broad range of dynamical systems near criticality. The excellent agreement between the temporal statistics of our experimental data with those of simulated Ising lattices (Fig. 2) suggested that both Tar and Tsr chemoreceptor arrays are well described as finite-size Ising systems close to criticality.

To quantitatively estimate the degree to which chemosensory arrays are close to criticality, we applied finite-size scaling to further analyze our experimental and simulated temporal statistics, as described below. The basic idea is to use the observed degree of near-critical slowing to estimate the distance to criticality, and (with appropriate calibrations) also obtain an estimate for the fundamental conformational timescale $1/\omega_0$ of allosteric units.

2.3 Scaling of the critical coupling energy

An important result of finite-size scaling theory is that the position of the critical point (*i.e.* the value of J^*, or equivalently, T^*) itself becomes a function of the system size L (*116*). As noted above, the well-known Onsager exact solution for the infinite two-dimensional Ising lattice (*114*) gives the critical coupling energy $J^*(\infty)$ explicity (in units of $k_B T$) as

$$J^*(\infty) = \frac{1}{2} \ln \left(1 + \sqrt{2}\right) \approx 0.44. \tag{S2}$$

For finite lattices there is no exact analytical solution, but both analytical (*61*) and numerical (*62*) approaches to finite-size scaling theory have established the dependence of the critical coupling energy J^* (or equivalently the critical temperature T^*) on the system size L. The dependence $J^*(L)$ of the critical coupling energy on system size is well approximated as (*61, 62*),

$$J^*(L) \approx \frac{J^*(\infty)}{1 - cL^{-1}}, \tag{S3}$$

where c is constant whose value and sign depend on the boundary conditions. For periodic boundary conditions, c is negative ($c_{p.b.c.} = -0.36$) and hence $J^*(L)$ approaches $J^*(\infty)$ from below as $L \to \infty$. By contrast, c is positive for free boundary conditions ($c_{f.b.c.} = +1.25$) and in this case $J^*(L)$ approaches $J^*(\infty)$ from above as $L \to \infty$. Given that chemoreceptor arrays cover only a finite area of the plasma membrane, they have open ends. In our simulations,

therefore, only free boundary conditions are considered, meaning that $J^*(L) > J^*(\infty)$ for all values of L.

2.4 Scaling of the residence and transition times

As noted above, for coupling energies close to the critical energy ($J \approx J^*$), the spatial correlation length of activity states approaches the lattice size L, leading to polarized all-or-none fluctuations. Thus, within this regime, the activity time series is well approximated as a random telegraph process (*117*), and hence the residence timescale Δt (i.e. time between switching events) is expected to be proportional to the correlation time τ_c (because $\tau_c = (\langle \Delta t_{\text{up}} \rangle^{-1} + \langle \Delta t_{\text{down}} \rangle^{-1})^{-1}$ for a random telegraph process). Numerical studies have found that a good approximation for the scaling of τ_c in finite lattices in the near-critical region, $\tau_c \sim L^z \exp[c_0 \epsilon L]$, with $\epsilon = |(J^*_\infty - J)/J|$ and the "dynamical critical exponent" $z = 2.2 \pm 0.1$ (*59, 60*). We confirmed that the residence times extracted from our simulations at various combinations of ϵ and L were well-fit by a scaling of the same form,

$$\Delta t = c_t L^z \exp[c_0 \epsilon L] \tag{S4}$$

with $z = 2.2$ (Extended Data Figure 7a), and these fits yielded for the remaining scaling constants $c_0 = 1.6 \pm 0.5$ and $c_t = 1.4 \pm 0.5$.

For the transition time τ (the duration of the activity transient upon switching), that we define as the simple average of t$\langle \tau_- \rangle$ and $\langle \tau_+ \rangle$, we found that our data were well-described (Extended Data Figure 7b) by a power-law,

$$\tau = c_\tau L^b \tag{S5}$$

with $c_\tau = 1$ and $b = 1.725$.

2.5 Scaling analysis of the experimental data

While the finite-size scaling relations obtained above provide an excellent approximation to the simulated data, they can not be directly applied to the experimentally observed timescales because the simulations yield timescales in units of the fundamental flipping timescale $1/\omega_0$, which remains unknown for the chemosensory array. That is, Δt_{sim} and τ_{sim} from simulations are related to Δt_{exp} and τ_{exp} from experiments as, respectively, *i.e.*,

$$\Delta t_{\text{sim}} = \Delta t_{\text{exp}} \omega_0, \text{ and } \tau_{\text{sim}} = \tau_{\text{exp}} \omega_0. \tag{S6}$$

We therefore identify as a key experimental observable the dimensionless timescale ratio $r \equiv \Delta t/\tau$, which removes the dependence on the unknown constant ω_0. Using Eqs. S4 and S5, we obtain for this ratio,

$$r = \frac{\Delta t}{\tau} = \frac{c_t}{c_\tau} L^{(z-b)} \exp(c_0 \epsilon L). \tag{S7}$$

This scaling provides a one-to-one relationship between r and the product ϵL, as evidenced by the collapse of simulated data at various combinations of ϵ and L onto a single curve (Fig. 3a Inset). However, because the size L is not precisely known for the chemoreceptor array, we can not uniquely determine ϵ.

We therefore chose to assess the distance from criticality by comparing iso-lines $J_r^{\text{iso}}(L)$ in the J-L plane, corresponding to parameter combinations that yield the same ratio $r = \Delta t/\tau$, against the curve for $J^*(L)$ given by the finite-size scaling of the critical coupling energy (Eq. S3). To obtain an expression for the iso-lines $J_r^{\text{iso}}(L)$, we first solved Eq. S7 for ϵ to obtain the reduced temperature along the isoline of constant r as a function of L,

$$\epsilon_r^{\text{iso}}(L) = \frac{\log\left[r/c_R\right] + (b-z)\log L}{c_0 L}, \tag{S8}$$

where we have defined $c_R \equiv c_t/c_\tau$. Plugging in the definition of the reduced temperature $\epsilon_r^{\text{iso}} \equiv \left|J^*(\infty) - J_r^{\text{iso}}\right| / J_r^{\text{iso}}$ and solving, we obtain,

$$J_r^{\text{iso}}(L) = \frac{J^*(\infty)}{1-\epsilon_r^{\text{iso}}} = \frac{J^*(\infty)}{1 - c_r^{\text{iso}}(L)/L}, \tag{S9}$$

where in the last step we have defined $c_r^{\text{iso}}(L) \equiv \left(\log\left[r/c_R\right] + (b-z)\log L\right)/c_0$.

The isolines $J_r^{\text{iso}}(L)$ from simulations (solid lines in Fig. 3b) were fit well by Eq. S9 (dashed curves in Fig. 3b), for experimentally determined r-values corresponding to both Tar (r_{Tar}=9.1) and Tsr (r_{Tsr}=12), with each fit parameter (Table S16) constrained to within one standard deviation of their scaling values estimated numerically (Extended Data Figure 7). Comparing Eq. S9 with Eq. S3, and noting that $c_r^{\text{iso}}(L)$ depends only weakly on L over the range of interest ($10 \lesssim L \lesssim 30$), clarifies why the isolines $J_r^{\text{iso}}(L)$ have nearly the same shape as $J^*(L)$ (gold curve in Fig. 3b). As a result, the ratio $J_r^{\text{iso}}(L)/J^*(L)$ remains nearly constant as a function of L for each value of r (Fig. 3b Inset), allowing us to quantify the proximity to criticality despite uncertainty in the exact value of L.

Finally, we note that given the observed asymmetry in the transition times τ for upward and downward transitions ($\langle\tau_+\rangle \neq \langle\tau_-\rangle$; Fig. 2h), there remains some ambiguity in the value of τ to be used in the ratio $r = \Delta t/\tau$. In lieu of a strong motivation to favor either, we have opted to impose symmetry by taking the average $(\langle\tau_+\rangle + \langle\tau_-\rangle)/2$, as noted in the Main Text. Choosing instead $\langle\tau_+\rangle$, $\langle\tau_-\rangle$, or any value in between would for Tar lead to values in the range $7.7 \lesssim r_{\text{Tar}} \lesssim 11$ and for Tsr, $10.7 \lesssim r_{\text{Tsr}} \lesssim 14$. Yet as can be gleaned from the logarithmic dependence of $c_r^{\text{iso}}(L)$ on r in Eq. S9, modifications to r_{Tar} and r_{Tsr} in that range would have only slight effects, with J_r^{iso} remaining confined to within a few percent of $J^*(L)$.

2.6 Scaling of the fundamental frequency ω_0

As noted above, the timescales determined from the simulations are dimensionless quantities (Δt_{sim} and τ_{sim}) expressed in units of the fundamental flipping timescale $1/\omega_0$ of individual lattice units (through Eq. 7 introduced in Methods). Thus, Δt_{sim} is related to experimentally

observed timescale Δt_{exp} as $\Delta t_{\text{sim}} = \Delta t_{\text{exp}}\omega_0$. Given the finite-size scaling relations obtained above, one could therefore estimate ω_0 as a function of L by comparing the timescales extracted from experiments and numerical simulations. To obtain such a scaling relation for ω_0, we use the fact that along the isolines $J_r^{\text{iso}}(L)$, the ratio $r = \Delta t/\tau$ remains constant, so Δt scales in the same way as τ. Plugging in the expression for the reduced temperature ϵ_r^{iso} along the isoline (Eq. S8) into the residence time scaling (Eq. S4) yields for the simulated residence time,

$$\Delta t_{\text{sim}} = c_t L^z \exp\left[\log\left(r c_\tau / c_t\right) + (b - z)\log L\right] = r c_\tau L^b, \tag{S10}$$

which indeed scales as the transition time ($\sim L^b$). From this we obtain the scaling relation for ω_0 as a function of the lattice size L and experimental residence timescale Δt_{exp}:

$$\omega_0 = \Delta t_{\text{sim}}/\Delta t_{\text{exp}} = r c_\tau \frac{L^b}{\Delta t_{\text{exp}}}. \tag{S11}$$

with the r corresponding to the experimentally obtained values of Tar and Tsr.

2.7 Field dependence of two-state switching in finite lattices

While we have shown that temporal statistics compatible with the experimentally observed two-state switching behavior can be generated by a near-critical Ising model with J close to J^*, proximity to the Ising critical point depends also on the biasing field H, which must vanish ($H \to 0$) at the critical point. It is therefore of interest to ask what ranges of H are compatible with two-state switching. Switching fluctuations are large collective excitations involving nearly all $N = L^2$ lattice units, and due to the fluctuation-dissipation relation (*118*),

$$\langle \sigma^2 \rangle - \langle \sigma \rangle^2 = k_B T \chi / L^2 \tag{S12}$$

where $\chi \equiv \partial |\langle \sigma \rangle| / \partial H$ is the field-dependent susceptibility, their likelihood can be assessed by the field-dependence of the activity bias $\langle a \rangle = (\langle \sigma \rangle + 1)/2$ of Ising lattices. Intuitively, switching is most likely where the activity variance $\langle a^2 \rangle - \langle a \rangle^2$ $(= (\langle \sigma^2 \rangle - \langle \sigma \rangle^2)/4)$ is greatest, which through Eqn. S12 is also where the susceptibility χ (and equivalently the absolute logarithmic activity derivative $|\partial \log\langle a \rangle / \partial H|$) is largest.

A biasing field H (in units of $k_B T$) modifies the flipping rate of a single spin $\omega(\sigma_i \to -\sigma_i)$ by a multiplicative factor $\exp(H\sigma_i/2)$ (Eqn. 7). Thus, a positive field ($H > 0$) pushes the bias towards the limiting value $\langle \sigma \rangle \to -1$ (equivalently $\langle a \rangle \to 0$), but there will always be a finite amplitude of fluctuations such that $\langle \sigma \rangle > -1$ (equivalently $\langle a \rangle > 0$).

For strong fields ($H \gg J$), fluctuations in activity will be dominated by single spin flips, and hence the activity bias will approach that expected for an arbitrary spin σ_i in the lattice,

$$\langle a \rangle \to \left\langle \frac{p(\sigma_i)}{p(\sigma_i) + p(-\sigma_i)} \right\rangle = \left\langle \frac{1}{1 + e^{\Delta \mathscr{H}}} \right\rangle \tag{S13}$$

where the averages are taken over all spins $\{\sigma_i\}$ and $\Delta\mathscr{H} \equiv \mathscr{H}(\sigma_i) - \mathscr{H}(-\sigma_i)$ is the change in the Hamiltonian (Eqn. 5) upon flipping σ_i. Given that the $\Delta\mathscr{H} \approx H$ for strong fields, the activity bias reduces to

$$\langle a \rangle \approx \frac{1}{1+e^H}, \text{ for } H \gg J \tag{S14}$$

which decays with derivative $\partial \log \langle a \rangle / \partial H = \chi / 2 \langle a \rangle \approx -1$ characteristic of this single spin-flip dominated regime.

In the limit of weak coupling ($J \to 0$), Eqn. S14 becomes an exact identity valid for all values of H, and the derivative $\partial \log \langle a \rangle / \partial H \to -1/2$ as $H \to 0$.

Conversely, in the limit of strong coupling ($J \to \infty$), the system approaches the MWC limit (*49, 119, 120*), for which the activity bias becomes instead

$$\langle a \rangle = \frac{1}{1+e^{NH}} \tag{S15}$$

with $N = L^2$ is the system size, and valid at all finite values of H. The corresponding derivatives $\partial \log \langle a \rangle / \partial H \approx -N$ for strong fields ($e^{NH} \gg 1$) and $\partial \log \langle a \rangle / \partial H \to -N/2$ for weak fields ($H \to 0$), respectively.

Near criticality where $J \approx J^*$, and $0 < H/J \ll 1$ the activity bias in the thermodynamic limit ($L \to \infty$) is known to scale as a power law (*121*),

$$\langle a \rangle = \frac{1}{2}(1 - H^{1/\delta}) \tag{S16}$$

with $\delta = 15$ for two-dimensional lattices, which gives a steep field dependence $\partial \log \langle a \rangle / \partial H \approx -\delta^{-1} H^{(1/\delta - 1)}$ that diverges as $H \to 0$.

In finite-size Ising lattices, this singularity is rounded to give a finite size-dependent maximal steepness at $H = 0$ that scales as a power law in L,

$$|\partial \log \langle a \rangle / \partial H|_{H=0} = |\partial \log \langle a \rangle / \partial H|_{\text{max}} = \chi_{\text{max}} \sim L^x \tag{S17}$$

with an exponent x in the range $1.75 \leq x \leq 2.0$ depending on J/J^* (*118*). Thus, the steepness at zero field grows as $\sim L^x$ with system size, and the range ΔH of the field exhibiting this steep dependence narrows as $\sim L^{-x}$.

Despite the steep dependence at small $|H|$ ($< \Delta H$), it has been shown that in finite Ising lattices (with periodic boundary conditions) near criticality this steepness decays sharply as H increases beyond ΔH. As a result, the response curve of $\langle a \rangle$ vs H develops a substantial tail that contrasts starkly to that in the strongly coupled MWC limit (*46*). Our simulations (on finite lattices with free boundary conditions, $L = 20$, $J = 0.475 k_B T \approx J^*(L)$, Extended Data Figure 5c) also show a similar trend: a steep dependence of $\langle a \rangle$ on H for $H < 0.03$ that then deviates strongly to develop a substantial tail before crossing over eventually to the expected scaling for the single-spin dominated regime $\partial \log \langle a \rangle / \partial H \to -1$ at $H \approx 0.3$ (Extended Data Figure 5c, Inset). Thus, there exists a substantial range in H beyond the steep region ΔH (≈ 0.03 for

$L = 20$ and $J \approx J^*(L)$) exhibiting collective excitations. While we expect switching to be most prominently observed within the steep region $|H| < \Delta H$, they can also occur within this intermediate regime ($0.03 < H < 0.3$) of mildly attenuated collective excitations, albeit with reduced frequency.

Nevertheless, taking as a conservative estimate the width ΔH of the steep region as the range of switching behavior for the $L = 20$ lattice, an exponent of $x = 2$ for the finite-size scaling of the switching range, i.e. $\Delta H \sim L^{-2}$, our estimated size range $L = 17$-30 for chemosensory arrays our experiments (see Main Text) implies that the biasing field is no greater than $|H| < \Delta H \approx 0.01$-$0.04 k_B T$ in cells demonstrating two-state switching. Combined with the fact that two-state switching requires a value of J near its critical value ($J \approx J^* \approx 0.44 k_B T$), this suggests that the strength of any biasing field in these cells is at most 3-10% of the coupling energy.

Note that for *E. coli* chemoreptor arrays the activity bias in the absence of ligand is known to depend on temperature (*95*), and in our experiments we have deliberately set the temperature T to a value that gives the largest fraction of two-state switcher cells (see Methods and Supplementary Figure 1). Thus, the population-average value μ_H of the biasing field H is set through this procedure to be very close to zero. We also observed (Fig. 1c and Main Text) that approximately two thirds of cells with a single large cluster demonstrated two-state switching, whereas most of the remaining third or so showed no switching. If we interpret the non-switcher cells as having an effective biasing field outside of the two-state switching regime discussed above (*i.e.* $|H| > \Delta H$) and further assume for simplicity a normal distribution $N(\mu_H, \sigma_H^2)$ of H across the population, the above considerations suggest an average $\mu_H \approx 0$ and standard deviation $\sigma_H \approx 0.01$-$0.04 k_B T$ (given that for a normal distribution approximately two thirds of the population lies within $\mu_H \pm \sigma_H$).

3 Array size estimation and calibration of the fundamental frequency ω_0

Although our FRET measurements do not provide a direct estimate of L, we can motivate approximate upper and lower bounds based on structural and biochemical findings in the literature. Cryo-EM studies have revealed the detailed ultrastructure of bacterial chemosensory arrays (*32, 33, 122*), revealing an extended regular arrangement (Extended Data Figure 8) of 'core units', the smallest complex of array components that has shown kinase activity *in vitro* (*123*) and *in vivo* (*124*), consisting of one CheA kinase dimer, two CheW scaffolding protein monomers and two trimers of chemoreceptor dimers. Expression data under defined growth conditions (*103*) suggest an approximate number of 1000 core units per cell, close in number to a lattice of size $L \times L = 30 \times 30$, which we take as an approximate upper bound. However, it is also possible that the fundamental unit of cooperativity is an even larger complex than the core units. For example, taking instead the repeating unit cell of the array structure's p6 symmetry group (Fig. 4) as the allosteric unit leads to ≈ 300 repeating units per cell, or approximately $L \times L = 17 \times 17$, which we take as an approximate lower bound.

With these approximate limits, our scaling analysis yields a flipping timescale of individual

allosteric units in the range $1/\omega_0 \approx$ 15-35 ms (Extended Data Figure 8). The importance of protein structural dynamics for function is increasingly recognized (*125*), but estimates of the transition timescales are usually obtained via *in vitro* measurements (*126–128*) or molecular dynamics simulations (*129*), and span an enormously wide range (from picoseconds to milliseconds). Our *in vivo* estimate for the chemoreceptor array allosteric unit lies near the upper extreme of that range, perhaps reflecting the large size of allosteric units (even the core unit, the smaller of the two limits considered here, contains 16 protein monomers).

Interestingly, our finite-size scaling estimate of the fundamental allosteric transition timescale ω_0^{-1} ($\approx$ 15-35 ms) is close to the timescale of CheA autophosphorylation (*130*), which operates out of equilibrium by hydrolyzing ATP. It is therefore plausible that in chemosensory arrays, the dynamics of allosteric cooperativity are driven by an underlying nonequilibrium process involving CheA (Supplementary Note 6).

4 Combined effect of static and dynamic disorder on response times and fluctuations near criticality

Ligand-response cooperativity of chemosensory arrays maps in the Ising model framework to the susceptibility χ to an applied external field ($\chi \equiv \partial a/\partial H$). Near criticality, χ scales with the correlation length ξ as $\chi \sim \xi^{\gamma/\nu}$, where γ and ν are critical exponents ($\gamma = 7/4$ and $\nu = 1$ for 2-D) whereas the correlation time τ_c that defines the degree of critical slowing scales as $\tau_c \sim \xi^z$ ($z \approx$ 2.2 for 2-D) (*60*). Thus, $\tau_c \sim \chi^{\nu z/\gamma} \approx \chi^{1.25}$, meaning that any changes to system parameters that attenuate response cooperativity (*i.e.* leading to reduced χ), can be expected to mitigate critical slowing of both flucttuation and response (as reflected in τ_c) more or less proportionally.

Chemosensory arrays of wildtype cells are affected not only by the standard Ising model parameters (J, H, L), but also by two additional kinds of spatial disorder that contribute additional variables relevant to near-critical dynamics:

- **Static disorder due to mixing of different receptor species within the array.** *E. coli* possesses five chemoreceptor genes (*tar, tsr, tap, trg, aer*), but they are not all expressed in equal amounts. The two 'major' chemoreceptors (*tsr* and *tar*) are expressed at much higher levels compared to the other three 'minor' chemoreceptors (*102*). Considering that arrays form by stochastic self-assembly (*36*), in wildtype cells Tar and Tsr receptors are expected to be randomly scattered across the array. And in such mixed arrays, it is known that the cooperativity of ligand response is attenuated relative to arrays with only one receptor type (*48*), suggesting that the coupling energy J likely takes different values between nearest neighbors depending on whether the neighbouring sites are occupied by the same species or not. Within the Ising model, this situation corresponds to the family of random-bond Ising models (RBIM), familiar in spin glass physics, where the coupling energy J becomes a random variable: $J = \langle J \rangle + \delta J$, with $\langle \delta J \rangle = 0$ and $\langle \delta J^2 \rangle = \sigma_J^2$. It is known that RBIMs can retain a ferromagnetic-paramagnetic transition for weak disorder,

but even in this regime, the critical temperature T^* decreases (or equivalently, critical coupling energy J^* increases) as the disorder σ_J is increased (*131, 132*). Thus, while the presence of this static bond disorder can shift the distance from the critical point, it does not eliminate near-critical behavior and consideration of this disorder-dependent distance from criticality is crucial for understanding the behavior of wildtype cells with different degrees of receptor mixing (and hence different disorder strengths σ_J) at different stages of growth (as shown by (*68, 69*) and exemplified in this study through experiments with WT cells harvested at different stages of growth, OD=0.2 and OD=0.45).

- **Dynamic disorder injected by the adaptation system into the array.** In wildtype cells, the adaptation system implements negative feedback by addition and removal of covalent modifications (methyl groups) to maintain a stable average activity level. These enzymatic modification reactions occur stochastically at random sites, introducing another kind of disorder in the array. Within the Ising model, this situation can be captured by the family of random-field Ising models (RFIM), where the local biasing field, H_b at each site becomes a random variable: $H_b = \langle H_b \rangle + \delta H_b$, with $\langle \delta H_b \rangle = 0$ and $\langle \delta H_b^2 \rangle = \sigma_{H_b}^2$. In contrast to RBIMs, RFIMs do not exhibit a critical point in the thermodynamic limit (i.e. the infinite-size limit), because the correlation length becomes limited to a disorder-dependent lengthscale (known as the Imry-Ma length, L_{IM} (*133*). However in reality, the system size is always finite, and if the array size L falls below the Imry-Ma length ($L < L_{\text{IM}}$), the system can still exhibit a transition between effectively ferromagnetic and paramagnetic phases. Experimentally, even in arrays with only a single receptor species (which we have shown to be near-critical without the adaptation system), it is known that cooperativity of ligand response is attenuated in the presence of the adaptation system, suggesting that the dynamic local field-disorder injected by the adaptation system is sufficient to bring the Imry-Ma length below the array size ($L_{\text{IM}} < L$), thereby alleviating deleterious critical slowing (Figs. 5 and 6 and Extended Data Figure 9).

These general insights regarding RBIMs and RFIMs provide a basis for interpreting our measurements of steady-state fluctuations in wildtype cells (Extended Data Fig. 10e-h), which demonstrated a striking difference in amplitude between physiological conditions with a low degree of receptor mixing (and hence low σ_J, when harvested at OD=0.2) and a high degree of receptor mixing (and hence high σ_J, when harvested at OD=0.45). The fact that varying the degree of bond disorder σ_J leads to large differences in fluctuation amplitude suggests that despite a finite level of both bond disorder ($\sigma_J > 0$) and field disorder ($\sigma_{H_b} > 0$), wildtype chemosensory arrays remain within the near-critical regime. More specifically, the very large amplitude of fluctuations spanning nearly the full range of activity $0 < a < 1$ (Extended Data Figure 10) adaptation-induced Imry-Ma lengthscale L_{IM}, which limits fluctuation amplitudes due adaptation-induced field disorder σ_{H_b}, is evidently not far below the array size L so that the correlation length ξ of fluctuations can grow close to L when the bond disorder σ_J due to receptor mixing is sufficiently low.

5 Physiological modulation of proximity to the critical point

A compelling open question is how *E. coli* chemoreceptor arrays achieve the observed near-critical cooperativity, and if/how cells can physiologically tune proximity to the critical point. The strength of conformational interactions in canonical allosteric oligomers such as hemoglobin are usually assumed to be determined by their three-dimensional structure, which is in turn encoded by their amino acid sequence. On the one hand, it is possible that the nearest-neighbor interaction strength J is similarly "hard-coded" in bacterial chemosensory arrays at the level of protein structures, which would imply tuning through natural selection over evolutionary timescales. On the other hand, J and/or L might be physiologically regulated.

We first discuss the role of chemoreceptor species mixing in tempering near-critical dynamics (by adding disorder to the value of J across the lattice). We then discuss the possibility that the average coupling strength (J) and/or the array size (L) might also be regulated. Finally, we discuss the functional consequence of near-critical singal fluctuations on exploration performance of swimming *E. coli* cells.

5.1 Physiological modulation via chemoreceptor mixing

The receptor population within chemosensory arrays typically comprise multiple chemoreceptor species, allowing cells to integrate distinct signals, but at the cost of reduced cooperativity (*48*). The expressed chemoreceptor population of wildtype *E. coli* cells is known to vary across growth phases of cultured cells, with Tsr dominating the population early on (at low opitcal density, OD), and an increased Tar/Tsr ratio at later phases (at high OD) of batch-culture growth (*68, 69*). This change in the expressed chemoreceptor population makes the chemosensory response cooperativity dependent on the growth phase at which cells are harvested (*34*). Therefore, harvesting cells at different ODs allows tests of wildtype array properties at different degrees of chemoreceptor mixing.

We measured the effect of receptor mixing on response timescales. Adaptation-deficient cells with a wildtype complement of chemoreceptors harvested at OD=0.20 demonstrated substantial response delays (≈ 15 s) to sub-saturating stimuli (Fig. 5c). By contrast, when cells of the same genotype were harvested at OD=0.45, the response delay was reduced to ≈ 2 s (Fig. 5d), that is, an order of magnitude increase in response speed relative to the pure Tsr arrays (Fig. 5a). The response to saturating stimuli in adaptation deficient cells was faster than the response to subsaturating stimuli for each genotype (Fig. 5). Evidently, homogeneous arrays exhibit the strongest response slowdown, but even when all chemoreceptor genes are present, the array's response can be very slow, depending on chemoreceptor ratios. without some mechanism to mitigate this response slowdown, chemotactic performance would be severely impaired.

Our results reveal another mechanism that enables physiologically tuning of the distance to criticality. By changing the degree of chemoreceptor mixing, cells can tune the degree of static disorder (Supplementary Note 4). Consistent with this view, we found above that the degree of critical slowing in adaptation-deficient cells is reduced at later stages of growth (OD=0.45) when the degree of receptor mixing is high, compared to earlier stages of growth (OD=0.2)

when receptor mixing is low (Fig. 5c). Moreover, the same adaptation-deficient cells at the low receptor-mixing growth stage (OD=0.2) exhibited binary switching fluctuations whereas at the high receptor-mixing growth stage (OD=0.45) they did not (Extended Data Figure 10c,d), further supporting the idea that cells can physiologically modulate proximity to criticality. Modulation of near-critical dynamics was also observable in wildtype cells with an intact adaptation system, where the amplitude of continuous-level signal fluctuations were substantially augmented in cell populations with low degrees of receptor mixing (OD=0.2) compared to populations with high degrees of mixing (OD=0.45) (Extended Data Figure 10e-h), with the fraction of cells with very high noise strength $\eta \equiv \sigma_a/a_0$ reaching $\eta \approx 0.88$ at low mixing (OD=0.2) compared to $\eta \approx 0.7$ at high mixing (OD=0.45). These fluctuation amplitudes exceed by ~ 3- to 4-fold previous theoretical predictions of models that did not account for near-critical cooperativity of arrays (*38, 39, 75, 79, 80*).

5.2 Physiological modulation by array size or receptor interactions

Another possibility of physiological regulation of homogeneous arrays (one chemoreceptor species) is by increasing the array size (L in the model) or by directly changing the receptor-receptor coupling (J in the model). This could be mediated by the expression level of scaffolding protein CheW, which has recently been shown to affect both the array composition and apparent response cooperativity at the population level (*134*). Here we discuss the possibility if such tuning can be inferred from the switching statistics in combination with the measured chemoreceptor cluster size.

It has been shown that chemosensory array sizes vary strongly between cells (*36, 37*). Because CheZ is known to localize to the arrays (Fig. 1b), in our FRET experiments with reduced FRET pair expression, the arrays are visible as fluorescence intensity peaks above the cytoplasmic CheZ-YFP background (clusters). We can integrate fluoresce intensity over the cluster area to obain a 'cluster size' measure that serves as a proxy for the true array size (Fig. S4a and Methods). These experiments revealed that the average cluster size in cells exhibiting only a single detectable cluster was smaller for cells exhibiting two-state switching than for cells exhibiting no switching (one-state) ($p = 0.004$, two-sample KS test), consistent with the expectation that switching events become increasingly rare with increasing array size L (at a given J).

It is also possible to estimate the array size L using the temporal statistics we measured by FRET and the Ising finite-size scaling theory. This can be done in two ways, depending on different assumptions that can be made about J under cell-to-cell variations in L. One possibility is that J remains constant under variations in L. If that is the case, L can be calculated by plugging in the measured residence timescale $\langle \Delta t \rangle$ of each cell scaled by ω_0 into Eqn. S11 and solving for L. Otherwise, J varies together with L in such a way that the timescale ratio r (≈ 12 for Tsr) remains constant, in which case, a relationship between L and Δt can be found

by solving Eq. (S11) to obtain,

$$L = \left(\frac{\omega_0 \Delta t}{c_\tau r}\right)^{1/b} .$$

In Fig. S4b, these two estimation methods (constant J and varying J) were used to convert transform the $\langle \Delta t \rangle$-distribution from FRET experiments into the distribution of array size. Parameters were the same as those used in Fig. 3 (and listed in Table S16) ($z = 2.1$, $b = 1.75$, $c_0 = 1.18$) and those obtained in in Sections 2.2.3 and 2.2.4 of the Supplementary Note 2 ($c = 1.25$, $c_t = 1.4$, $c_\tau = 1$). For ω_0, we chose a value in the center of the range given in Fig. 4 ($1/\omega_0 = 21$ ms). Data for the $\langle \Delta t \rangle$-distribution were the same as those shown in Fig. 2d for Tsr-I214K, restricted to the activity-bias range $-0.25 \leq \Delta G/k_{\mathrm{B}}T \leq 0.25$.

Fig. S4b compares the distribution of cluster intensities for those cells exhibiting only one cluster but showing two state switching, to the distribution of array size estimated from two-state switching statistics. The shapes of the distributions for each finite-size scaling estimation method (constant J and varying J) and from CheZ-YFP cluster intensity are indistinguishable within experimental error (Fig. S4b).

5.3 Functional consequences of near-criticality on signal noise and exploration behavior

Numerous experimental and theoretical studies have established that swimming *E. coli* cells can exploit such signal fluctuations to enhance the exploratory propensity of their run-and-tumble motility by converting an otherwise Brownian-type random walk (with an exponential distribution of run times τ_{run}, which occurs at low η) into a Lévy-type random walk (with a power-law distribution of τ_{run}, which occurs at high η) (*38, 75–78*).

Theoretically, the range of the power law in the distribution of run intervals τ_{run} is predicted (*75*) to extend over a range $\mathcal{R} \equiv \tau_{\text{run}}^{\text{high}}/\tau_{\text{run}}^{\text{low}}$ that scales with the signal noise strength η as $\mathcal{R} \sim \exp[g\eta]$, where g is a gain coefficient of the flagellar motor response to the intracellular signal, which is known to be very high ($g \approx$ 10-20 (*135, 136*)). If we take $\eta = 0.7$ (which at $g = 10$ extends the power law range $\mathcal{R}$ over three orders of magnitude) as a nominal threshold for Lévy-type exploratory behavior, our signal noise measurements across wildtype cell populations (Extended Data Figure 10h) indicate that physiological modulation of near-critical dynamics during growth allows for a substantial shift in the fraction of exploratory cells ($\eta > 0.7$) within the population, with around half of cells exhibiting the exploratory high-noise phenotype ($P(\eta > 0.7) \approx 0.51$) at early stages of growth (OD=0.2), compared to around a third ($P(\eta > 0.7) \approx 0.29$) at later stages of growth (OD=0.45).

Thus, in addition to balancing the speed-amplitude tradeoff, near-critical cooperativity affects environmental exploration of wildtype *E. coli* cells by augmenting steady-state signal noise that promotes exploratory behavior. Furthermore, the fraction of cells exhibiting strong noise to drive exploratory behavior is evidently modulated physiologically, by changing the degree to which near-critical dynamics are tempered by static disorder due to receptor-species mixing within chemosensory arrays.

6 Robustness of criticality in non-equilibrium Ising models

While we have shown that nearly all features of switching temporal statistics can be explained within the framework of the canonical equilibrium Ising model (Fig. 2), we have noted that the single feature not captured by our equilibrium Ising modeling, namely asymmetry in the transition times for the up- and down-directions of activity switching (Fig. 2h), suggests a breaking of time-reversal symmetry — a hallmark of nonequilibrum dynamics (*54, 55*). Interestingly, our finite-size scaling estimate (Extended Data Figure 8) of the fundamental allosteric transition timescale ω_0^{-1} ($\approx$ 15-35 ms) is close to the timescale of CheA autophosphorylation (*130*), which operates out of equilibrium by hydrolyzing ATP. It is therefore plausible that in chemosensory arrays, the dynamics of allosteric cooperativity are driven by an underlying nonequilibrium process involving CheA. While exploration of such nonequilibrium effects not explicitly accounted for in the present study represents an exciting direction for future work, we expect our main finding that chemosensory arrays operate near criticality to remain a robust result. This is because kinetic Ising models with spin-flip dynamics are known to retain the same near-critical scalings as the equilibrium Ising model (*i.e.* they remain in the Ising universality class), even when driven out of equilibrium (*137, 138*). Indeed, recent theoretical studies (*88–90*) indicate that the switching statistics observed in our experiments point to near-critical cooperativity also when the Ising framework is generalized to include nonequilibrium effects

B Supplementary Tables

Table S1: Strains used in this study. EDF: Extended Data Figure. MCP: all methyl-accepting chemotaxis proteins. Genotype refers to changes compared to parent strain *E. coli* RP437.

**Name**	**Relevant genotype**	**Plasmid I**	**Plasmid II**	**Source**	**Figures used**
TSS58	ΔfliC ΔCheRBYZ	pSJAB106	pZR1	(*34*)	EDF10
TSS1845	FliC* ΔCheRBYZ	pSJAB106	pBAD33	This Study	FIG5
TSS1846	FliC* ΔCheYZ	pSJAB106	pBAD33	This Study	EDF9 and EDF10
TSS1964	FliC* ΔMCP ΔCheRBYZ	pSJAB106	receptor allele (see Table S2)	(*34*)	rest of this study

Table S2: Plasmids used in this study. Amp: 100 μg/mL ampicillin; Cam: 34 μg/ml chloramphenicol.

**Name**	**Product**	**Vector**	**Induction**	**Resistance**	**Source**
pVS120	Tar [QEEE]	pLC113	2.0 μM NaSal	cam	(*48*)
pSJAB191	Tar [QEEE]/R69H	pLC113	2.0 μM NaSal	cam	This study
pPA114	Tsr-WT	pKG116	0.6 μM NaSal	cam	(*67*)
pPA114 I214K	Tsr-I214K	pKG116	0.6 μM NaSal	cam	(*40*)
pSJAB193	Tsr-I214K/R69E	pKG116	0.6 μM NaSal	cam	This study
pPA114 F396Y	Tsr-F396Y	pKG116	0.6 μM NaSal	cam	(*139*)
pPA114 QEEEE	Tsr [QEEE(E)]	pKG116	0.6 μM NaSal	cam	(*109*)
pPA114 EEEEE	Tsr [EEEE(E)]	pKG116	0.6 μM NaSal	cam	(*109*)
pZR1	FliC*	pKG116	3.0 μM NaSal	cam	(*34*)
pBAD33	empty vector	pBAD33	-	cam	(*140*)
pSJAB106	CheZ-5G-YFP CheY-5G-mRFP1	PTrc99a	15/50/100 μM IPTG (see methods)	amp	(*34*)

Table S3: Mean residence times and number of events extracted from cells expressing Tsr-I214K.

Bias	$\langle \Delta t_{\text{up}} \rangle$ (s)	± std	$\langle \Delta t_{\text{down}} \rangle$ (s)	± std	N_{up}	N_{down}
$0.65 < \alpha \leq 1$	116.2 ± 3.27	± 101.8	38.6 ± 0.79	± 26.2	967	1091
$0.55 < \alpha \leq 0.65$	72.4 ± 2.31	± 61.2	48.0 ± 1.20	± 32.6	703	733
$0.45 < \alpha \leq 0.55$	60.4 ± 1.84	± 50.9	59.6 ± 1.68	± 46.6	766	767
$0.35 < \alpha \leq 0.45$	56.7 ± 2.36	± 47.2	80.7 ± 3.48	± 68.2	398	384
$0 \leq \alpha \leq 0.35$	41.3 ± 1.65	± 27.9	118.9 ± 6.56	± 104.8	285	255
$0 \leq \alpha \leq 1$	78.2 ± 1.36	± 75.9	57.0 ± 0.95	± 53.9	3119	3230

Table S4: Mean transition times and number of events extracted from cells expressing Tsr-I214K.

Bias	$\langle \tau_{\text{up}} \rangle$ (s)	± std	$\langle \tau_{\text{down}} \rangle$ (s)	± std	N_{up}	N_{down}
$0.65 < \alpha \leq 1$	4.28 ± 0.10	± 3.16	6.09 ± 0.12	± 3.49	970	837
$0.55 < \alpha \leq 0.65$	4.31 ± 0.12	± 3.21	6.07 ± 0.15	± 3.57	660	572
$0.45 < \alpha \leq 0.55$	4.18 ± 0.12	± 3.02	5.89 ± 0.14	± 3.54	681	604
$0.35 < \alpha \leq 0.45$	4.36 ± 0.17	± 3.16	6.18 ± 0.20	± 3.60	362	320
$0 \leq \alpha \leq 0.35$	4.49 ± 0.20	± 3.24	6.35 ± 0.22	± 3.32	262	235
$0 \leq \alpha \leq 1$	4.29 ± 0.06	± 3.15	6.07 ± 0.07	± 3.52	2935	2568

Table S5: Mean residence times and number of events extracted from cells expressing Tar [QEEE].

Bias	$\langle \Delta t_{\text{up}} \rangle$ (s)	± std	$\langle \Delta t_{\text{down}} \rangle$ (s)	± std	N_{up}	N_{down}
$0.65 < \alpha \leq 1$	79.4 ± 2.87	± 70.6	28.2 ± 0.58	± 14.8	607	647
$0.55 < \alpha \leq 0.65$	56.5 ± 2.15	± 41.5	37.4 ± 1.22	± 23.7	373	379
$0.45 < \alpha \leq 0.55$	44.7 ± 1.66	± 30.7	45.3 ± 1.68	± 31.1	343	341
$0.35 < \alpha \leq 0.45$	39.0 ± 2.06	± 29.6	58.0 ± 3.56	± 50.1	207	198
$0 \leq \alpha \leq 0.35$	32.0 ± 1.33	± 18.0	86.0 ± 5.94	± 77.0	183	168
$0 \leq \alpha \leq 1$	57.5 ± 3.03	± 52.7	42.6 ± 1.95	± 39.3	1713	1733

Table S6: Mean transition times and number of events extracted from cells expressing Tar [QEEE].

Bias	$\langle \tau_{\text{up}} \rangle$ (s)	± std	$\langle \tau_{\text{down}} \rangle$ (s)	± std	N_{up}	N_{down}
$0.65 < \alpha \leq 1$	4.70 ± 0.14	± 3.22	5.73 ± 0.15	± 3.33	556	501
$0.55 < \alpha \leq 0.65$	5.13 ± 0.18	± 3.39	6.00 ± 0.21	± 3.42	346	271
$0.45 < \alpha \leq 0.55$	4.71 ± 0.20	± 3.50	6.39 ± 0.20	± 3.18	301	260
$0.35 < \alpha \leq 0.45$	4.81 ± 0.27	± 3.53	6.26 ± 0.28	± 3.35	176	140
$0 \leq \alpha \leq 0.35$	4.56 ± 0.24	± 3.09	6.61 ± 0.31	± 3.49	171	130
$0 \leq \alpha \leq 1$	4.79 ± 0.08	± 3.34	6.06 ± 0.09	± 3.35	1550	1302

Table S7: Fit parameters of mean residence times per cell as a function of energy bias ΔG.

	slope γ_{down}	slope γ_{up}	crossover point $\tau_{\text{up}} = \tau_{\text{down}}$	N
Tar [QEEE]	-0.44	0.45	47.0 ± 1 s	204
Tsr-I214K	-0.39	0.45	65.5 ± 1 s	549

Table S8: Mean residence times and number of events extracted from numerical simulations using a conformational spread model with a lattice size of 20 × 20 spins and coupling energy J = 0.475 $k_B T$.

External Field	Bias	$\langle \Delta t_{\text{up}} \times \omega \rangle$	± std	$\langle \Delta t_{\text{down}} \times \omega \rangle$	± std	N_{up}	N_{down}
H = -0.006	$\alpha \approx$ 0.79	4790 ± 381.43	± 4110	1250 ± 76.81	± 840	116	121
H = -0.002	$\alpha \approx$ 0.63	2820 ± 172.44	± 2220	1680 ± 95.29	± 1240	166	168
H = 0	$\alpha \approx$ 0.51	2190 ± 128.34	± 1600	2130 ± 149.60	± 1870	155	156
H = 0.002	$\alpha \approx$ 0.44	1930 ± 97.63	± 1280	2440 ± 155.67	± 2040	172	171
H = 0.006	$\alpha \approx$ 0.23	1370 ± 82.68	± 920	4480 ± 359.98	± 3890	123	117
		2185 ± 90.52	± 1597	2132 ± 87.50	± 1868	732	733

Table S9: Mean transition times and number of events extracted from numerical simulations using a conformational spread model with a lattice size of 20 × 20 spins and coupling energy J = 0.475 $k_B T$.

External Field	Bias	$\langle \tau_{\text{up}} \times \omega \rangle$	± std	$\langle \tau_{\text{down}} \times \omega \rangle$	± std	N_{up}	N_{down}
H = -0.006	$\alpha \approx$ 0.79	179.76 ± 10.35	± 111.51	169.54 ± 11.05	± 118.01	116	114
H = -0.002	$\alpha \approx$ 0.63	169.18 ± 8.83	± 113.11	166.95 ± 9.16	± 115.50	164	159
H = 0	$\alpha \approx$ 0.51	166.57 ± 8.95	± 106.98	156.20 ± 9.24	± 113.49	143	151
H = 0.002	$\alpha \approx$ 0.44	184.13 ± 9.74	± 124.39	155.91 ± 7.38	± 95.05	163	166
H = 0.006	$\alpha \approx$ 0.23	154.32 ± 8.79	± 91.73	159.81 ± 10.54	± 113.00	109	115
		171.59 ± 8.79	± 111.49	161.30 ± 10.54	± 110.41	695	705

Table S10: Mean residence times and number of events extracted from numerical simulations using a conformational spread model with a lattice size of 12 $\times$ 12 spins and coupling energy J = 0.5 $k_B T$.

External Field	Bias	$\langle \Delta t_{up} \times \omega \rangle$	$\pm$ std	$\langle \Delta t_{down} \times \omega \rangle$	$\pm$ std	N_{up}	N_{down}
H = -0.02	$\alpha \approx 0.85$	2230 $\pm$ 96.18	$\pm$ 2120	390 $\pm$ 11.34	$\pm$ 250	486	491
H = -0.006	$\alpha \approx 0.66$	1040 $\pm$ 45.69	$\pm$ 930	520 $\pm$ 21.05	$\pm$ 430	412	410
H = 0	$\alpha \approx 0.53$	800 $\pm$ 31.53	$\pm$ 650	700 $\pm$ 31.20	$\pm$ 640	419	420
H = 0.006	$\alpha \approx 0.40$	610 $\pm$ 24.81	$\pm$ 510	920 $\pm$ 38.20	$\pm$ 780	416	415
H = 0.02	$\alpha \approx 0.22$	520 $\pm$ 27.07	$\pm$ 640	1810 $\pm$ 69.06	$\pm$ 1610	551	546
		904 $\pm$ 27.60	$\pm$ 1318	1045 $\pm$ 22.34	$\pm$ 1067	2284	2282

Table S11: Mean transition times and number of events extracted from numerical simulations using a conformational spread model with a lattice size of 12 $\times$ 12 spins and coupling energy J = 0.5 $k_B T$.

External Field	Bias	$\langle \tau_{up} \times \omega \rangle$	$\pm$ std	$\langle \tau_{down} \times \omega \rangle$	$\pm$ std	N_{up}	N_{down}
H = -0.02	$\alpha \approx 0.85$	56.05 $\pm$ 1.89	$\pm$ 40.04	57.59 $\pm$ 1.97	$\pm$ 41.06	450	434
H = -0.006	$\alpha \approx 0.66$	58.07 $\pm$ 1.94	$\pm$ 38.02	60.16 $\pm$ 2.11	$\pm$ 41.10	385	380
H = 0	$\alpha \approx 0.53$	59.27 $\pm$ 2.17	$\pm$ 43.31	54.42 $\pm$ 1.87	$\pm$ 37.14	398	394
H = 0.006	$\alpha \approx 0.40$	58.84 $\pm$ 1.98	$\pm$ 38.48	57.51 $\pm$ 2.14	$\pm$ 41.90	378	382
H = 0.02	$\alpha \approx 0.22$	60.04 $\pm$ 1.99	$\pm$ 44.15	54.77 $\pm$ 1.60	$\pm$ 36.41	492	516
		58.46 $\pm$ 0.89	$\pm$ 41.03	56.76 $\pm$ 0.86	$\pm$ 39.43	2103	2106

Table S12: Fit parameters of exponential fits to residence times extracted from cells expressing Tsr-I214K. Exponential fit of the form $\alpha e^{\beta x}$.

Bias	Δt_{up}	Δt_{down}
$0.65 < \alpha \leq 1$	α = 0.0083<br>β = -0.0088	α = 0.0460<br>β = -0.0329
$0.55 < \alpha \leq 0.65$	α = 0.0177<br>β = -0.0166	α = 0.0289<br>β = -0.0227
$0.45 < \alpha \leq 0.55$	α = 0.0249<br>β = -0.0221	α = 0.0216<br>β = -0.0185
$0.35 < \alpha \leq 0.45$	α = 0.0310<br>β = -0.0273	α = 0.0139<br>β = -0.0122
$0 \leq \alpha \leq 0.35$	α = 0.0423<br>β = -0.0318	α = 0.0073<br>β = -0.0065

Table S13: Fit parameters of exponential fits to residence times extracted from cells expressing Tar [QEEE]. Exponential fit of the form $\alpha e^{\beta x}$.

Bias	Δt_{up}	Δt_{down}
$0.65 < \alpha \leq 1$	α = 0.0154<br>β = -0.0150	α = 0.0862<br>β = -0.0504
$0.55 < \alpha \leq 0.65$	α = 0.0231<br>β = -0.0199	α = 0.0480<br>β = -0.0342
$0.45 < \alpha \leq 0.55$	α = 0.0360<br>β = -0.0284	α = 0.0317<br>β = -0.0243
$0.35 < \alpha \leq 0.45$	α = 0.0556<br>β = -0.0403	α = 0.0227<br>β = -0.0194
$0 \leq \alpha \leq 0.35$	α = 0.0746<br>β = -0.0473	α = 0.0143<br>β = -0.0142

Table S14: Fit parameters of exponential fits to residence times extracted from numerical simulations using a conformational spread model with a lattice size of 20 $\times$ 20 spins and coupling energy of J = 0.475 $k_B T$. Exponential fit of the form $\alpha e^{\beta x}$.

Bias	Δt_{up}	Δt_{down}
H = -0.006	α = 0.2280<br>β = -0.2339	α = 3.3210<br>β = -1.5900
H = -0.002	α = 0.5589<br>β = -0.4987	α = 1.4930<br>β = -1.0050
H = 0	α = 0.7966<br>β = -0.6830	α = 1.3090<br>β = -0.9488
H = 0.002	α = 0.8822<br>β = -0.6558	α = 0.6276<br>β = -0.5173
H = 0.006	α = 1.0670<br>β = -0.9160	α = 0.1771<br>β = -0.0278

Table S15: Fit parameters of exponential fits to residence times extracted from numerical simulations using a conformational spread model with a lattice size of 12 $\times$ 12 spins and coupling energy of J = 0.5 $k_B T$. Exponential fit of the form $\alpha e^{\beta x}$.

Bias	Δt_{up}	Δt_{down}
H = -0.02	α = 0.5876<br>β = -0.5844	α = 3.3820<br>β = -3.4340
H = -0.006	α = 1.4200<br>β = -1.2040	α = 2.4260<br>β = -2.3460
H = 0	α = 2.1750<br>β = -1.6820	α = 2.0490<br>β = -1.7660
H = 0.006	α = 3.3800<br>β = -2.3630	α = 1.0920<br>β = -1.0250
H = 0.02	α = 4.1970<br>β = -3.2720	α = 0.8545<br>β = -0.8116

Table S16: Parameters that describe the scaling of the timescale ratio with lattice size and critical energy (Eq. S8). Each parameter is determined individually from the scaling behavior of residence and transition times (Supplementary Figure 8). For the scaling relation of the phase diagram (Fig. 3), the finite scaling relation was fitted to the iso lines corresponding to Tar, where each fit parameter was allowed to vary according to the fit uncertainty of the individual fits.

Parameter	From fitting (Fig. S8)	Used in iso-line fit (Fig. 3)	Literature
z	2.2	2.1	2.2 ± 0.1 (*60*)
b	1.725 ± 0.032	1.75	
c_1	0.294 ± 0.366		
$c_2 = c_0$	1.608 ± 0.466	1.18	
$c_t = e^{c_1}$	1.4 ± 0.5		
c_τ	1 (assumed, not fit)		
$c_R = c_t/c_\tau$	1.03± 0.23	0.8	

C Supplementary Figures

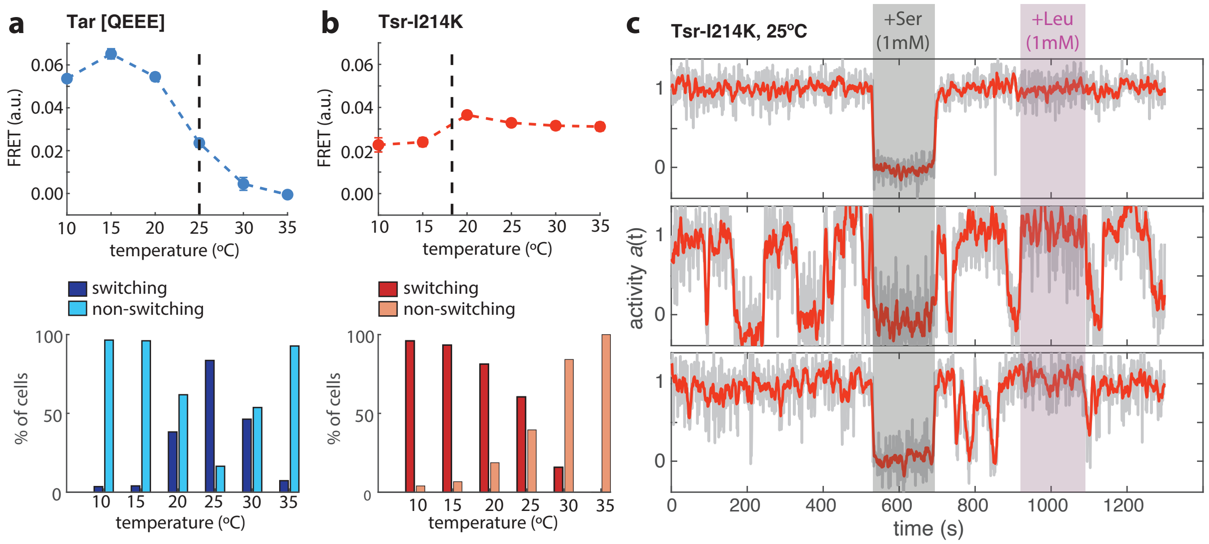

Figure **S1**: **Temperature dependence of switching dynamics (a)** (Top) Temperature dependence of the steady-state population-averaged FRET signal of cells expressing only Tar [QEEE]. Error bars indicate standard deviation of the steady-state population-averaged FRET signal. Error bars indicate (Bottom) Fraction of cells exhibiting switching of cells expressing only Tar [QEEE]. The vertical black dashed line indicates the temperature chosen in all Tar [QEEE] experiments in this manuscript (25°C). Switching was defined as the presence of strong, discrete transitions rather than two-state fluctuations and was determined manually. Cell counts for the six temperatures were: 142 (10°C), 126 (15°C), 89 (20°C), 140 (25°C), 54 (30°C), and 55 (35°C) **(b)** As in a, but for cells expressing only Tsr-I214K. The vertical line indicates the temperature used in all Tsr-I214K experiments (18°C), except for cluster imaging (room temperature). Cell counts were: 176 (10°C), 165 (15°C), 155 (20°C), 86 (25°C), 76 (30°C), and 22 (35°C). **(c)** Representative FRET timeseries of cells expressing Tsr-I214K at 25°C. The FRET timeseries were normalized by the responses to 1 mM serine and 1 mM Leucine as saturating, respectively, attractant and repellent stimuli. This experiment confirms that Tsr-I214K switches at 25°C, the temperature used for experiments with cells expressing Tar[QEEE].

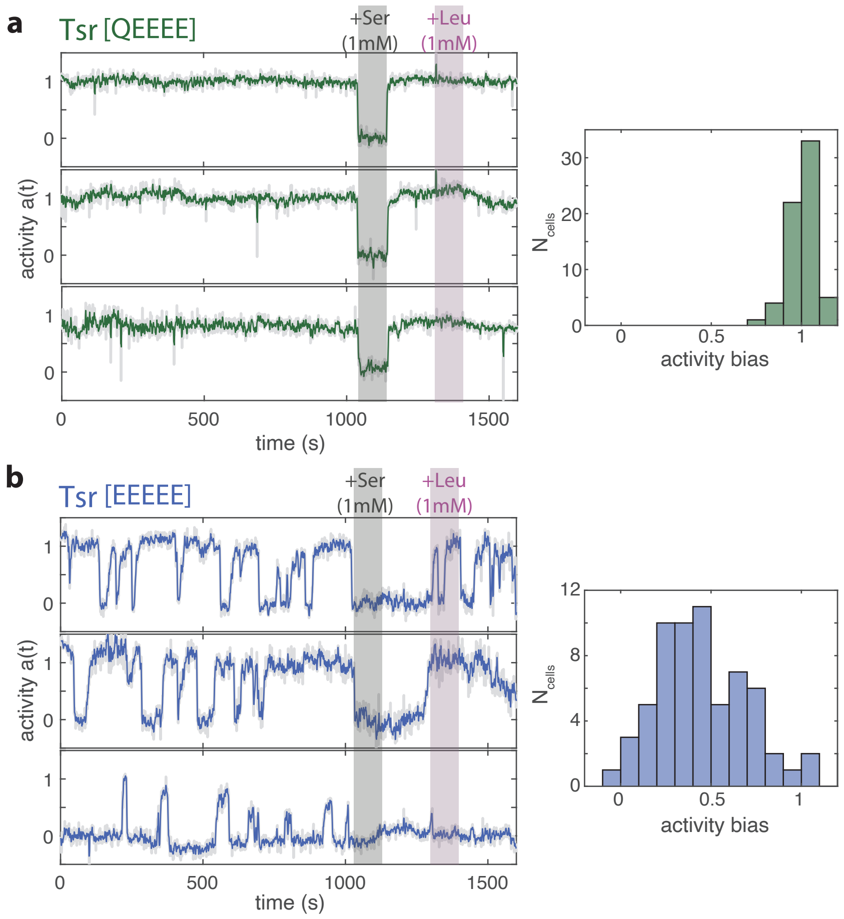

Figure **S2**: **Experiments with different Tsr modification states. (a)** 3 representative FRET timeseries (left) from an experiment with cells expressing Tsr [QEEEE], and the activity bias distribution of all (N=66) cells from the same experiment (right). This experiment revealed no switching dynamics and a activity bias close to 1. **(b)** 3 representative FRET timeseries (left) from an experiment with cells expressing Tsr [EEEEE], and the activity bias distribution of all (N=65) cells from the same experiment (right). This experiment revealed switching dynamics and intermediate activity bias. Experiments were performed at room temperature (22°C).

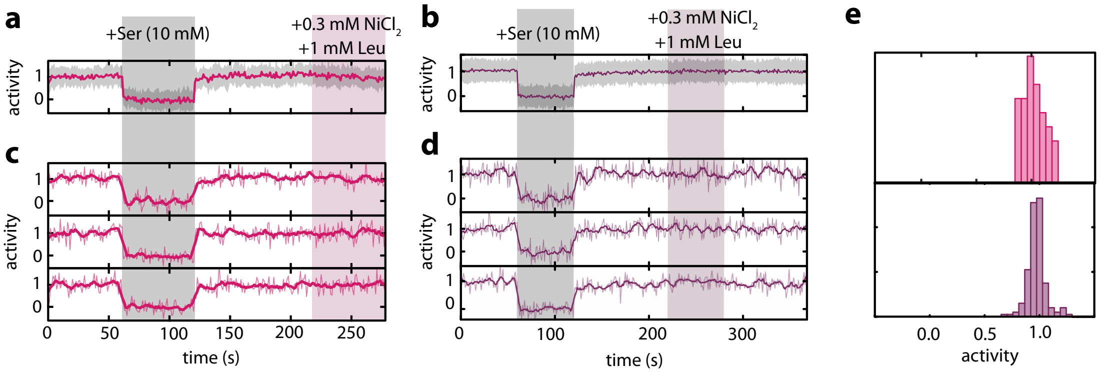

Figure **S3**: **Activity baseline is equal to 1 for non-adapting cells expressing WT receptor complement. (a,b)** Normalised population FRET time series (bold) and standard deviation of single-cell time series (gray area) for cells harvested at OD=0.20 (a, 40 cells) and OD=0.45 (b, 162 cells). FRET normalized between 0 and 1 using saturating attractant (10 mM L-serine) and repellent (0.3 mM $NiCl_2$ and 1 mM Leucine) stimuli, demonstrates that the baseline activity of the population and of individual cells (in the absence of any stimuli) is equal to 1. **(c,d)** Three examples of raw normalised single-cell FRET time series (faded curve) are superimposed on a low-pass filtered version of these curves using a 7 second time window, for cells harvested at OD=0.20 (c) OD=0.45 (d). **(e)** Histograms of the steady-state activity level (in the absence of any stimuli) for OD=0.20 (top) and OD=0.45 (bottom).

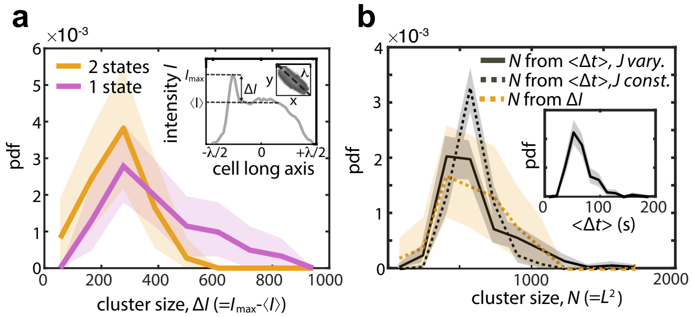

Figure **S4**: **Comparison of cell-to-cell variability in measured cluster size and array size inferred from switching statistics. (a)** Distribution of chemosensory cluster size, determined by CheZ-YFP fluorescence intensity, in 33 cells exhibiting a single Tsr-I214K cluster and demonstrating two-state switching ("2-states", solid yellow curve) and 55 cells exhibiting a single Tsr-I214K cluster but demonstrating no switching ("1-state", solid purple curve). Clusters were rendered detectable by lowering FRET plasmid induction below that in our standard FRET protocol to decrease cytoplasmic fluorescence and thereby allow visualization of CheZ-YFP localization (Fig. 1c, Extended Data Figure 2). Consistent with expectations from the finite-size Ising model (in which switching events become increasingly rare for larger array sizes L at a given coupling energy J), the average cluster size of cells exhibiting no switching (mean $\pm$ s.e.m. $=$ 388 $\pm$ 23) is significantly larger than the average cluster size of cells exhibiting two-state switching (mean $\pm$ s.e.m. $=$ 250 $\pm$ 17). These two distributions are statistically significantly distinct (two-sided Kolmogorov-Smirnov, $p = 0.0041$). Inset illustrates the method used for determining the cluster size. Cluster size is defined as the difference ΔI between the maximum CheZ-YFP intensity I_{max} along the long axis of each cell of length λ and its average intensity $\langle I \rangle$ (Methods). **(b)** Distribution of the array size inferred from cell-to-cell variation in two-state switching temporal statistics, compared against the measured cluster-size distribution of two-state switching cells of panel **a** (dashed yellow curve). Inferred array sizes were calculated in two ways (see Supplementary Note 5). First, by using the finite-size scaling relation (Eqn. S3) to transform the distribution of the average two-state switching residence times $\langle \Delta t \rangle$ per cell (inset), assuming J co-varies with L such that the timescale ratio remains constant, as in Fig. 3b (solid curve: mean $\pm$ s.d. $=$ 608 $\pm$ 251). Second, as in the first method but assuming J remains constant at $J = 0.426\ k_{\text{B}}T$, close to $J^*(\infty)$. (dashed dark curve: mean $\pm$ s.d. $=$ 593 $\pm$ 130). The inset shows the average two-state switching residence times $\langle \Delta t \rangle$ of 116 cells expressing Tsr-I214K and with a near-zero activity bias (corresponding to data points falling within the range $-0.25 k_{\text{B}}T \leq -\Delta G \leq 0.25 k_{\text{B}}T$ in Fig. 2d, upper panel). To enable proportional comparison, the cluster intensity is linearly scaled to match the average of the means of the two distributions of $N = L^2$. Shaded areas in all plots represent 97.5% confidence intervals obtained through bootstrap resampling.

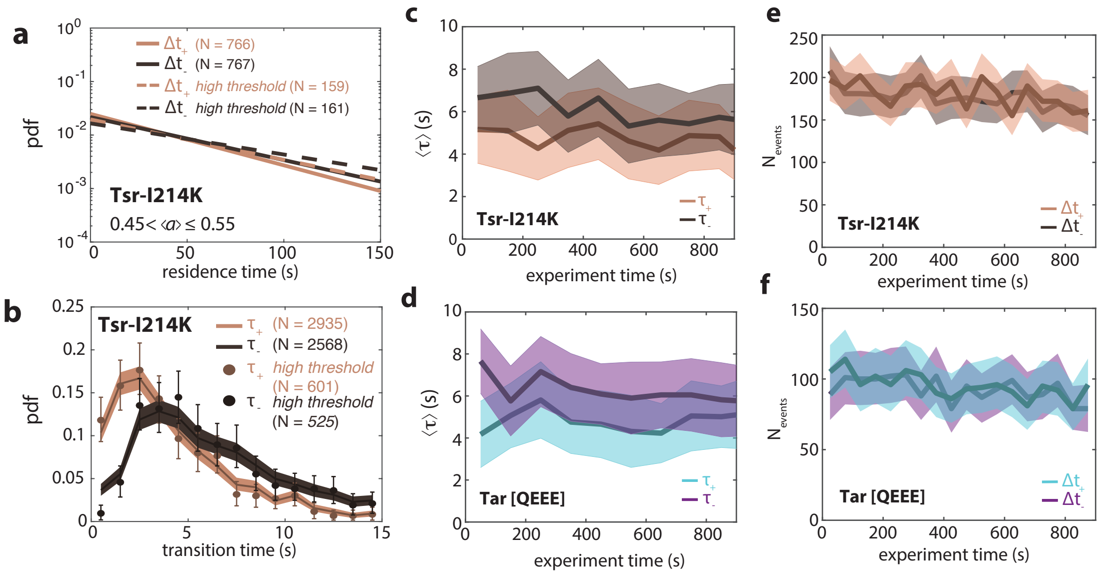

Figure **S5**: **Stability and robustness of switching statistics.** **(a)** Dependence of extracted residence times on threshold choice. Solid lines: single exponential fit to residence times for Tsr-I214K for cells with $a \approx 0.5$. The selection parameter for two-state switching cells throughout the paper is that more than 65% of the cell's transitions exhibit activity level changes exceeding 70% of the full-scale amplitude. Increasing both thresholds to 80% only has a marginal effect on the fitted distribution (dashed lines). Increased thresholds yield the following average residence times: Δt_{up} = 62.40 ± 3.73 s (mean ± s.e.m.) and Δt_{down} = 63.26 ± 3.36 s. For comparison, regular thresholds yield: Δt_{up} = 60.37 ± 1.84 s and Δt_{down} = 59.61 ± 1.68 s. **(b)** Dependence of extracted transition times on threshold choice. Increased thresholds yield the following average transition times: τ_{+} = 4.10 ± 0.12 s and τ_{-} = 6.07 ± 0.15 s. For comparison, regular thresholds yield: τ_{+} = 4.29 ± 0.06 s and τ_{-} = 6.07 ± 0.07 s. Shaded areas and error bars represent 95% confidence intervals obtained through bootstrap resampling. **(c)** Mean upwards (orange) and downwards (brown) transition times for switching events plotted as a function of their incidence time, for cells expressing Tsr-I214K. Shaded areas represent standard deviation. Mean transition times are stable throughout the duration of each experiment. **(d)** Mean upwards (turqoise) and downwards (purple) transition times for switching events plotted as a function of their incidence time, for cells expressing Tar[QEEE]. Shaded areas represent standard deviation. Mean transition times are stable throughout the duration of each experiment **(e)** Total number of upwards (orange) and downwards (brown) switching events with respect to their incidence time, for cells expressing Tsr-I214K. Shaded areas represent 95% confidence intervals obtained through bootstrap resampling. The frequency of transitions is constant throughout the duration of each experiment. **(f)** Total number of upwards (turquoise) and downwards (purple) switching events with respect to their incidence time, for cells expressing Tar[QEEE]. Shaded areas represent 95% confidence intervals obtained through bootstrap resampling. The frequency of transitions is constant throughout the duration of each experiment.

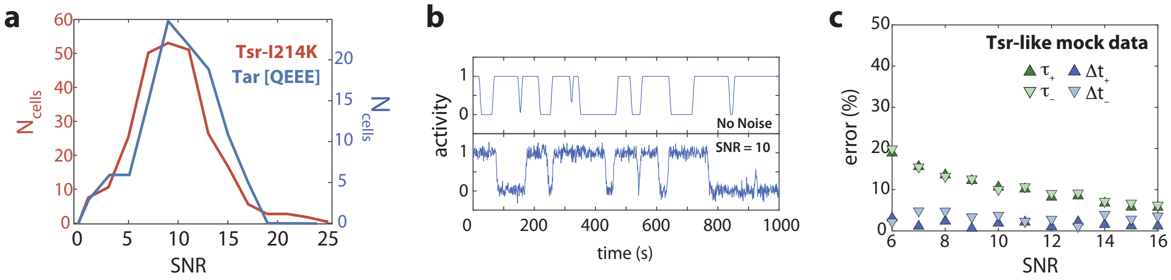

Figure **S6**: **Effect of experimental noise on extracting transition and residence times. (a)** Signal-to-noise ratio (SNR) for two-state switching cells calculated as the maximum FRET response divided by the standard deviation of fluctuations in the FRET signal during attractant response. **(b)** Mock-time series generated by sampling residence times from an exponential distribution and assuming one transition time (top panel) with Gaussian white noise added to simulate experimental noise (bottom panel). **(c)** Relative uncertainties for transition times τ and residence times Δt, based on analyzing mock two state time series with added Gaussian white noise. The simulated time series are based on the average transition and residence times measured for Tsr-I214K.

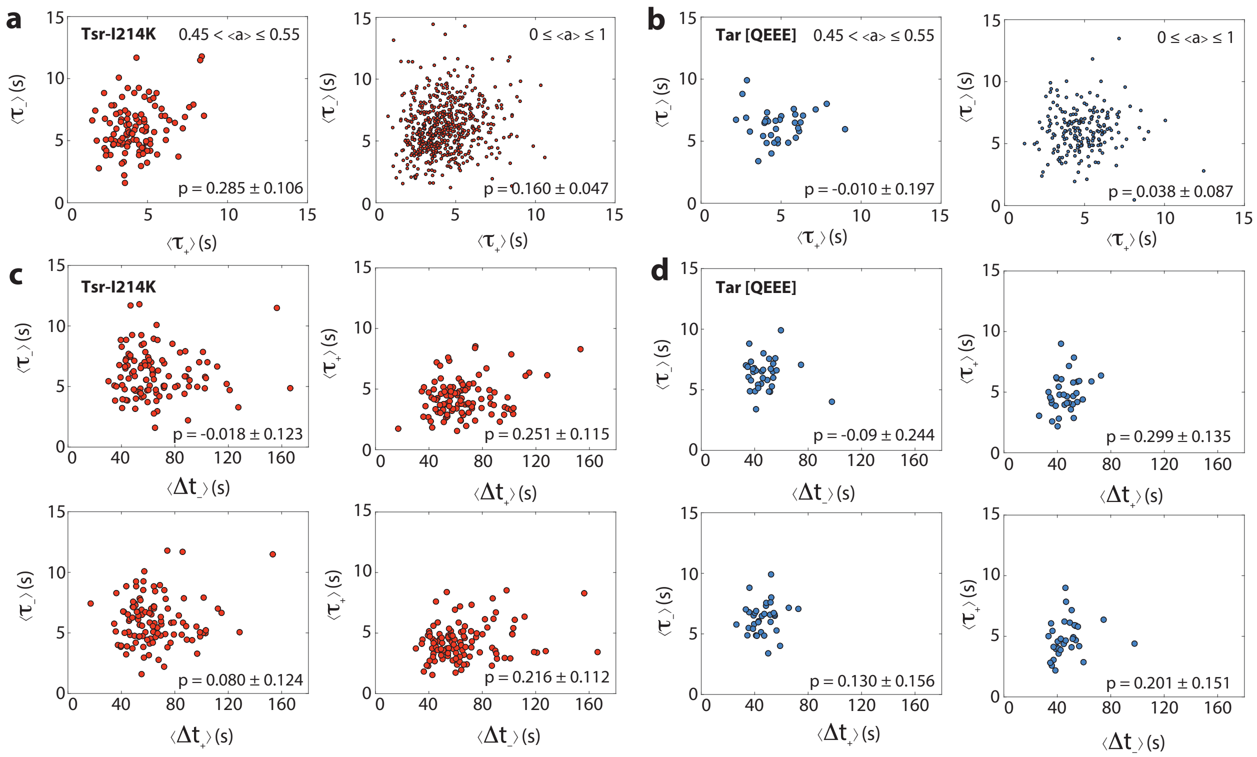

Figure **S7**: **Apparent correlations among switching timescale parameters.** **(a)** Correlations between upwards and downwards transition times for cells expressing Tsr-I214K (red points). Error margins in Pearson correlation coefficients are 95% confidence intervals obtained through bootstrap resampling. **(b)** as in (a) but for cells expressing Tar [QEEE] (blue points). **(c)** Correlations between transition and and residence times for cells that have an intermediate activity bias and express Tsr-I214K (red points). Error margins in Pearson correlation coefficients are 95% confidence intervals obtained through bootstrap resampling. Only small strength of association is observed between these switching parameters. **(d)** as in (c) but for cells expressing Tar [QEEE] (blue points). **(e)** Measured transition time vs residence time for (Left) 108 cells expressing Tsr-I214K from FRET experiments, with activity $0.45 \leq \langle a \rangle \leq 0.55$, and (Right) for 300 simulated cells. For each simulated cell, residence times and transition times were drawn respectively from an exponential and gamma distribution with parameters extracted from the experiments (Fig. 2 and Extended Data Figure 6), where the sum of residence times was limited to the experimental duration (1000 s). The variation in the simulation represents the sampling error that is observed without variation in parameters between cells. **(f)** As in (e) but for cells expressing Tar [QEEE].

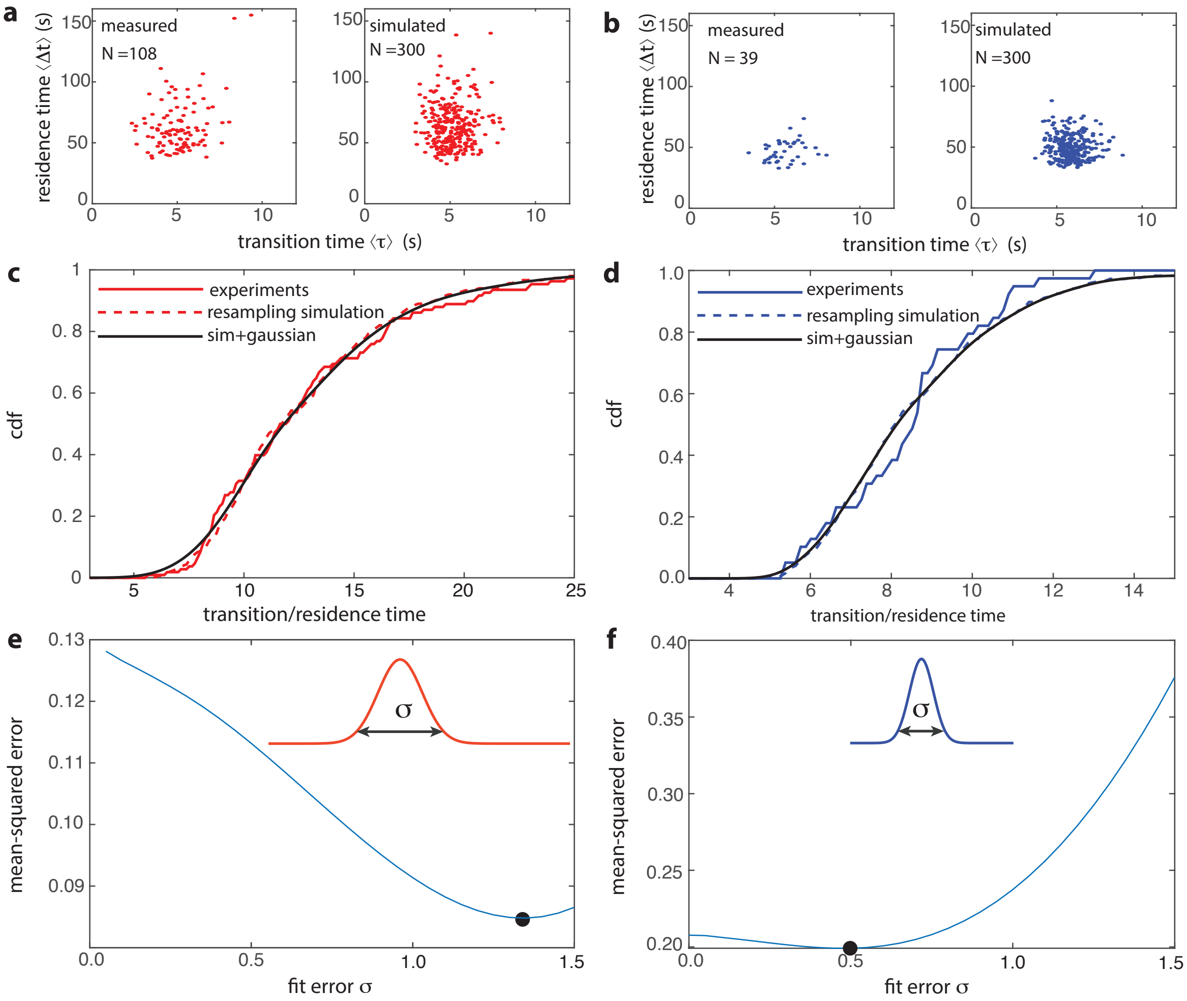

Figure **S8**: **Convolution-based estimation of true variation in residence/transition time ratio. (a)** Measured transition time vs residence time for (Left) 108 cells expressing Tsr-I214K from FRET experiments, with activity $0.45 \leq \langle a \rangle \leq 0.55$, and (Right) for 300 simulated cells. For each simulated cell, residence times and transition times were drawn respectively from an exponential and gamma distribution with parameters extracted from the experiments (Figs. 2 and Extended Data Figure 6), where the sum of residence times was limited to the experimental duration (1000 s). The variation in the simulation represents the sampling error that is observed without variation in parameters between cells. **(b)** As in (a) but for cells expressing Tar [QEEE]. **(c)** The cumulative density function (cdf) for the distribution of transition/residence time ratio per cell in experiments (solid red line) and in the simulated data (dashed line). The simulated cdf (sampling noise) was convolved with a Gaussian pdf (cell–cell variation in ratio) with variance σ^2, representing true cell–cell variation in timescale ratio, and superimposed on the curve (black line, $\sigma = 1.3$.) **(d)** As in (c) but then for cells expressing Tar [QEEE] ($\sigma = 0.5$). **(e)** Mean squared error (mse) between the convolved cdf (resampling cdf $*$ gaussian pdf) and the experimental cdf, as a function of the standard deviation σ. The value of σ that minimizes the mse represents the cell–cell variation in timescale ratio that best describes the experimentally observed variation. **(f)** As in (e) but for cells expressing Tar [QEEE].

References

1. Y. Shin and C. P. Brangwynne, “Liquid phase condensation in cell physiology and disease,” *Science*, vol. 357, no. 6357, p. 4382, 2017.

2. S. F. Banani, H. O. Lee, A. A. Hyman, and M. K. Rosen, “Biomolecular condensates: organizers of cellular biochemistry,” *Nat. Rev. Mol. Cell Biol.*, vol. 18, no. 5, pp. 285–298, 2017.

3. L.-P. Bergeron-Sandoval, N. Safaee, and S. W. Michnick, “Mechanisms and Consequences of Macromolecular Phase Separation,” *Cell*, vol. 165, pp. 1067–1079, May 2016.

4. H. Wu, “Higher-order assemblies in a new paradigm of signal transduction,” *Cell*, vol. 153, no. 2, pp. 287–292, 2013.

5. H. Wu and M. Fuxreiter, “The structure and dynamics of higher-order assemblies: amyloids, signalosomes, and granules,” *Cell*, vol. 165, no. 5, pp. 1055–1066, 2016.

6. E. Korkmazhan, P. Tompa, and A. R. Dunn, “The role of ordered cooperative assembly in biomolecular condensates,” *Nat. Rev. Mol. Cell Biol.*, vol. 22, no. 10, pp. 647–648, 2021.

7. X. Su, J. A. Ditlev, E. Hui, W. Xing, S. Banjade, J. Okrut, D. S. King, J. Taunton, M. K. Rosen, and R. D. Vale, “Phase separation of signaling molecules promotes t cell receptor signal transduction,” *Science*, vol. 352, no. 6285, pp. 595–599, 2016.

8. W. Y. Huang, S. Alvarez, Y. Kondo, Y. K. Lee, J. K. Chung, H. Y. M. Lam, K. H. Biswas, J. Kuriyan, and J. T. Groves, “A molecular assembly phase transition and kinetic proofreading modulate ras activation by sos,” *Science*, vol. 363, no. 6431, pp. 1098–1103, 2019.

9. A. Klosin, F. Oltsch, T. Harmon, A. Honigmann, F. Jülicher, A. A. Hyman, and C. Zechner, “Phase separation provides a mechanism to reduce noise in cells,” *Science*, vol. 367, no. 6476, pp. 464–468, 2020.

10. D. Bray and T. Duke, “Conformational spread: The propagation of allosteric states in large multiprotein complexes,” *Annu. Rev. Biophys. Biomol. Struct.*, vol. 33, pp. 53–73, 2004.

11. Z. Chen and M. B. Elowitz, “Programmable protein circuit design,” *Cell*, vol. 184, no. 9, pp. 2284–2301, 2021.

12. A. J. Ben-Sasson, J. L. Watson, W. Sheffler, M. C. Johnson, A. Bittleston, L. Somasundaram, J. Decarreau, F. Jiao, J. Chen, I. Mela, A. A. Drabek, S. M. Jarrett, S. C. Blacklow, C. F. Kaminski, G. L. Hura, J. J. De Yoreo, J. M. Kollman, H. Ruohola-Baker, E. Derivery, and D. Baker, “Design of biologically active binary protein 2d materials,” *Nature*, vol. 589, no. 7842, pp. 468–473, 2021.

13. A. Pillai, A. Idris, A. Philomin, C. Weidle, R. Skotheim, P. J. Y. Leung, A. Broerman, C. Demakis, A. J. Borst, F. Praetorius, and D. Baker, “De novo design of allosterically switchable protein assemblies,” *Nature*, 2024.

14. T. Kortemme, “De novo protein designFrom new structures to programmable functions,” *Cell*, vol. 187, pp. 526–544, Feb. 2024.

15. R. Ciuffa, T. Lamark, A. K. Tarafder, A. Guesdon, S. Rybina, W. J. Hagen, T. Johansen, and C. Sachse, “The selective autophagy receptor p62 forms a flexible filamentous helical scaffold,” *Cell reports*, vol. 11, no. 5, pp. 748–758, 2015.

16. X. Cai, J. Chen, H. Xu, S. Liu, Q.-X. Jiang, R. Halfmann, and Z. J. Chen, “Prion-like polymerization underlies signal transduction in antiviral immune defense and inflammasome activation,” *Cell*, vol. 156, no. 6, pp. 1207–1222, 2014.

17. J. Ruan, S. Xia, X. Liu, J. Lieberman, and H. Wu, “Cryo-em structure of the gasdermin a3 membrane pore,” *Nature*, vol. 557, no. 7703, pp. 62–67, 2018.

18. Y. Chang, K. Zhang, B. L. Carroll, X. Zhao, N. W. Charon, S. J. Norris, M. A. Motaleb, C. Li, and J. Liu, “Molecular mechanism for rotational switching of the bacterial flagellar motor,” *Nat. struct. & mol. biol.*, vol. 27, no. 11, pp. 1041–1047, 2020.

19. A. Briegel, D. Ortega, E. Tocheva, K. Wuichet, Z. Li, S. Chen, A. Mueller, C. Iancu, G. Murphy, M. J. Dobro, I. Zuhlin, and G. Jensen, “Universal architecture of bacterial chemoreceptor arrays,” *PNAS*, vol. 106, pp. 17181–86, 2009.

20. C.-C. Yin, L. G. DCruz, and F. A. Lai, “Ryanodine receptor arrays: not just a pretty pattern?,” *Trends in cell biology*, vol. 18, no. 4, pp. 149–156, 2008.

21. É. S. Vanamee and D. L. Faustman, “Structural principles of tumor necrosis factor superfamily signaling,” *Science Signaling*, vol. 11, no. 511, p. eaao4910, 2018.

22. L. Pan, T.-M. Fu, W. Zhao, L. Zhao, W. Chen, C. Qiu, W. Liu, Z. Liu, A. Piai, Q. Fu, S. Chen, H. Wu, and J. J. Chou, “Higher-Order Clustering of the Transmembrane Anchor of DR5 Drives Signaling,” *Cell*, vol. 176, no. 6, pp. 1477–1489.e14, 2019.

23. Q. Yin, T.-M. Fu, J. Li, and H. Wu, “Structural Biology of Innate Immunity,” *Annual Review of Immunology*, vol. 33, pp. 393–416, Mar. 2015.

24. A. Briegel and G. Jensen, “Progress and Potential of Electron Cryotomography as Illustrated by Its Application to Bacterial Chemoreceptor Arrays,” *Ann. Rev. of Biophys.*, vol. 46, pp. 1–21, May 2017.

25. H. N. Motlagh, J. O. Wrabl, J. Li, and V. J. Hilser, “The ensemble nature of allostery,” *Nature*, vol. 508, pp. 331–339, Apr. 2014.

26. M. Beck, R. Covino, I. Hnelt, and M. Mller-McNicoll, "Understanding the cell: Future views of structural biology," *Cell*, vol. 187, pp. 545–562, Feb. 2024.

27. E. Nogales and J. Mahamid, "Bridging structural and cell biology with cryo-electron microscopy," *Nature*, vol. 628, pp. 47–56, Apr. 2024.

28. V. Sourjik and H. Berg, "Receptor sensitivity in bacterial chemotaxis.," *PNAS*, vol. 99, no. 1, pp. 123–127, 2002.

29. S. Bi and V. Sourjik, "Stimulus sensing and signal processing in bacterial chemotaxis," *Current Opinion in Microbiology*, vol. 45, pp. 22–29, Oct. 2018.

30. J. M. Keegstra, F. Carrara, and R. Stocker, "Ecological roles of bacterial chemotaxis," *Nature Reviews Microbiology*, vol. 20, pp. 491–504, 2022.

31. P. Zhang, C. M. Khursigara, L. M. Hartnell, and S. Subramaniam, "Direct visualization of escherichia coli chemotaxis receptor arrays using cryo-electron microscopy," *PNAS*, vol. 104, no. 10, pp. 3777–3781, 2007.

32. A. Briegel, X. Li, A. Bilwes, K. Hughes, G. Jensen, and B. Crane, "Bacterial chemoreceptor arrays are hexagonally packed trimers of receptor dimers networked by rings of kinase and coupling proteins," *PNAS*, vol. 109, p. 3767, 2012.

33. J. Liu, B. Hu, D. R. Morado, S. Jani, M. D. Manson, and W. Margolin, "Molecular architecture of chemoreceptor arrays revealed by cryoelectron tomography of escherichia coli minicells," *PNAS*, vol. 109, no. 23, pp. E1481–E1488, 2012.

34. J. M. Keegstra, K. Kamino, F. Anquez, M. Lazova, T. Emonet, and T. S. Shimizu, "Phenotypic diversity and temporal variability in a bacterial signaling network revealed by single-cell fret," *eLife*, vol. 6, p. e27455, 2017.

35. R. Colin, C. Rosazza, A. Vaknin, and C. Sourjik, "Multiple sources of slow activity fluctuations in a bacterial chemosensory network," *eLife*, vol. 6, p. e26796, 2017.

36. D. Greenfield, A. L. McEvoy, H. Shroff, G. E. Crooks, N. S. Wingreen, E. Betzig, and J. Liphardt, "Self-organization of the escherichia coli chemotaxis network imaged with super-resolution light microscopy," *PLoS biology*, vol. 7, no. 6, p. e1000137, 2009.

37. M. Koler, E. Peretz, C. Aditya, T. S. Shimizu, and A. Vaknin, "Long-term positioning and polar preference of chemoreceptor clusters in e. coli," *Nature communications*, vol. 9, no. 1, pp. 1–10, 2018.

38. E. Korobkova, T. Emonet, J. M. Vilar, T. Shimizu, and P. Cluzel, "From molecular noise to behavioural variability in a single bacterium.," *Nature*, vol. 428, no. 6982, pp. 574–578, 2004.

39. T. Shimizu, Y. Tu, and H. Berg, “A modular gradient-sensing network for chemotaxis in Escherichia coli revealed by responses to time-varying stimuli.,” *Mol. Sys. Biol.*, vol. 6, June 2010.

40. S. Kitanovic, P. Ames, and J. Parkinson, “Mutational analysis of the control cable that mediates transmembrane signaling in the escherichia coli serine chemoreceptor,” *J Bacteriol*, vol. 193, no. 19, pp. 5062–5072, 2011.

41. R. Phillips, “The molecular switch,” in *The Molecular Switch*, Princeton University Press, 2020.

42. V. Sourjik and H. C. Berg, “Localization of components of the chemotaxis machinery of *Escherichia coli* using fluorescent protein fusions,” *Mol. Microb.*, vol. 37, pp. 740–751, Aug. 2000.

43. B. J. Cantwell, R. R. Draheim, R. B. Weart, C. Nguyen, R. C. Stewart, and M. D. Manson, “Chez phosphatase localizes to chemoreceptor patches via chea-short,” *J. Bacteriol.*, vol. 185, no. 7, pp. 2354–2361, 2003.

44. T. Duke and D. Bray, “Heightened sensitivity of a lattice of membrane receptors,” *PNAS*, vol. 96, pp. 10104–8, 1999.

45. B. Mello, L. Shaw, and Y. Tu, “Effects of receptor interaction in bacterial chemotaxis,” *Biophys. J.*, vol. 87, pp. 1578–95, 2004.

46. M. Skoge, R. Endres, and N. Wingreen, “Receptor-Receptor coupling in bacterial chemotaxis: Evidence for strongly coupled clusters,” *Biophys. J.*, vol. 90, pp. 4317–4326, June 2006.

47. M. Skoge, Y. Meir, and N. Wingreen, “Dynamics of cooperativity in chemical sensing among cell-surface receptors,” *Phys. Rev. Lett.*, vol. 107, p. 178101, 2011.

48. V. Sourjik and H. Berg, “Functional interactions between receptors in bacterial chemotaxis.,” *Nature*, vol. 428, no. 6981, pp. 437–441, 2004.

49. B. Mello and Y. Tu, “An allosteric model for heterogeneous receptor complexes: Understanding bacterial chemotaxis responses to multiple stimuli,” *PNAS*, vol. 102, no. 48, pp. 17354–17359, 2005.

50. R. Endres and N. Wingreen, “Precise adaptation in bacterial chemotaxis through assistance neighbourhoods,” *PNAS*, vol. 103, pp. 13040–44, 2006.

51. Y. Tu, T. Shimizu, and H. Berg, “Modeling the chemotactic response of Escherichia coli to time-varying stimuli,” *PNAS*, vol. 105, pp. 14855–14860, Sept. 2008.

52. F. Bai, R. Branch, D. Nicolau, T. Pilizota, B. Steel, P. Maini, and R. Berry, "Conformational spread as a mechanism for cooperativity in the bacterial flagellar switch.," *Science*, vol. 327, pp. 685–9, 2010.

53. F. Wang, H. Shi, R. He, R. Wang, R. Zhang, and J. Yuan, "Non-equilibrium effect in the allosteric regulation of the bacterial flagellar switch," *Nature Physics*, 2017.

54. Y. Pomeau, "Symétrie des fluctuations dans le renversement du temps," *Journal de Physique*, vol. 43, no. 6, pp. 859–867, 1982.

55. M. Zanin and D. Papo, "Algorithmic approaches for assessing irreversibility in time series: Review and comparison," *Entropy*, vol. 23, no. 11, p. 1474, 2021.

56. M. A. Muoz, "*Colloquium* : Criticality and dynamical scaling in living systems," *Reviews of Modern Physics*, vol. 90, p. 031001, July 2018.

57. M. E. Fisher and M. N. Barber, "Scaling theory for finite-size effects in the critical region," *Phys. Rev. Lett.*, vol. 28, pp. 1516–1519, Jun 1972.

58. P. C. Hohenberg and B. I. Halperin, "Theory of dynamic critical phenomena," *Reviews of Modern Physics*, vol. 49, no. 3, p. 435, 1977.

59. H. Takano, "Finite-Size Scaling Approach to the Kinetic Ising Model," *Progress of Theoretical Physics*, vol. 68, pp. 493–507, Aug. 1982.

60. S. Miyashita and H. Takano, "Dynamical Nature of the Phase Transition of the Two-Dimensional Kinetic Ising Model," *Progress of Theoretical Physics*, vol. 73, pp. 1122–1140, May 1985.

61. A. E. Ferdinand and M. E. Fisher, "Bounded and inhomogeneous ising models. i. specific-heat anomaly of a finite lattice," *Physical Review*, vol. 185, no. 2, p. 832, 1969.

62. D. P. Landau, "Finite-size behavior of the Ising square lattice," *Physical Review B*, vol. 13, pp. 2997–3011, Apr. 1976.

63. M. Meijers, S. Ito, and P. R. ten Wolde, "Behavior of information flow near criticality," *Phys. Rev. E*, vol. 103, p. L010102, Jan 2021.

64. J. Segall, S. Block, and H. Berg, "Temporal comparisons in bacterial chemotaxis," *PNAS*, pp. 8987–8991, 1986.

65. D. A. Clark and L. C. Grant, "The bacterial chemotactic response reflects a compromise between transient and steady-state behavior," *PNAS*, vol. 102, no. 26, pp. 9150–9155, 2005.

66. Y. S. Dufour, X. Fu, L. Hernandez-Nunez, and T. Emonet, "Limits of feedback control in bacterial chemotaxis," *PLoS computational biology*, vol. 10, no. 6, p. e1003694, 2014.

67. P. Ames, C. Studdert, R. Reiser, and J. Parkinson, "Collaborative signaling by mixed chemoreceptor teams in escherichia coli," *PNAS*, vol. 99, pp. 7060–65, 2002.

68. H. Salman and A. Libchaber, "A concentration-dependent switch in the bacterial response to temperature.," *Nature Cell Biology*, vol. 9, no. 9, pp. 1098–1100, 2007.

69. Y. Kalinin, S. Neumann, V. S., and M. Wu, "Responses of Escherichia coli bacteria to two opposing chemoattractant gradients depend on the chemoreceptor ratio," *J. Bacteriol.*, vol. 192, no. 7, pp. 1796–1800, 2010.

70. R. E. Steuer, *Multiple Criteria Optimization: Theory, Computation, and Application*. Wiley, 1986.

71. J. Segall, M. Manson, and H. Berg, "Signal processing times in bacterial chemotaxis.," *Nature*, vol. 296, pp. 855–7, 1982.

72. V. Sourjik and H. Berg, "Binding of the Escherichia coli response regulator CheY to its target measured in vivo by fluorescence resonance energy transfer.," *PNAS*, vol. 99, no. 20, pp. 12669–12674, 2002.

73. M. Levin, T. Shimizu, and D. Bray, "Binding and diffusion of CheR molecules within a cluster of membrane receptors," *Biophys. J.*, vol. 82, pp. 1809–17, 2002.

74. T. Shimizu, S. Aksenov, and D. Bray, "A spatially extended stochastic model of the bacterial chemotaxis signalling pathway.," *J. Mol. Biol.*, vol. 329, no. 2, pp. 291–309, 2003.

75. Y. Tu and G. Grinstein, "How white noise generates power-law switching in bacterial flagellar motors," *Phys. Rev. Lett.*, vol. 94, p. 208101, 2005.

76. H. Huo, R. He, R. Zhang, and J. Yuan, "Swimming escherichia coli cells explore the environment by lévy walk," *Applied and environmental microbiology*, vol. 87, no. 6, pp. e02429–20, 2021.

77. N. Figueroa-Morales, R. Soto, G. Junot, T. Darnige, C. Douarche, V. A. Martinez, A. Lindner, and É. Clément, "3d spatial exploration by e. coli echoes motor temporal variability," *Physical Review X*, vol. 10, no. 2, p. 021004, 2020.

78. A. C. Costa, G. Sridhar, C. Wyart, and M. Vergassola, "Fluctuating landscapes and heavy tails in animal behavior," *PRX Life*, vol. 2, no. 2, p. 023001, 2024.

79. T. Emonet and P. Cluzel, "Relationship between cellular response and behavioral variability in bacterial chemotaxis," *PNAS*, vol. 105, pp. 3304–3309, Mar. 2008.

80. D. Clausznitzer and R. Endres, "Noise characteristics of the escherichia coli rotary motor," *BMC Systems Biology*, vol. 5, p. 151, 2011.

81. T. Mora, A. M. Walczak, W. Bialek, and C. G. Callan Jr, "Maximum entropy models for antibody diversity," *PNAS*, vol. 107, no. 12, pp. 5405–5410, 2010.

82. S. L. Veatch, P. Cicuta, P. Sengupta, A. Honerkamp-Smith, D. Holowka, and B. Baird, "Critical fluctuations in plasma membrane vesicles," *ACS chemical biology*, vol. 3, no. 5, pp. 287–293, 2008.

83. J. W. Larkin, X. Zhai, K. Kikuchi, S. E. Redford, A. Prindle, J. Liu, S. Greenfield, A. M. Walczak, J. Garcia-Ojalvo, A. Mugler, and G. M. Sel, "Signal Percolation within a Bacterial Community," *Cell Systems*, vol. 7, pp. 137–145.e3, Aug. 2018.

84. J. Hesse and T. Gross, "Self-organized criticality as a fundamental property of neural systems," *Front. Sys. Neur.*, vol. 8, Sept. 2014.

85. A. J. T. M. Mathijssen, J. Culver, M. S. Bhamla, and M. Prakash, "Collective intercellular communication through ultra-fast hydrodynamic trigger waves," *Nature*, vol. 571, pp. 560–564, July 2019.

86. A. Cavagna, A. Cimarelli, I. Giardina, G. Parisi, R. Santagati, F. Stefanini, and M. Viale, "Scale-free correlations in starling flocks," *PNAS*, vol. 107, no. 26, pp. 11865–11870, 2010.

87. T. Mora and W. Bialek, "Are Biological Systems Poised at Criticality?," *Journal of Statistical Physics*, vol. 144, pp. 268–302, July 2011.

88. D. Hathcock, Q. Yu, B. A. Mello, D. N. Amin, G. L. Hazelbauer, and Y. Tu, "A nonequilibrium allosteric model for receptor-kinase complexes: The role of energy dissipation in chemotaxis signaling," *PNAS*, vol. 120, no. 42, p. e2303115120, 2023.

89. D. Hathcock, Q. Yu, and Y. Tu, "Time-reversal symmetry breaking in the chemosensory array reveals a general mechanism for dissipation-enhanced cooperative sensing," *Nat. Comm.*, no. 8892, 2024.

90. D. M. Sherry, I. R. Graf, S. J. Bryant, T. Emonet, and B. B. Machta, "Lattice ultrasensitivity produces large gain in *E. coli* chemosensing," *preprint at https://doi.org/10.1101/2024.05.28.596300*, 2024.

91. K. Kamino, J. M. Keegstra, J. Long, T. Emonet, and T. S. Shimizu, "Adaptive tuning of cell sensory diversity without changes in gene expression," *Science Advances*, vol. 6, no. 46, p. eabc1087, 2020.

92. K. Taute, S. Gude, S. Tans, and T. Shimizu, “High-throughput 3D tracking of bacteria on a standard phase contrast microscope,” *Nat. Comm.*, vol. 6, 2015.

93. K. A. Datsenko and B. L. Wanner, “One-step inactivation of chromosomal genes in ¡i¿escherichia coli¡/i¿ k-12 using pcr products,” *PNAS*, vol. 97, no. 12, pp. 6640–6645, 2000.

94. J. S. Parkinson, “Complementation analysis and deletion mapping of Escherichia coli mutants defective in chemotaxis,” *J. Bacteriol.*, vol. 135, pp. 45–53, July 1978.

95. O. Oleksiuk, V. Jakovljevic, N. Vladimirov, R. Carvalho, E. Paster, W. Ryu, Y. Meir, N. Wingreen, M. Kollmann, and V. Sourjik, “Thermal robustness of signaling in bacterial chemotaxis,” *Cell*, vol. 145, no. 2, pp. 312–321, 2011.

96. H. C. Berg and S. M. Block, “A Miniature Flow Cell Designed for Rapid Exchange of Media Under High-power Microscope Objectives,” *Journal of General Microbiology*, vol. 130, pp. 2915–2920., 1984.

97. P. Thévanas, U. Ruttimann, and M. Unser, “A pyramid approach to subpixel registration based on intensity,” ieee transactions on image processing,” *IEEE Transactions on Image Processing*, vol. 7, pp. 27–41, 1998.

98. L. Coelho, “Mahotas: Open source software for scriptable computer vision.,” *Journal of Open Research Software*, vol. 1, p. e3, 2013.

99. S. Schulmeister, M. Ruttorf, S. Thiem, D. Kentner, D. Lebiedz, and V. Sourjik, “Protein exchange dynamics at chemoreceptor clusters in Escherichia coli,” *PNAS*, vol. 105, pp. 6403–6408, Apr. 2008.

100. A. Vaknin and H. Berg, “Single Cell FRET imaging of phosphatase activity in the escherichia coli chemotaxis system.,” *PNAS*, vol. 101, no. 49, pp. 170272–7, 2004.

101. A. B. Bortz, M. H. Kalos, and J. L. Lebowitz, “A new algorithm for monte carlo simulation of ising spin systems,” *Journal of Computational Physics*, vol. 17, no. 1, pp. 10–18, 1975.

102. J. Parkinson, G. Hazelbauer, and J. Falke, “Signaling and sensory adaptation in escherichia coli chemoreceptors: 2015 update,” *Trends in Microbiology*, vol. 23, no. 5, pp. 257–266, 2015.

103. M. Li and G. L. Hazelbauer, “Cellular stoichiometry of the components of the chemotaxis signaling complex.,” *J. Bacteriol.*, vol. 186, pp. 3687–3694, June 2004.

104. T. Terwilliger, J. Wang, and D. Koshland, “Kinetics of receptor modification,” *J. Biol. Chem.*, vol. 261, pp. 10814–20, 1986.

105. S. A. Simms, A. M. Stock, and J. B. Stock, "Purification and Characterization of the S-Adenosy1methionine:Glutamyl Methyltransferase That Modifies Membrane Chemoreceptor Proteins in Bacteria," *The Journal of Biological Chemistry*, vol. 262, no. 18, pp. 8537–8543, 1987.

106. D. V. Schroeder, *Introduction to Thermal Physics*. Addison Wesley Longman, 2000.

107. J. M. Keegstra, F. Avgidis, *et al.*, "Figshare dataset.".

108. S. Bi, M. Kargeti, R. Colin, N. Farke, H. Link, and V. Sourjik, "Dynamic fluctuations in a bacterial metabolic network," *Nat. Comm.*, vol. 14, p. 2173, Apr. 2023.

109. X. Han and J. Parkinson, "An unorthodox sensory adaptation site in the e. coli serine chemoreceptor," *J. Bacteriol.*, vol. 196, pp. 641–649, 2014.

110. H. Tajima, K. Imada, M. Sakuma, F. Hattori, T. Nara, K. Noaki, M. Homma, and I. Kawagishi, "Ligand specificity determined by differentially arranged common ligand-binding residues in bacterial amino acid chemoreceptors tsr and tar.," *J. Biol. Chem.*, vol. 286, pp. 42200–42210, 2011.

111. C. Wolff and J. Parkinson, "Aspartate Taxis Mutants of the Escherichia coli Tar Chemoreceptor," *J. BACTERIOL.*, vol. 170, p. 7, 1988.

112. E. Ising, "Beitrag zur theorie des ferromagnetismus," *Z. Physik*, vol. 31, p. 253, 1925.

113. J.-P. Changeux, "50 years of allosteric interactions: the twists and turns of the models," *Nature Reviews Molecular Cell Biology*, vol. 14, pp. 819–829, Dec. 2013.

114. L. Onsager, "Crystal Statistics. I. A Two-Dimensional Model with an Order-Disorder Transition," *Physical Review*, vol. 65, pp. 117–149, Feb. 1944.

115. L. P. Kadanoff, "Scaling laws for ising models near t c," *Physics Physique Fizika*, vol. 2, no. 6, p. 263, 1966.

116. J. Cardy, *Finite-size scaling*. Elsevier, 1988.

117. A. Papoulis, *Probability, Random Variables, and Stochastic Processes*. New York: McGraw-Hill, 2nd ed., 1984.

118. K. Binder and D. P. Landau, "Finite-size scaling at first-order phase transitions," *Physical Review B*, vol. 30, pp. 1477–1485, Aug. 1984.

119. T. A. Duke, N. Le Novère, and D. Bray, "Conformational spread in a ring of proteins: a stochastic approach to allostery," *J. Mol. Biol.*, vol. 308, pp. 541–53, 2001.

120. J. Keymer, R. Endres, M. Skoge, Y. Meir, and N. Wingreen, "Chemosensing in Escherichia coli: Two regimes of two-state receptors," *PNAS*, vol. 103, pp. 1786–1791, Feb. 2006.

121. M. E. Fisher, "The theory of equilibrium critical phenomena," *Reports on progress in physics*, vol. 30, no. 2, p. 615, 1967.

122. C. K. Cassidy, B. A. Himes, D. Sun, J. Ma, G. Zhao, J. S. Parkinson, P. J. Stansfeld, Z. Luthey-Schulten, and P. Zhang, "Structure and dynamics of the e. coli chemotaxis core signaling complex by cryo-electron tomography and molecular simulations," *Communications biology*, vol. 3, no. 1, pp. 1–10, 2020.

123. M. Li and G. Hazelbauer, "Core unit of chemotaxis signaling complexes," *PNAS*, vol. 108, pp. 9390–5, 2011.

124. G. Piñas, V. Frank, A. Vaknin, and J. Parkinson, "The source of high signal cooperativity in bacterial chemosensory arrays," *PNAS*, vol. 113, no. 12, pp. 3335–3340, 2016.

125. R. G. Smock and L. M. Gierasch, "Sending Signals Dynamically," *Science*, vol. 324, pp. 198–203, Apr. 2009.

126. J. Lewandowski, M. Halse, M. Blackledge, and L. Emsley, "Direct observation of hierarchical protein dynamics," *Science*, vol. 348, pp. 587–581, May 2015.

127. Y. Goldtzvik, M. L. Mugnai, and D. Thirumalai, "Dynamics of Allosteric Transitions in Dynein," *Structure*, vol. 26, pp. 1664–1677.e5, Dec. 2018.

128. D. D. Boehr, D. McElheny, H. J. Dyson, and P. E. Wright, "Millisecond timescale fluctuations in dihydrofolate reductase are exquisitely sensitive to the bound ligands," *PNAS*, vol. 107, pp. 1373–1378, Jan. 2010.

129. S. Buchenberg, F. Sittel, and G. Stock, "Time-resolved observation of protein allosteric communication," *PNAS*, vol. 114, pp. E6804–E6811, Aug. 2017.

130. M. N. Levit, Y. Liu, and J. B. Stock, "Mechanism of CheA Protein Kinase Activation in Receptor Signaling Complexes," *Biochemistry*, vol. 38, pp. 6651–6658, May 1999.

131. A. B. Harris, "Effect of random defects on the critical behaviour of ising models," *Journal of Physics C: Solid State Physics*, vol. 7, no. 9, p. 1671, 1974.

132. S. Cho and M. P. A. Fisher, "Criticality in the two-dimensional random-bond ising model," *Phys. Rev. B*, vol. 55, pp. 1025–1031, Jan 1997.

133. Y. Imry and S.-k. Ma, "Random-field instability of the ordered state of continuous symmetry," *Phys. Rev. Lett.*, vol. 35, pp. 1399–1401, Nov 1975.

134. G. E. Piñas, M. D. DeSantis, C. K. Cassidy, and J. S. Parkinson, “Hexameric rings of the scaffolding protein chew enhance response sensitivity and cooperativity in escherichia coli chemoreceptor arrays,” *Science Signaling*, vol. 15, no. 718, p. eabj1737, 2022.

135. P. Cluzel, M. Surette, and S. Leibler, “An ultrasensitive bacterial motor revealed by monitoring signaling proteins in single cells,” *Science*, vol. 287, pp. 1652–1655, Mar. 2000.

136. J. Yuan and H. Berg, “Ultrasensitivity of an adaptive bacterial motor,” *J Mol Biol.*, vol. 425, pp. 1760–4, 2013.

137. G. Grinstein, C. Jayaprakash, and Y. He, “Statistical mechanics of probabilistic cellular automata,” *Physical review letters*, vol. 55, no. 23, p. 2527, 1985.

138. U. C. Täuber, V. K. Akkineni, and J. E. Santos, “Effects of violating detailed balance on critical dynamics,” *Physical review letters*, vol. 88, no. 4, p. 045702, 2002.

139. D. R. Ortega, C. Yang, P. Ames, J. Baudry, J. S. Parkinson, and I. B. Zhulin, “A phenylalanine rotameric switch for signal-state control in bacterial chemoreceptors,” *Nat. Comm.*, vol. 4, pp. 1–8, 2013.

140. L. M. Guzman, D. Belin, M. J. Carson, and J. Beckwith, “Tight regulation, modulation, and high-level expression by vectors containing the arabinose PBAD promoter,” *J. Bacteriol.*, vol. 177, pp. 4121–4130, July 1995.
