# Supplementary material for: Spontaneous switching in a protein signalling array reveals near-critical cooperativity
Source: Nat Phys. 2026 Jan 29;22(3):452–60. doi: 10.1038/s41567-025-03158-3 (PMC13019169; doi:10.1038/s41567-025-03158-3)
Supplement: Supplementary file 2 — Reporting Summary [file 41567_2025_3158_MOESM2_ESM.pdf]

nature portfolio

Corresponding author(s): Shimizu, Thomas S.

Last updated by author(s): September 17, 2025

Reporting Summary

Nature Portfolio wishes to improve the reproducibility of the work that we publish. This form provides structure for consistency and transparency in reporting. For further information on Nature Portfolio policies, see our Editorial Policies and the Editorial Policy Checklist.

Statistics

For all statistical analyses, confirm that the following items are present in the figure legend, table legend, main text, or Methods section.

n/a	Confirmed	
☐	☒	The exact sample size (*n*) for each experimental group/condition, given as a discrete number and unit of measurement
☐	☒	A statement on whether measurements were taken from distinct samples or whether the same sample was measured repeatedly
☐	☒	The statistical test(s) used AND whether they are one- or two-sided<br>*Only common tests should be described solely by name; describe more complex techniques in the Methods section.*
☒	☐	A description of all covariates tested
☒	☐	A description of any assumptions or corrections, such as tests of normality and adjustment for multiple comparisons
☒	☐	A full description of the statistical parameters including central tendency (e.g. means) or other basic estimates (e.g. regression coefficient) AND variation (e.g. standard deviation) or associated estimates of uncertainty (e.g. confidence intervals)
☒	☐	For null hypothesis testing, the test statistic (e.g. *F*, *t*, *r*) with confidence intervals, effect sizes, degrees of freedom and *P* value noted<br>*Give P values as exact values whenever suitable.*
☒	☐	For Bayesian analysis, information on the choice of priors and Markov chain Monte Carlo settings
☒	☐	For hierarchical and complex designs, identification of the appropriate level for tests and full reporting of outcomes
☐	☒	Estimates of effect sizes (e.g. Cohen's *d*, Pearson's *r*), indicating how they were calculated

Our web collection on statistics for biologists contains articles on many of the points above.

Software and code

Policy information about availability of computer code

Data collection	Simulated data on Ising lattices were generated using stochastic simulations with Python (v 3.7.11). FRET microscopy images were aquired using custom microscopy control software.
Data analysis	Experimental and simulated data analyses were performed using custom scripts in MATLAB (2017A or newer) and Python (v 3.7.11). Custom scripts are available in a Figshare repository: https://doi.org/10.6084/m9.figshare.27824757.

For manuscripts utilizing custom algorithms or software that are central to the research but not yet described in published literature, software must be made available to editors and reviewers. We strongly encourage code deposition in a community repository (e.g. GitHub). See the Nature Portfolio guidelines for submitting code & software for further information.

Data

Policy information about availability of data

All manuscripts must include a data availability statement. This statement should provide the following information, where applicable:

- Accession codes, unique identifiers, or web links for publicly available datasets
- A description of any restrictions on data availability
- For clinical datasets or third party data, please ensure that the statement adheres to our policy

All FRET experimental data, including figure source data, are available in a Figshare repository: https://doi.org/10.6084/m9.figshare.27824757. Raw FRET images are available upon request. Bacterial strains and plasmids are listed respectively in Supplementary Tables 1 and 2 and are available upon request.

Research involving human participants, their data, or biological material

Policy information about studies with human participants or human data. See also policy information about sex, gender (identity/presentation), and sexual orientation and race, ethnicity and racism.

Reporting on sex and gender	Not applicable (no human participants or data).
Reporting on race, ethnicity, or other socially relevant groupings	Not applicable (no human participants or data).
Population characteristics	Not applicable (no human participants or data).
Recruitment	Not applicable (no human participants or data).
Ethics oversight	Not applicable (no human participants or data).

Note that full information on the approval of the study protocol must also be provided in the manuscript.

Field-specific reporting

Please select the one below that is the best fit for your research. If you are not sure, read the appropriate sections before making your selection.

☒ Life sciences ☐ Behavioural & social sciences ☐ Ecological, evolutionary & environmental sciences

For a reference copy of the document with all sections, see nature.com/documents/nr-reporting-summary-flat.pdf

Life sciences study design

All studies must disclose on these points even when the disclosure is negative.

Sample size	Sample sizes were not predetermined. The number of cells and number of events are reported in the figure captions or supplementary tables. The effect of limited sample size on estimation of switching statistics is addressed in Fig. S8.
Data exclusions	No data were excluded from the study a priori. When the analysis is confired to a subset of the data this is clearly stated in the manuscript, and the selection procedure were routinely executed. As explained in the main text, the study focuses on the switching statistics of activity timeseries with two activity states, which were in total 202/1414 (tar) and 548/1414 (Tsr) cells.
Replication	The experiments were successfully reproduced and data collected from 19 (Tar) and 44 (Tsr) independent FRET experiments. The number of replicates is mentioned in the figure captions.
Randomization	Randomization was not applicable in this study, as all samples were associated with different variables (mostly the chemoreceptor species).
Blinding	Blinding was not possible in this study as the person performing and analysing the data was the same.

Reporting for specific materials, systems and methods

We require information from authors about some types of materials, experimental systems and methods used in many studies. Here, indicate whether each material, system or method listed is relevant to your study. If you are not sure if a list item applies to your research, read the appropriate section before selecting a response.

Materials & experimental systems

n/a	Involved in the study	
☒	☐	Antibodies
☒	☐	Eukaryotic cell lines
☒	☐	Palaeontology and archaeology
☒	☐	Animals and other organisms
☒	☐	Clinical data
☒	☐	Dual use research of concern
☒	☐	Plants

Methods

n/a	Involved in the study	
☒	☐	ChIP-seq
☒	☐	Flow cytometry
☒	☐	MRI-based neuroimaging

Plants

Seed stocks

Report on the source of all seed stocks or other plant material used. If applicable, state the seed stock centre and catalogue number. If plant specimens were collected from the field, describe the collection location, date and sampling procedures.

Novel plant genotypes

Describe the methods by which all novel plant genotypes were produced. This includes those generated by transgenic approaches, gene editing, chemical/radiation-based mutagenesis and hybridization. For transgenic lines, describe the transformation method, the number of independent lines analyzed and the generation upon which experiments were performed. For gene-edited lines, describe the editor used, the endogenous sequence targeted for editing, the targeting guide RNA sequence (if applicable) and how the editor was applied.

Authentication

Describe any authentication procedures for each seed stock used or novel genotype generated. Describe any experiments used to assess the effect of a mutation and, where applicable, how potential secondary effects (e.g. second site T-DNA insertions, mosiacism, off-target gene editing) were examined.
